# In vivo CRISPR screens identify CBX4 as an epigenetic regulator for cancer immunotherapy

**DOI:** 10.1172/JCI200564

**Published:** 2026-03-31

**Authors:** Zhibo Ma, Wenlong Jia, Xi Zhou, Jing Liu, Qingwen Li, Ruizhi Chang, Gu Shiqi, Naonao Yuan, Zhishui Chen, Peixiang Lan

**Affiliations:** 1 Institute of Organ Transplantation, Tongji Hospital, Tongji Medical College, Huazhong University of Science and Technology, Wuhan, China.; 2 Key Laboratory of Organ Transplantation, Ministry of Education, NHC Key Laboratory of Organ Transplantation, Key Laboratory of Organ Transplantation, Chinese Academy of Medical Sciences, Wuhan, China.; 3 Hepatic Surgery Center, Tongji Hospital, Tongji Medical College, Huazhong University of Science and Technology, Wuhan, China.

**Keywords:** Immunology, Oncology, Cancer immunotherapy, Epigenetics, Macrophages

## Abstract

Epigenetic dysregulation is associated with immune evasion and immune checkpoint blockade (ICB) resistance. Here, using in vivo CRISPR/Cas9 screens targeting epigenetics-related factors in mouse tumor models treated with ICB, we identified chromobox 4 (CBX4) as a key negative regulator of the immune tumor microenvironment (TME). Single-cell RNA-seq and spatial transcriptomics analyses of patients receiving neoadjuvant anti–programmed cell death protein 1 (anti–PD-1) therapy revealed high CBX4 expression in both tumor cells and immunosuppressive tumor-associated macrophage subpopulations, with preferential accumulation in nonresponders. Deficiency of CBX4 in macrophages or tumor cells induced robust antitumor immunity and increased infiltration and the cytotoxic activity of CD8^+^ T cells and NK cells, thereby heightening the sensitivity of ICB treatment. Mechanistically, CBX4 targeted H3K9me3- and H3K27me3-marked endogenous retroelements such as RLTR4-Mm-int. Loss of CBX4 derepressed retrotransposons, activating cytosolic RNA-sensing pathways and triggering the type I IFN response, ultimately leading to a robustly inflamed TME. Moreover, we uncovered a negative correlation between CBX4 expression, immune responses, and retrotransposon levels, and were able to determine the prognosis of patients with hepatocellular carcinoma (HCC) undergoing ICB therapy. Our study establishes CBX4 as an epigenetic immune checkpoint through the epigenetic silencing of retrotransposons, remodeling the immune TME and thus providing a promising therapeutic target to enhance tumor immunogenicity and overcome immunotherapy resistance.

## Abstract 2

CRISPR screening of mouse tumor models identifies CBX4 as an epigenetic regulator of tumor immunity

## Graphical abstract

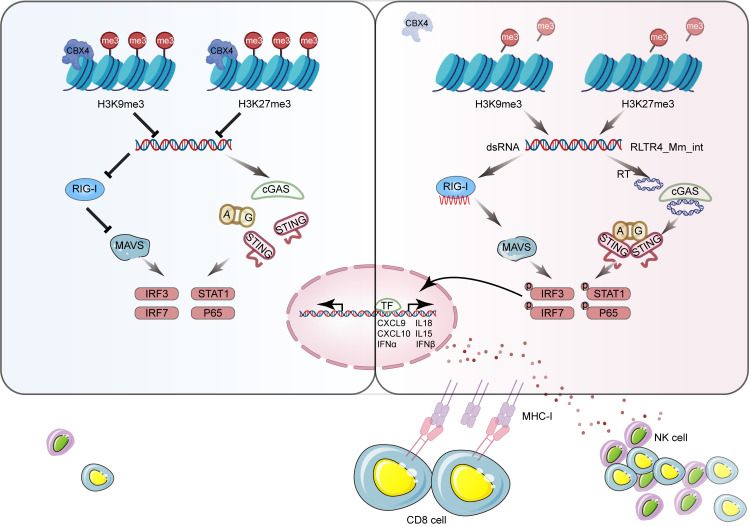

## Introduction

Immunotherapy, particularly immune checkpoint blockade (ICB), which elicits T cell responses, is now a mainstay approach for treating multiple cancers (1–3). Despite high durable response rates with cancer immunotherapies, many patients either do not benefit from these therapies (i.e., primary resistance) or relapse after a period of response (i.e., acquired resistance), underscoring the urgent need to identify cellular and molecular targets for advanced immunotherapies (2, 4, 5). The clinical efficacy of ICB therapy hinges on the proper functioning of CD8^+^ T cells and the orderly cell-cell interactions within the tumor microenvironment (TME) (6–9), which are crucial for initiating and sustaining T cell responses, fostering durable memory T cell formation, overcoming resistance, and facilitating desired outcomes. However, T cells do not operate autonomously in their effector functions (6). The onset and maintenance of T cell responses is intricately regulated by innate immune responses (10–12). The role of innate immune responses in the activation of T cells involves complex signaling pathways, including cytokine production and receptor-ligand interactions (13–15). Strategies that enhance the innate immune responses can synergize with ICB therapy to bolster the effector functions of CD8^+^ T cells and improve overall cancer treatment outcomes (16–19). Therefore, it is crucial to identify immunomodulatory targets or combinations that are regulated by immune stress, enhance tumor-intrinsic immunogenicity, and potentiate immunotherapy efficacy.

Emerging evidence suggests that, under immune pressure, tumors acquire traits disrupting antitumor immunity through highly plastic epigenetic reprogramming, resulting in immune invasion and ICB therapy resistance (20). Epigenetic reprogramming, including DNA methylation, histone modifications, and noncoding RNA regulation, are heritable changes in gene expression that do not involve alterations to the underlying DNA sequence (21). Understanding and targeting these epigenetic crosstalk pathways provide unique opportunities for selective therapeutic targeting. However, the complexity and heterogeneity of epigenetic alterations within tumors pose a formidable challenge to identify the core epigenetic driving factors. The advent of CRISPR/Cas9 screening has provided a robust tool for the unbiased and systematic identification of cancer cell vulnerabilities (22–25). Because genome-wide screens require a huge number of cells for adequate coverage, focused screens including a smaller set of tens to thousands of genes achieves higher sensitivity (25). We therefore conducted rigorous in vivo screen using tumor-bearing mouse models to systematically identify key epigenetic regulators that modulate the immune TME and subsequently influence the efficacy of ICB therapy.

In this study, we identified chromobox 4 (CBX4) as an epigenetic target involved in immune evasion and resistance to immunotherapy, which accumulated in nonresponders to anti–programmed cell death protein 1 (anti–PD-1) treatment in both tumor cells and in subpopulations of immunosuppressive tumor-associated macrophages. Targeting CBX4 derepressed H3K9me3- and H3K27me3-marked endogenous retroelements such as RLTR4-MM-int triggered the cytosolic RNA-sensing pathway and the subsequent type I IFN response, ultimately leading to a robustly inflamed TME. Our study establishes CBX4 as an epigenetic immune checkpoint through epigenetic silencing of retrotransposons and remodeling of the immune TME and identifies a promising therapeutic target to enhance tumor immunogenicity and overcome immunotherapy resistance.

## Results

### In vivo CRISPR screen identifies CBX4 as an epigenetic regulator of immune evasion.

**Figure 1:**
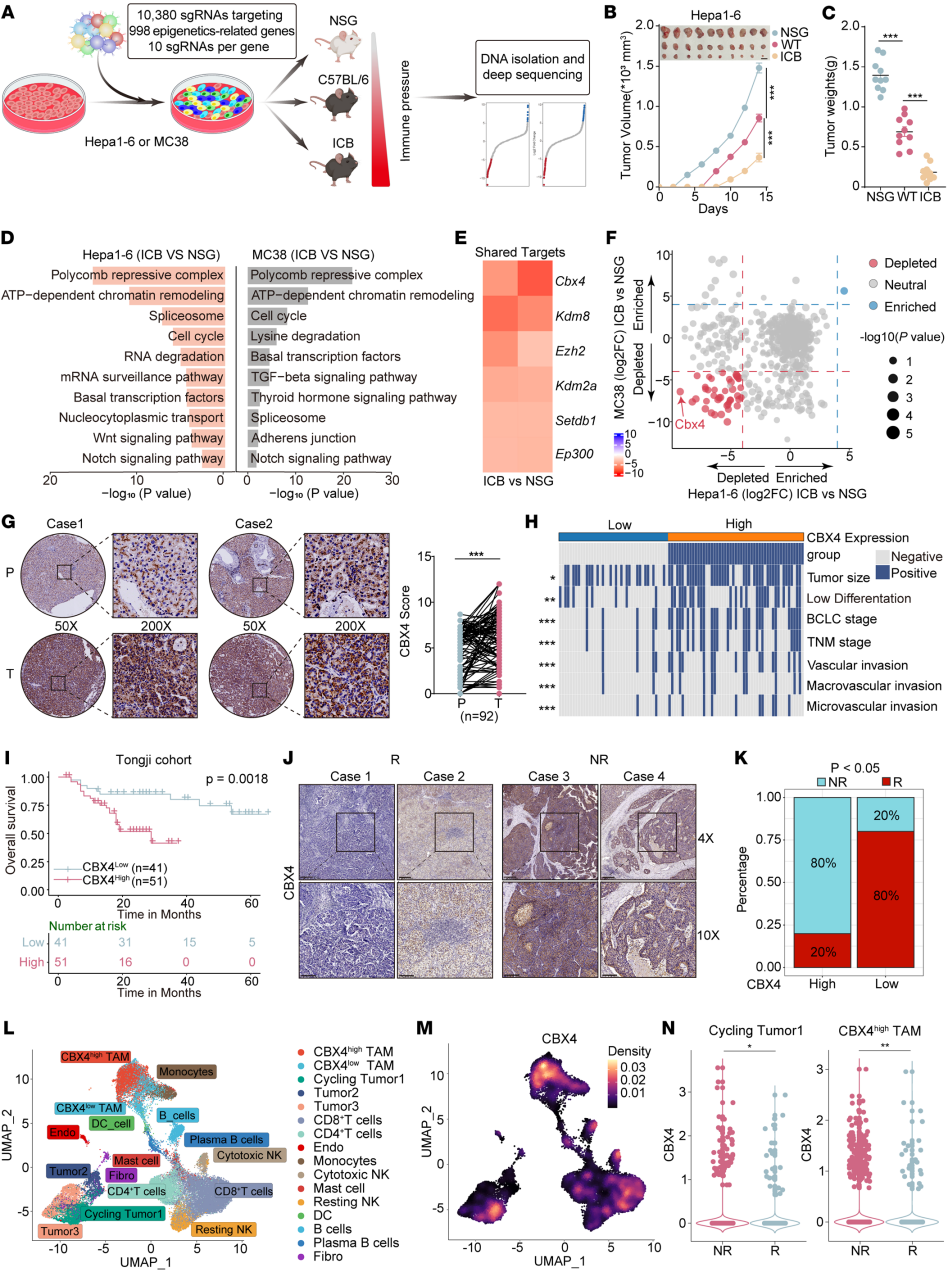
In vivo CRISPR screens identify CBX4 as an immune evasion target. (**A**) Schematic of the CRISPR screen. (**B** and **C**) Tumor growth curve (**B**) and tumor weights (**C**) for Hepa1-6 tumors (*n* = 10) from NSG, C57BL/6J, and C57BL/6J + ICB-treated groups. Scale bar: 1 cm. (**D**) Core pathway enrichment analysis of deleted genes in the Hepa1-6 and MC38 libraries from ICB-treated mice compared with NSG controls. (**E**) Depletion (red) and enrichment (blue) of targeted genes in ICB versus NSG mice grouped by top shared hits. (**F**) Comparison of ICB treatment and NSG mouse epigenetic library screening in Hepa1-6 and MC38 tumors. Red represents sgRNA depletion, blue represents enrichment, and the circle size corresponds to –log_10_(*P* value). (**G**) Representative IHC images of CBX4 staining of clinical HCC and paracancerous tissues (*n* = 92) and paired statistical analysis of staining intensity. Original magnification, ×50 and ×200. (**H**) Heatmap of clinicopathologic characteristics of Tongji cohort 2 HCC patients grouped by CBX4 expression level. BCLC, Barcelona Clinic Liver Cancer; TNM, tumor node metastasis. (**I**) Kaplan-Meier analyses of overall survival of mice according to CBX4 expression levels. (**J**) Representative IHC images showing CBX4 staining in clinical HCC samples (Tongji cohort 4). Original magnification, ×4 and ×10. (**K**) Relationship between high and low levels of CBX4 expression and the response rate to immunotherapy. (**L**) UMAP plots for identifying distinct single-cell clusters from RCC cohort samples after PD-1 blockade. (**M**) Feature plots showing the normalized gene expression of CBX4 projected onto the UMAP. Endo, endothelial cells: Fibro, fibroblasts. (**N**) Violin plots showing CBX4 expression distributions in the indicated subgroups of responders versus nonresponders. Data represent the mean ± SEM. *P < 0.05, **P < 0.01, and ***P < 0.001, by 2-way ANOVA with Tukey’s multiple-comparison test (**B**), 1-way ANOVA (**C**), 2-tailed, paired *t* test (**G**), 2-sided log-rank test (**I**), and 1-tailed, unpaired Wilcoxon test (**N**). NR, nonresponders; R, responders.

To systematically identify epigenetics-related immune evasion targets in tumor cells, we designed a lentiviral CRISPR/Cas9 screen library including 10 sgRNAs for each of 998 epigenetics-related genes for adequate coverage and high targeting efficiency (Supplemental Table 1; supplemental material available online with this article; https://doi.org/10.1172/JCI200564DS1), which were transduced into 2 mouse tumor cell lines, Hepa1-6 (hepatocellular carcinoma [HCC]) and MC38 (colon cancer). Tumor cells transduced with the sgRNA library were transplanted by injection into immunocompetent WT mice and ICB-treated WT mice, and immunodeficient NOD SCID *Il2rg^−/−^* (NSG) mice were used as controls to identify sgRNAs with immune-dependent effects (Figure 1A). After tumor collection, we performed library quality control and observed tumor growth inhibition driven by endogenous antitumor immunity or ICB therapy (Figure 1, B and C, and Supplemental Figure 1, A–D, and Supplemental Table 1). In addition to identifying genes such as *Ezh2*, *Setdb1*, *Ep300*, and *Mettl14*, which were previously discovered in library studies as being associated with ICB resistance, sgRNAs targeting the polycomb-repressive complex pathway were predominantly depleted in tumor cells under immune pressure (Figure 1, D and E, and Supplemental Figure 1, E–G) (22, 24). In validation experiments, KO of *Cbx4*, *Kdm8*, *Ezh2*, or *Kdm2a* sensitized tumors to anti–PD-1 in Hepa-1-6 tumor–bearing mice (Supplemental Figure 1, H–K). Notably, sgRNAs targeting Cbx4, a component of the canonical polycomb-repressive complex, ranked among the most depleted, highlighting the pivotal role of Cbx4 in tumor immune escape (Figure 1F). Additionally, we identified several factors, many of which scored preferentially in either Hepa1-6 or MC38 cell lines. Hepa1-6–specific hits included *Sf3b5*, *Exosc3*, *Dr1*, *Exosc9*, *Eif3k*, *Dnmt1*, etc. MC38-specific hits included *Rbm17*, *Cul4a*, *Mettl1*, *Mettl14*, *Kat14*, *Ino80*, etc. These context-specific genes also merit further study (Supplemental Figure 1E).

To further investigate the clinical and pathological relevance of CBX4 in HCC progression, we assembled an in-house cohort of 108 patients with HCC (Tongji cohort 2, Supplemental Table 2). Excluding samples with dropout, we included a total of 92 paired HCC and adjacent normal tissue samples. We found that CBX4 expression was significantly higher in HCC tissue than in adjacent normal tissue and that its expression was higher in advanced-stage tumors than in early-stage tumors (Figure 1G and Supplemental Figure 2, and H). In parallel, comprehensive profiling of multiple clinical cohorts — including 50 stage-stratified patients with HCC (Tongji cohort 3, Supplemental Table 2) and data from The Cancer Genome Atlas (TCGA) and the Clinical Proteomic Tumor Analysis Consortium (CPTAC) — demonstrated that CBX4 was significantly upregulated in tumor tissues compared with adjacent nontumor tissues across multiple cancer types (Supplemental Figure 2, A–F). Further analysis revealed that high CBX4 expression was closely associated with more aggressive tumor phenotypes (Figure 1H). Prognostic evaluation incorporating IHC scores, transcriptomics data, proteomics data, immune cell infiltration, and tumor purity consistently indicated that elevated CBX4 expression correlates with poor clinical outcomes (Figure 1, G–I, and Supplemental Figure 2, A–M). Notably, both univariate and multivariate Cox regression analyses identified CBX4 as an independent risk factor, underscoring its potential utility in patient risk stratification (Supplemental Figure 2, N and O).

We next investigated the relationship between CBX4 and the efficacy of ICB. Clinically, high CBX4 expression was associated with resistance to immunotherapy across multiple cancer types, which included HCC (Tongji cohort 4) (Figure 1, J and K, and Supplemental Table 2), colorectal cancer, melanoma, and urothelial tumors (Supplemental Figure 3, A–C). To substantiate these associations, we analyzed single-cell RNA-seq (scRNA-seq) and spatial transcriptomics data on patients treated with neoadjuvant anti–PD-1 therapy (26), which revealed that CBX4 was highly expressed in tumor cells and subpopulations of immunosuppressive tumor-associated macrophages (Figure 1, L–N, and Supplemental Figure 3, D–N) and was significantly enriched in the tumor tissues of nonresponsders compared with responders (Figure 1N and Supplemental Figure 3F). We next performed transcription factor binding motif analysis using the JASPAR and Profiler of Multi-Omics data (PROMO) databases to identify potential regulators of Cbx4, pinpointing Maz, E2f1, Usf1, and Patz1 as top candidates (Supplemental Figure 4A). siRNA-mediated Maz knockdown suppressed Cbx4 expression in Hepa1-6 cells, whereas E2f1, Usf1, and Patz1 knockdown had no effect on Cbx4 expression under the same conditions (Supplemental Figure 4, B and C). Then, we utilized the JASPAR database to predict 3 Maz binding sites (P1, P2, P3) on the Cbx4 promoter region. Cut&Tag quantitative PCR (qPCR) confirmed the binding of Maz to P1 and P2 sites on the Cbx4 promoter (Supplemental Figure 4, D and E). Correlation analysis across multiple transcriptomics datasets revealed that MAZ had a positive correlation with CBX4 expression (Supplemental Figure 4, F and G). Subsequently, we collected tissue specimens from patients with HCC following immunotherapy. Western blot analysis revealed that in nonresponders to immunotherapy, the levels of MAZ and CBX4 were significantly elevated compared with responders (Supplemental Figure 4, H and I). Moreover, there was a significant positive correlation between the protein levels of CBX4 and MAZ (Supplemental Figure 4K). Collectively, these data suggest that CBX4, identified through in vivo CRISPR screening, is closely linked to tumor malignancy and resistance to immunotherapy in patients with cancer.

### Loss of CBX4 in tumor cells induces antitumor immunity mediated by CD8^+^ T cells and NK cells.

**Figure 2:**
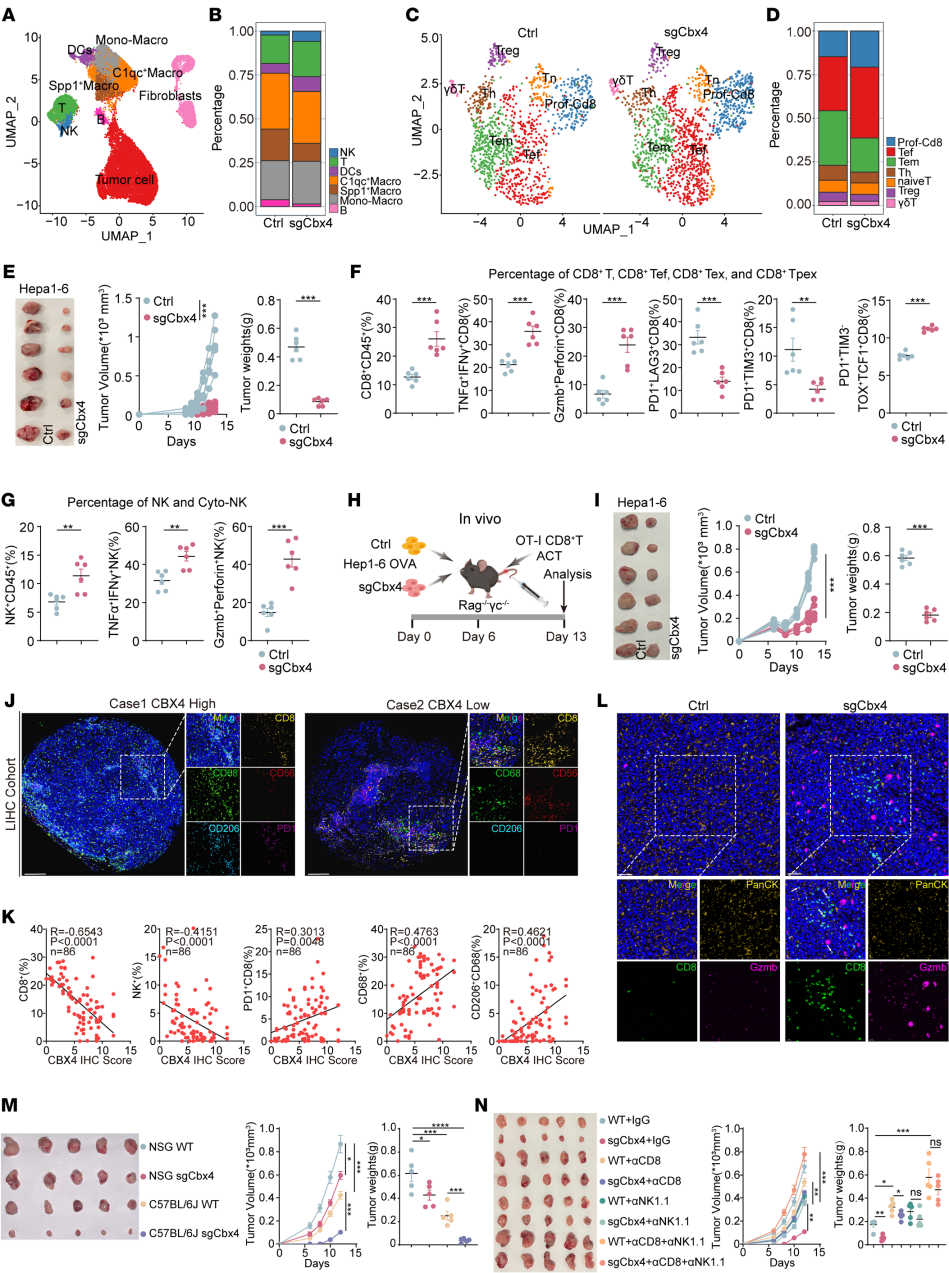
Loss of CBX4 in tumor cells augments CD8^+^ T cell and NK cell antitumor immunity. (**A**) UMAP of distinct cell clusters in control Hepa1-6 and sgCbx4 Hepa1-6 tumors. (**B**) Percentage of CD45^+^ immune cell populations. (**C**) UMAP of T cell subpopulations in control Hepa1-6 and sgCbx4 Hepa1-6 tumors. (**D**) Percentage of T cell subpopulations. (**E**) Image, tumor growth curves, and tumor volumes for Hepa1-6 tumors (*n* = 6). (**F** and **G**) Percentage of CD8^+^ T cells, CD8^+^ Tef cells, CD8^+^ Tex cells, CD8^+^ Tpex cells, NK cells, and cyto-NK cells in control and sgCbx4 Hepa1-6 tumors (*n* = 6). (**H**) Schematic of the adoptive T cell transfer experiment. (**I**) Image, tumor growth curves, and tumor volumes for Hepa1-6 tumors (*n* = 6). (**J**) Representative multiplex IHC images from Tongji cohort 2. CD68 (green), CD206 (azure), CD8 (yellow), CD56 (red), and PD-1 (purple). Scale bars: 200 μm; original magnification, ×8; ×24 (insets). (**K**) The clinical correlation between CBX4 expression and the density of CD8^+^ T cells, NK cells, PD-1^+^CD8^+^ T cells, CD68^+^ macrophages and CD206^+^CD68^+^ M2 macrophages from multiplex IHC and CBX4 IHC staining on the Tongji cohort 2 samples (*n* = 86). (**L**) Representative multiplex IHC images from control and sgCbx4 Hepa1-6 tumor tissues. CD8 (green), PanCK (yellow), Gzmb (purple). Scale bars: 50 μm; original magnification, ×20; ×40 (insets). (**M**) Image, tumor growth curves, and tumor volumes for Hepa1-6 tumors (*n* = 5). (**N**) Image, tumor growth curves, and tumor volumes for Hepa1-6 tumors (*n* = 5). Data represent the mean ± SEM. Tumor growth curve data were analyzed by 2-way ANOVA with Tukey’s multiple-comparison test (**E**, **I**, **M**, and **N**). Other data were analyzed by purity-corrected Spearman’s test (**K**), 1-way ANOVA (**M** and **N**), and 2-tailed, unpaired Student’s *t* test (**E**–**G** and **I**). *P < 0.05, **P < 0.01, ***P < 0.001, and ****P < 0.0001. Ctrl, control; Mono, monocytes; Macro, macrophages.

To comprehensively evaluate the effect of Cbx4 on the TME, we performed scRNA-seq on cells isolated from Hep1-6 control or Hep1-6 sgCbx4 tumor tissues in tumor-bearing mouse models (Figure 2A). Uniform manifold approximation and projection (UMAP) clustering identified 6 major cell types (Supplemental Figure 5A). Intratumoural CD45^+^ immune cell subsets demonstrated a significant increase in T cells, NK cells and DCs, alongside a marked decrease in Spp1^+^ macrophages in the tumors transduced with sgCbx4 (Figure 2B). To further investigate the effect of Cbx4 deficiency in tumor cells on T cell subpopulations, we conducted an additional dimensionality reduction analysis of T cells (Figure 2C). We classified T cells into 7 subpopulations, including effector T (Tef) cells, proliferating CD8^+^ T (CD8^+^ Tprof) cells, effector memory T (Tem) cells, Tregs, naive T cells, helper T (Th) cells, and γδT cells (Supplemental Figure 5B). Notably, the proportions of Tef cells with cytotoxic function and CD8^+^ Tprof cells significantly increased, alongside a marked decrease in Tregs in tumor tissues with Cbx4 ablation (Figure 2D). Loss of Cbx4 in tumor cells significantly inhibited Hepa1-6 and MC38 tumor growth (Figure 2E and Supplemental Figure 5L). Flow cytometric analysis validated the increased proportions and numbers of CD8^+^ T cells, CD8^+^ Tef cells (IFN-γ^+^TNF-α^+^ and perforin^+^granzyme B^+^), CD8^+^ progenitor of exhausted T (Tpex) cells (PD-1^+^TIM3^–^TOX^+^TCF1^+^), and NK and Cytotoxic Natural Killer (cyto-NK) cells (IFN-γ^+^TNF-α^+^ and perforin^+^granzyme B^+^), whereas that proportion of Exhausted T (Tex) cells was reduced (Figure 2, F and G, Supplemental Figure 5, C–K and M–T, and Supplemental Figure 6A). Consistent with these findings, we observed similar results in an orthotopic HCC tumor model (Supplemental Figure 7, A–G).

To elucidate the underlying mechanisms by which loss of Cbx4 in tumor cells elicits activation of the intratumoral immune microenvironment, we performed an analysis of tumor cell clusters from our single-cell sequencing datasets. Gene Ontology (GO) and gene set enrichment analysis (GSEA) analyses revealed that loss of Cbx4 induced the activation of innate immune pathways, including antigen processing and presentation of peptide antigen via MHC class I, responses to IFN-β, and positive regulation of the innate immune response. (Supplemental Figure 8, A and B). Next, we analyzed the expression of genes associated with T cell stimulation in sgCbx4 tumor cells. Tumor cells exhibited upregulated expression of antigen presentation machinery genes (*B2m, H2-D1*), which are critical for T cell survival and effector differentiation (27, 28). Additionally, *Cxcl10*, a chemokine essential for T and NK cell recruitment and linked to positive immunotherapy responses (29, 30), was also expressed at high levels (Supplemental Figure 8C). To further elucidate the effect of Cbx4 on the TME, we conducted a series of in vitro and in vivo assays. In coculturing experiments, OVA-specific CD8^+^ T cells displayed a significant increase in proliferation and effector cytokine production when incubated with OVA-expressing, Cbx4-deficient tumor cells rather than negative control tumor cells (Supplemental Figure 8, D–F). In addition, we subcutaneously implanted OVA-expressing Hepa1-6 cells into T, B, and NK cell–deficient Rag^–/–^ γc^–/–^ mice and simulated CD8^+^ T cell–mediated immune pressure via adoptive transfer of OVA-specific CD8^+^ T cells (Figure 2H). Cbx4-deficient tumors markedly enhanced the efficacy of adoptively transferred T cells in suppressing tumor growth (Figure 2I). Similar results were observed in cocultured naive NK cells with Hepa1-6 tumor cells (Supplemental Figure 8G). We confirmed that loss of Cbx4 in tumor cells also enabled NK cells to effectively differentiate into a cytotoxic phenotype (IFN-γ^+^TNF-α^+^ and perforin^+^granzyme B^+^) (Supplemental Figure 8H). Moreover, we constructed vector-tagBFP and Cbx4-Sh-tagGFP tumor cells and mixed at a 1:1 ratio for tumor rejection (Supplemental Figure 8I). Cbx4-Sh-tagGFP tumor cells were much more likely to get eradicated, and PD-1 blockade enhanced their vulnerabilities (Supplemental Figure 8J), in line with the observation in clinical immunotherapy cohorts and in an in vivo CRISPR screen showing that Cbx4^lo^ expression predicted better overall survival. Additionally, multiplex IHC revealed Cbx4 deficiency in tumor cells enhanced CD8^+^ T cell infiltration and cytotoxicity through more contact areas between tumor cells and CD8^+^ T cells in mice (Figure 2L). Consistently, multiplex IHC from the Tongji cohort 2 (Figure 2J) revealed a significant clinical negative correlation between CBX4 expression and the abundance of CD8^+^ T cells and NK cells (Figure 2K), while we observed a significant positive correlation between CBX4 expression and the presence of exhausted T cells and M2 macrophages (Figure 2K).

Given that Cbx4 ablation in tumors led to alterations in CD8^+^ T cells and NK cells, we first clarified whether the inhibitory role of sgCbx4 on tumor growth was dependent on immunity. Our data revealed that targeting Cbx4 only slightly inhibited tumor growth in NSG mice (Figure 2M). Consistent with previous studies, loss of Cbx4 can inhibit the proliferation of tumor cells (31). However, in immunocompetent C57BL/6J mice, targeting Cbx4 in tumor cells significantly suppressed tumor growth. These results showed that the antitumor efficacy of targeting Cbx4 in tumor cells was more dependent on the functions of immune cells. (Figure 2M). To explore which specific immune cell subsets are required during targeting of Cbx4-inhibited tumor progression, depleting antibodies were used to selectively deplete CD8^+^ T cells, NK cells, or CD8^+^ T cells/NK cells (Supplemental Figure 8K). Our results showed that depletion of CD8^+^ T cells and NK cells abolished the effect of Cbx4 ablation on tumor progression (Figure 2N). These findings suggest that targeting Cbx4 to suppress tumor growth dependent on CD8^+^ T cells and NK cells, which prompted us to concentrate on the mechanisms underlying Cbx4 promotion of tumor cell resistance to CD8^+^ T cell and NK cell–dependent cytotoxicity.

### CBX4 ablation in macrophages induces antitumor immunity through CD8^+^T cells and NK cells.

**Figure 3:**
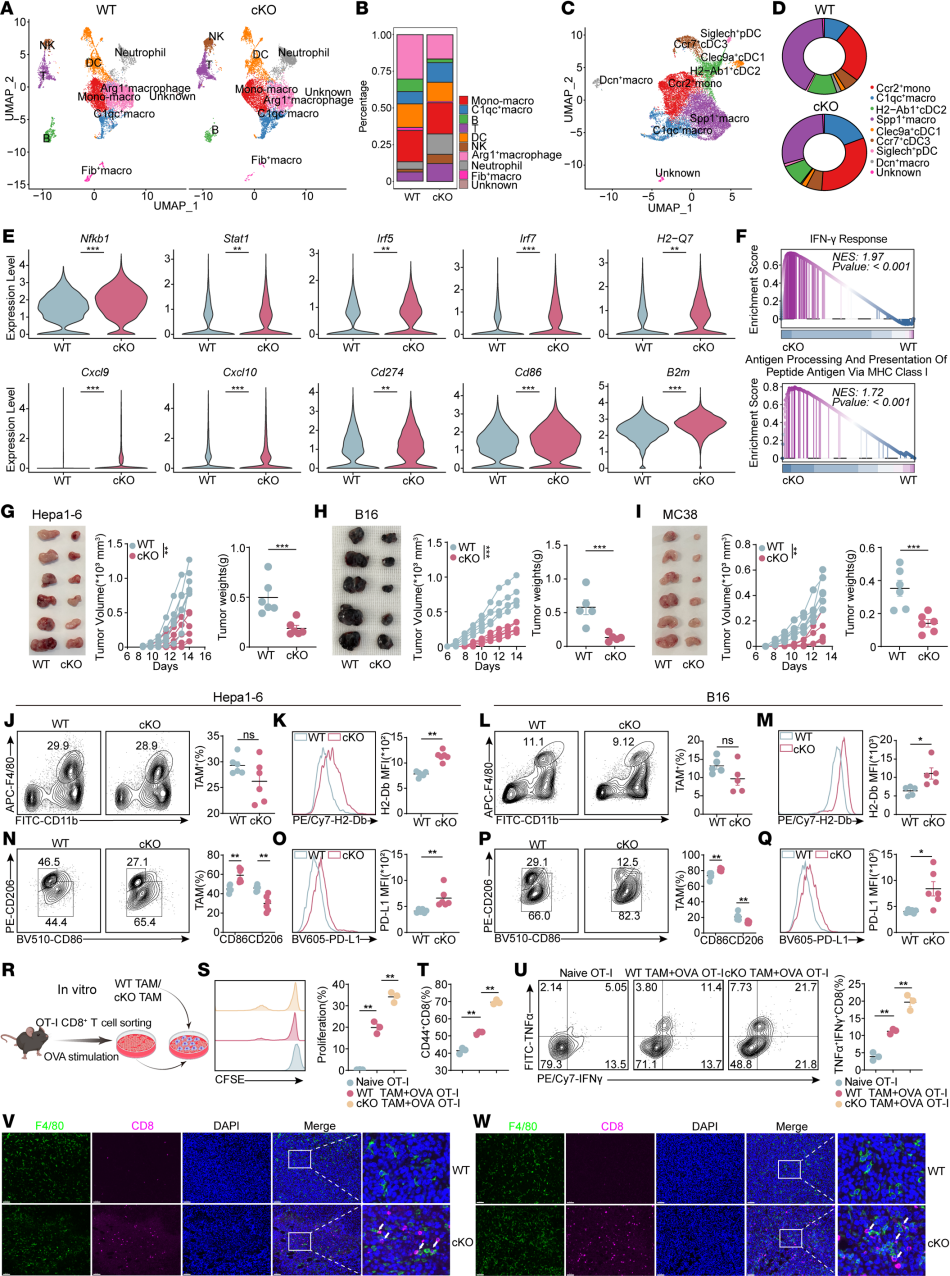
Depletion of CBX4 in macrophages induces an immunostimulatory phenotype. (**A**) UMAP of distinct clusters in WT Hepa1-6 and Cbx4-cKO Hepa1-6 tumors. (**B**) Percentage of cell populations. (**C**) UMAP of myeloid cell subpopulations in WT Hepa1-6 and Cbx4-cKO Hepa1-6 tumors. (**D**) Distribution of myeloid cell subpopulations. (**E**) Violin plots showing the expression distribution of innate immune–related genes in myeloid cells from the WT versus the Cbx4-cKO groups. (**F**) GSEA plots of the top 2 pathways induced by *Cbx4* deletion in myeloid cells from scRNA-seq. NES, normalized enrichment score. (**G**–**I**) Hepa1-6, B16, and MC38 tumor volumes, tumor growth curves, and tumor weights were quantified. (**J** and **L**) The percentage of CD11b^hi^F4/80^hi^ macrophages in WT and Cbx4-cKO Hepa1-6 (*n* = 6) and B16 tumors (*n* = 5). (**K** and **M**) MFI of histocompatibility 2, D region locus b (H2-Db) on CD11b^hi^F4/80^hi^ macrophages in WT and Cbx4-cKO Hepa1-6 (*n* = 6) and B16 tumors (*n* = 5). (**N** and **P**) Percentage of CD86^+^ and CD206^+^ on CD11b^hi^F4/80^hi^ macrophages in WT and Cbx4-cKO Hepa1-6 and B16 tumors (*n* = 6). (**O** and **Q**) MFI of PD-L1 on CD11b^hi^F4/80^hi^ macrophages in WT and Cbx4-cKO Hepa1-6 and B16 tumors (*n* = 6). (**R**–**U**) Schematic (**R**), representative CFSE proliferation analysis (**S**), and quantification of CD44^+^ (**T**) and TNF-α^+^IFN-γ^+^ (**U**) OVA-specific T cells cocultured with or without WT TAMs or Cbx4-cKO TAMs (*n* = 3). (**V** and **W**) Representative multiplex IHC images from WT and Cbx4-cKO Hepa1-6 and B16 tumor tissues. F4/80 (green), CD8 (purple), DAPI (blue). Scale bars: 50 μm; original magnification, ×20; ×80 (insets). Data represent the mean ± SEM. Tumor growth curves data were analyzed by 2-way ANOVA with Tukey’s multiple-comparison test (**G**–**I**). Other data were analyzed by 1-way ANOVA (**S**–**U**) and 2-tailed, unpaired Student’s *t* test (**G**–**Q**). *P < 0.05, **P < 0.01, and ***P < 0.001.

Based on the scRNA-seq and spatial transcriptomics of patients treated with neoadjuvant anti–PD-1 therapy, we observed that CBX4 was expressed at higher levels in the immunosuppressive TAM subpopulations within the tumor tissues of nonresponders, suggesting that CBX4 in macrophages may also play a critical role in immune evasion (Figure 1M and Supplemental Figure 3F). Immunofluorescence staining revealed a significant colocalization of CBX4 and macrophages in hepatocellular carcinoma tissues (Supplemental Figure 9A). Furthermore, the expression level of CBX4 was highly positively correlated with CD68 (a macrophage marker), and high CBX4 and CD68 expression levels were associated with poor clinical outcomes (Supplemental Figure 9, A–C). Consequently, we constructed *LysM^Cre^ Cbx4^fl/fl^* myeloid cell–conditional KO (cKO) mice. To comprehensively evaluate the effect of Cbx4 ablation in myeloid cells on the TME, we enriched CD45^+^ immune cells to perform scRNA-seq of cells isolated from Hep1-6 tumor tissues from WT and *LysM^Cre^ Cbx4^fl/fl^* mice (Figure 3A). We identified 10 distinct cell clusters, including T cells, NK cells, B cells, DCs, C1qc^+^ macrophages, Arg1^+^ macrophages, fibro-like macrophages, monocytes, and a subset of unknown cells (Supplemental Figure 10A). Comparative cluster dynamics analysis across conditions demonstrated a significant increase in T cells, NK cells, and C1qc^+^ macrophages (acknowledged as a M1-like subpopulation) (32), alongside a marked decrease in Arg1^+^ macrophages (acknowledged as a M2-like immunosuppressive subpopulation) (32) in the tumors of *LysM^Cre^ Cbx4^fl/fl^* mice (Figure 3B). To further investigate the effect of Cbx4 ablation on myeloid cells, we conducted an additional dimensionality reduction analysis of myeloid cells (Figure 3C). We classified myeloid cells into 9 subpopulations, including C1qc^+^ macrophages, Spp1^+^ macrophages, Ccr2^+^ macrophages, Dcn^+^ macrophages, Clec9a^+^ cDC1, H2-Ab1^+^ cDC2, Ccr7^+^ cDC3, Siglech^+^ plasmacytoid DCs (pDCs), and a subpopulation of unknown myeloid cells (Supplemental Figure 10B). Notably, the proportion of M1-like C1qc^+^ macrophages and Ccr2^+^ macrophages markedly increased, alongside a marked decrease in immunosuppressive Spp1^+^ macrophages (Figure 3D). However, the proportion of Clec9a^+^ cDC1, H2-Ab1^+^ cDC2, Ccr7^+^ cDC3, and Siglech^+^ pDCs showed only a faint shift (Figure 3D), and the expression level of Cbx4 in DCs was notably low (Supplemental Figure 3H), which indicated that ablation of Cbx4 primarily exerted its function through macrophage-related subpopulations.

Notably, analysis of differentially expressed genes (DEGs) and GSEA showed that the ablation of Cbx4 in macrophages promoted the activation of innate immune pathways (Figure 3, E and F). To validate our single-cell findings, we performed flow cytometry on mouse models of HCC, melanoma, and colorectal cancer. The ablation of Cbx4 in macrophages significantly inhibited tumor growth (Figure 3, G–I). Consistent with the single-cell findings, the flow cytometry results from HCC and melanoma cells from the *LysM^Cre^ Cbx4^fl/fl^* group showed the proportion of total tumor-associated macrophages did not change significantly (Figure 3, J and L). However, the proportion of CD86^+^ tumor-associated macrophages (TAMs) was substantially higher in the *LysM^Cre^ Cbx4^fl/fl^* group, along with a marked decrease in immunosuppressive CD206^+^ macrophages (Figure 3, N and P). Meanwhile, the expression of MHC class I molecules and programmed death ligand 1 (PD-L1) markedly increased (Figure 3, K, M, O, and Q). To elucidate the macrophages antigen presentation ability, we cocultured naive OT-I CD8^+^ T cells with WT or *LysM^Cre^ Cbx4^fl/fl^* bone marrow–derived macrophages (BMDMs) loaded with ova257-264 peptide. Consistently, coculture assays and flow cytometry showed that ablation of Cbx4 in TAMs resulted in a significant increase in proliferation and effector cytokine production (Figure 3, R–U). Additionally, multiplex IHC from Hep1-6 and B16 tumor tissues revealed that Cbx4 deficiency in macrophages enhanced CD8^+^ T cell infiltration through more contact areas between macrophages and CD8^+^ T cells, suggesting more cell-cell interactions and communication between macrophages and CD8^+^ T cells under Cbx4 ablation (Figure 3, V and W).

**Figure 4:**
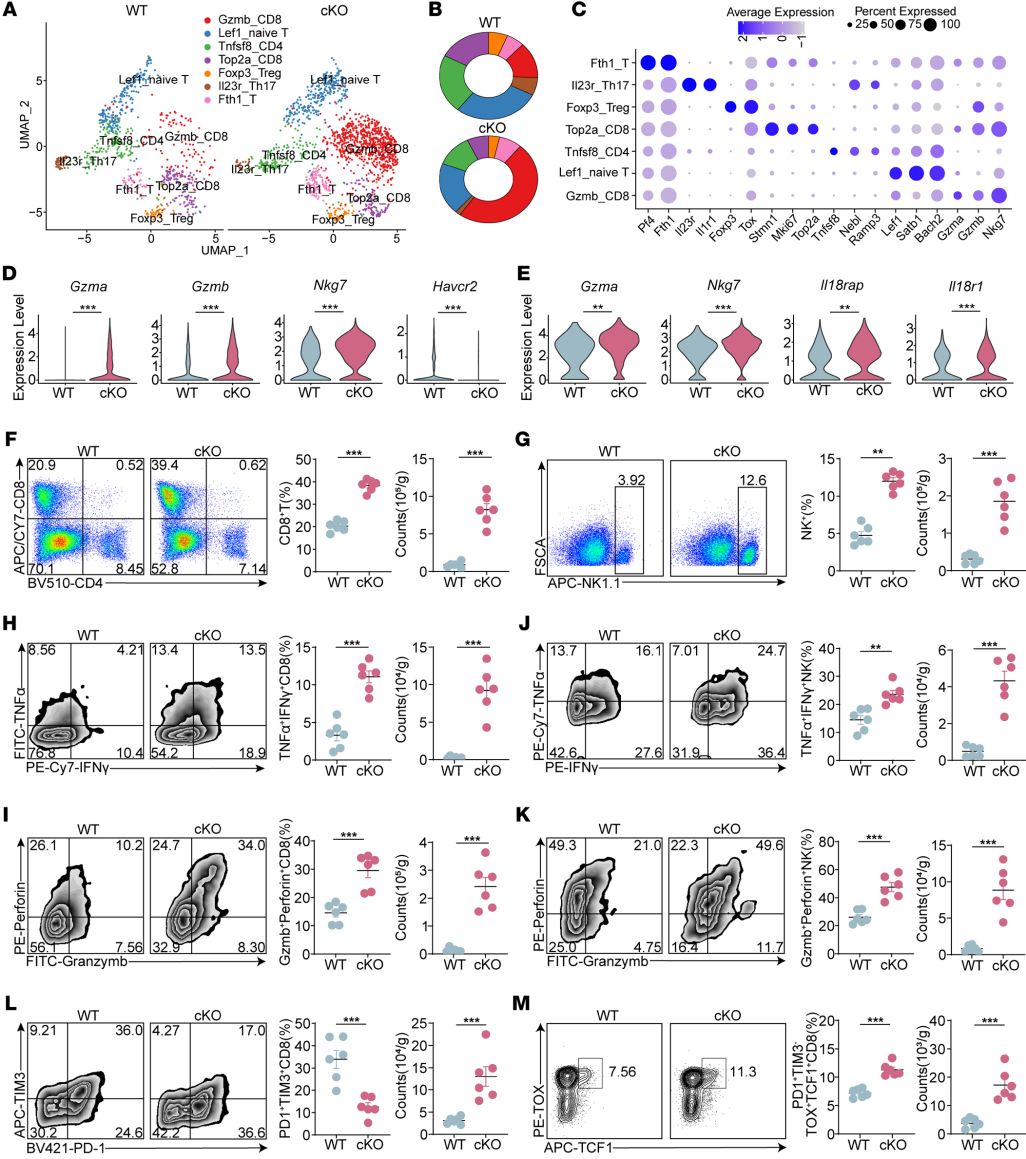
Depletion of CBX4 in macrophages augments CD8^+^ T cell and NK cell antitumor immunity. (**A**) UMAP of T cell subpopulations in WT Hepa1-6 and Cbx4-cKO Hepa1-6 tumors. (**B**) Distribution of T cell subpopulations. (**C**) Marker gene expression across defined cell clusters. Bubble size is proportional to the percentage of cells expressing a gene, and color intensity is proportional to average scaled gene expression. (**D**) Violin plots showing the expression distributions of cytotoxicity-related genes (*Gzma*, *Gzmb*, *Gzmk*, and *Nkg7*) and the exhaustion-related gene *Havcr2* from scRNA-seq of T cell subpopulations in WT Hepa1-6 and Cbx4-cKO Hepa1-6 tumors. (**E**) Violin plots showing the expression distributions of cytotoxicity-related genes (*Gzma*, *Gzmb*, and *Nkg7*) and NK cell activation–related genes (*Il18rap* and *Il18r1*) from scRNA-seq of NK cell subpopulations in WT Hepa1-6 and Cbx4-cKO Hepa1-6 tumors. (**F**–**M**) Percentage and number of CD8^+^ T cells (**F**), NK cells (**G**), TNF-α^+^IFN-γ^+^CD8^+^ T cells (**H**), perforin^+^granzyme B^+^CD8^+^ T cells (**I**), TNF-α^+^IFN-γ^+^ NK cells (**J**), perforin^+^granzyme B^+^ NK cells (**K**), PD-1^+^TIM3^+^CD8^+^ T cells (**L**), and PD-1^+^TIM3^–^TCF1^+^TOX^+^CD8^+^ T cells (**M**) in WT and Cbx4-cKO Hepa1-6 tumors (*n* = 6). Data represent the mean ± SEM. *P < 0.05, **P < 0.01, and ***P < 0.001, by 2-tailed, unpaired Student’s *t* test (**F**–**M**).

To further investigate the effect of Cbx4 deficiency in macrophages on T cell subpopulations, we conducted an additional dimensionality reduction analysis of T cells (Figure 4A). We classified T cells into 7 subpopulations, including Gzmb^+^CD8^+^ T cells (Tef cells), Top2^+^ CD8^+^ T cells (proliferating CD8^+^ T cells), Tnfsf8^+^ T cells, Tregs, Lef1^+^ T cells (naive T cells), IL-23r^+^ Th17 cells, and Fth1^+^ T cells (Figure 4C). Notably, the proportion and number of effector CD8^+^ T cells with cytotoxic function were markedly increased, along with a marked decrease in Tregs in the tumors of *LysM^Cre^ Cbx4^fl/fl^* mice (Figure 4B). DEGs in T cells and NK cells showed that the ablation of Cbx4 in macrophages remodeled an inflammatory TME (Figure 4, D and E). Flow cytometry results from HCC and melanoma cells validated the increased proportion and number of CD8^+^ T cells (Figure 4F and Supplemental Figure 11A), CD8^+^ Tef cells (IFN-γ^+^TNF-α^+^ and perforin^+^granzyme B^+^) (Figure 4, H and I, and Supplemental Figure 11, C and D), CD8^+^ Tpex cells (PD-1^+^TIM3^–^TOX^+^TCF1^+^) (Figure 4M), NK cells (Figure 4G and Supplemental Figure 11B), and cyto-NK cells (Figure 4, J and K, and Supplemental Figure 11, E and F), whereas the proportion of Tex cells (PD-1^+^TIM3^+^ and PD-1^+^LAG3^+^) was reduced (Figure 4L and Supplemental Figure 11, G and H). Consistent with these findings, we observed similar results in an orthotopic HCC tumor model (Supplemental Figure 12, A–G). Additionally, multiplex IHC revealed that Cbx4 deficiency in macrophages enhanced CD8^+^ T cell and NK cell infiltration and cytotoxicity (Supplemental Figure 11I). Given that Cbx4 ablation in macrophages led to alterations in CD8^+^ T cells and NK cells, we further clarified the role of these specific immune cell subsets by selectively depleting CD8^+^ T cells, NK cells, or both CD8^+^ T cells and NK cells (Supplemental Figure 11J). Our results showed that depletion of CD8^+^ T cells and NK cells abolished the effect of Cbx4-ablated macrophages on tumor progression (Supplemental Figure 11, J and K). These findings indicate that Cbx4 ablation in macrophages promoted antitumor immunity in a CD8^+^ T cell– and NK cell–dependent manner, which may reflect a mechanism similar to that of Cbx4 deficiency in tumor cells.

### CBX4 ablation triggers antiviral immune responses through activation of RIG-I/IFN signaling.

**Figure 5:**
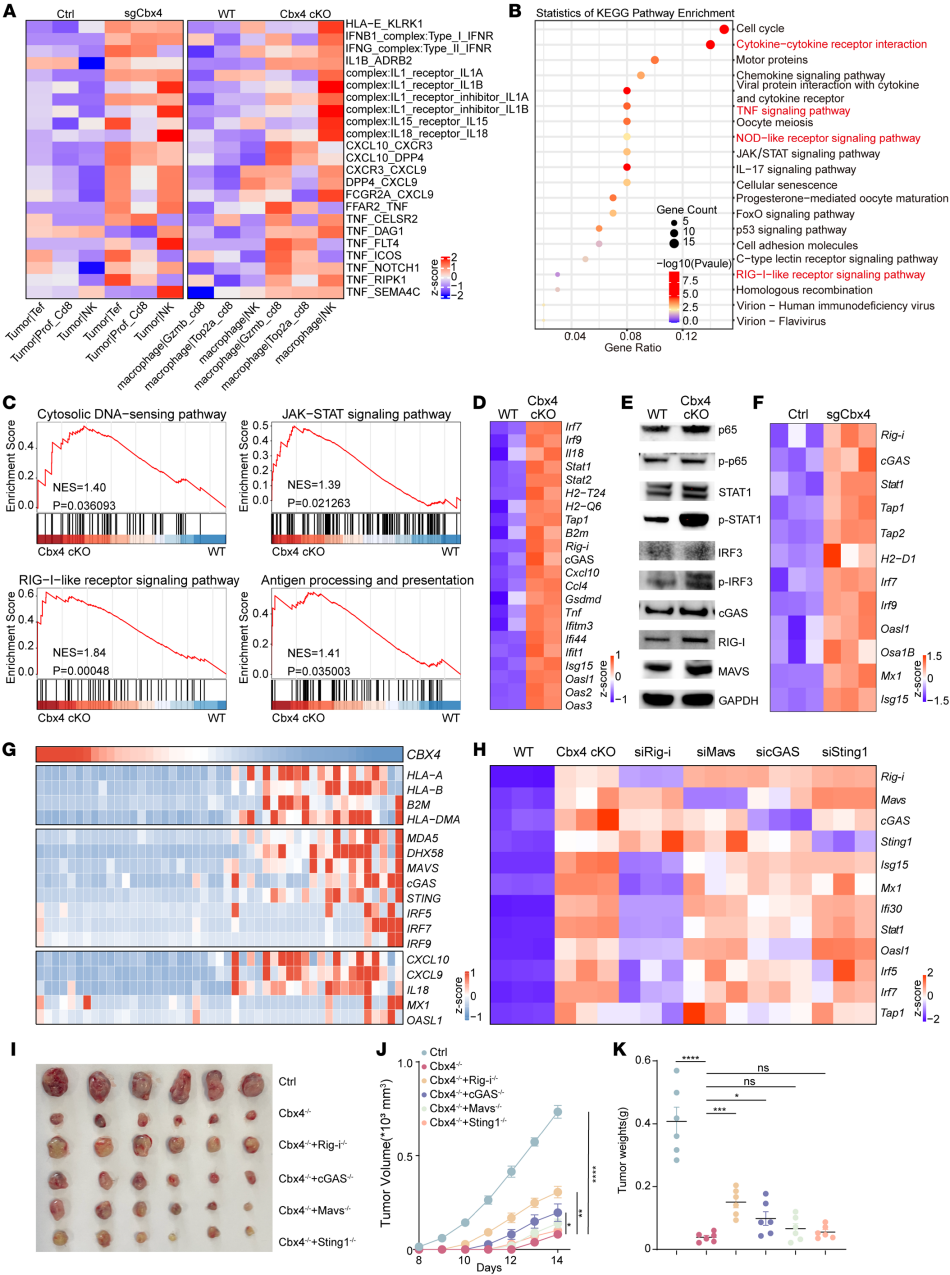
Depletion of CBX4 activates the type I IFN response through cytosolic RNA–sensing pathway. (**A**) Heatmaps summarize the changes in the strength of cell-cell interactions mediated by chemokines and cytokines following the ablation of Cbx4 in tumor cells and macrophages. (**B**) KEGG analysis of RNA-seq data from Cbx4-cKO CD11b^hi^F4/80^hi^ TAMs versus WT CD11b^hi^F4/80^hi^ TAMs. CD11b^hi^F4/80^hi^ TAMs were isolated by FACS. (**C**) GSEA plots of 4 top pathways induced by Cbx4 deletion in CD11b^hi^F4/80^hi^ TAMs from RNA-seq. NES, normalized enrichment score. (**D**) Heatmap of scaled type I IFN–related gene expression and innate immune–related gene expression between WT CD11b^hi^F4/80^hi^ TAMs and Cbx4-cKO CD11b^hi^F4/80^hi^ TAMs. (**E**) Western blot analyses of WT CD11b^hi^F4/80^hi^ TAMs and Cbx4-cKO CD11b^hi^F4/80^hi^ TAMs. p-STAT, phosphorylated STAT. (**F**) Heatmap of scaled type I IFN–related gene expression and innate immune–related gene expression between control and sgCbx4 Hepa1-6 tumor cells. (**G**) Heatmap of scaled type I IFN–related gene expression and innate immune-related gene expression in the Tongji cohort HCC data, which was grouped by CBX4 expression levels. (**H**) Heatmap of scaled type I IFN–related gene expression and innate immune-related gene expression in Cbx4-cKO TAMs with and without siRNA inhibition of the indicated dsDNA and dsRNA sensors. (**I**–**K**) Hepa1-6 tumor volumes (**I**), tumor growth curves (**J**), and tumor weights (**K**) were assessed for the following groups: control, sgCbx4, sgCbx4 + sgRig-i, sgCbx4 + sgcGAS, sgCbx4 + sgMavs, sgCbx4 + sgSting1 (*n* = 6). Data represent the mean ± SEM. *P < 0.05, **P < 0.01, ***P < 0.001, and ****P < 0.0001, by 1-way ANOVA (**K**) and 2-way ANOVA with Tukey’s multiple-comparison test (**J**).

T cells and NK cells are not autonomous in their effector functions. Targeting the reactivation of T cells and NK cells requires coordinated cell-cell interactions and communication, including the secretion of chemokines and activation of IFN-related pathways and antigen presentation pathways (6, 33–35). Given that Cbx4 deficiency in both tumor cells and macrophages triggered CD8^+^ T cell– and NK cell–mediated antitumor immunity, we utilized the CellPhoneDB to infer interactions between tumor cells and macrophages and T cells and NK cells, according to scRNA-seq data (Figure 5A). Notably, we observed interactions involving inflammatory cytokines such as TNF-α, IFN-β, IFN-γ, IL-1 family members such as IL-1b, IL-15, and IL-18 (essential for T cell and NK cell survival and effector cell differentiation) (35–37), chemokines such as Cxcl9 and Cxcl10 (essential for T and NK cell recruitment) (15, 29, 30), and the HLA-E/NKG2D axis (essential for activating the cytotoxic function of T cells and NK cells) (38, 39) between Cbx4-deficient tumor cells and macrophages and T cells and NK cells were markedly increased (Figure 5A). Subsequently, we conducted Kyoto Encyclopedia of Genes and Genomes (KEGG) analysis and GSEA to query the hallmark pathways database on Cbx4-deficient tumor cells and macrophages compared with controls. The analysis revealed that Cbx4 ablation in both tumor cells and macrophages led to the upregulation of multiple immune-related pathways, such as type I IFN response pathways, retinoic acid-inducible gene I (RIG-I)–like receptor signaling and cytosolic DNA–sensing pathways, antigen processing and presentation pathways, and others (Figure 5, B and C, and Supplemental Figure 13A). Consistently, Cbx4 deficiency in tumor cells and macrophages led to increased mRNA and protein levels of RIG-I, MAVS, cGAS and phosphorylated IRF3, IRF7, STAT1, and NF-κB (Figure 5, D–F). Clinically, DEGs in tumor tissues from patients with HCC (Tongji cohort), melanoma, or colorectal cancer additionally revealed that the expression of CBX4 has a negative correlation for genes involved in antigen presentation (MHC I related), innate immunity, and T cell activation (Figure 5G and Supplemental Figure 13, B and C). Consistently, multiplex cytokine assays revealed broad upregulation of inflammatory cytokines in Cbx4-deficient tumor cells and macrophages from tumor-bearing mice (Supplemental Figure 13, D and E).

We next assessed the role of the innate immune receptor pathway in Cbx4 ablation–induced IFN activation by knocking down different dsDNA and dsRNA sensors. Interestingly, loss of Cyclic GMP-AMP synthase (cGAS) only modestly declined Cbx4 ablation–induced IFN signaling in macrophages, suggesting that the cGAS/Sting1 pathway did not appear to be a critical mediator of Cbx4 ablation–induced IFN signaling (Figure 5H). In contrast, loss of RIG-I completely reversed Cbx4 ablation–induced interferon-stimulated gene (ISG) and inflammatory gene expression, indicating that RIG-I was a key mediator (Figure 5H). To determine whether Cbx4 mainly influences tumor progression and immune microenvironment remodeling via the Rig-i–like receptor signaling pathway, we generated stable Cbx4/RIG-I double-ablation, Cbx4/cGAS double-ablation, Cbx4/Mavs double-ablation, and Cbx4/Sting1 double-ablation Hepa1-6 cell lines (Figure 5I). The tumor-bearing mouse model data and multiplex cytokine assays showed that Rig-i ablation attenuated the tumor-suppressive and immune-modulatory effects associated with Cbx4 deficiency (Figure 5, I–K, and Supplemental Figure 13F). Collectively, these findings suggest that the deficiency of Cbx4 in tumor cells and macrophages mediates a shared mechanism, which is to remodel the TME through the RIG-I receptor signaling pathway.

### CBX4 represses H3K9me3- and H3K27me3-marked endogenous retrotransposons.

**Figure 6:**
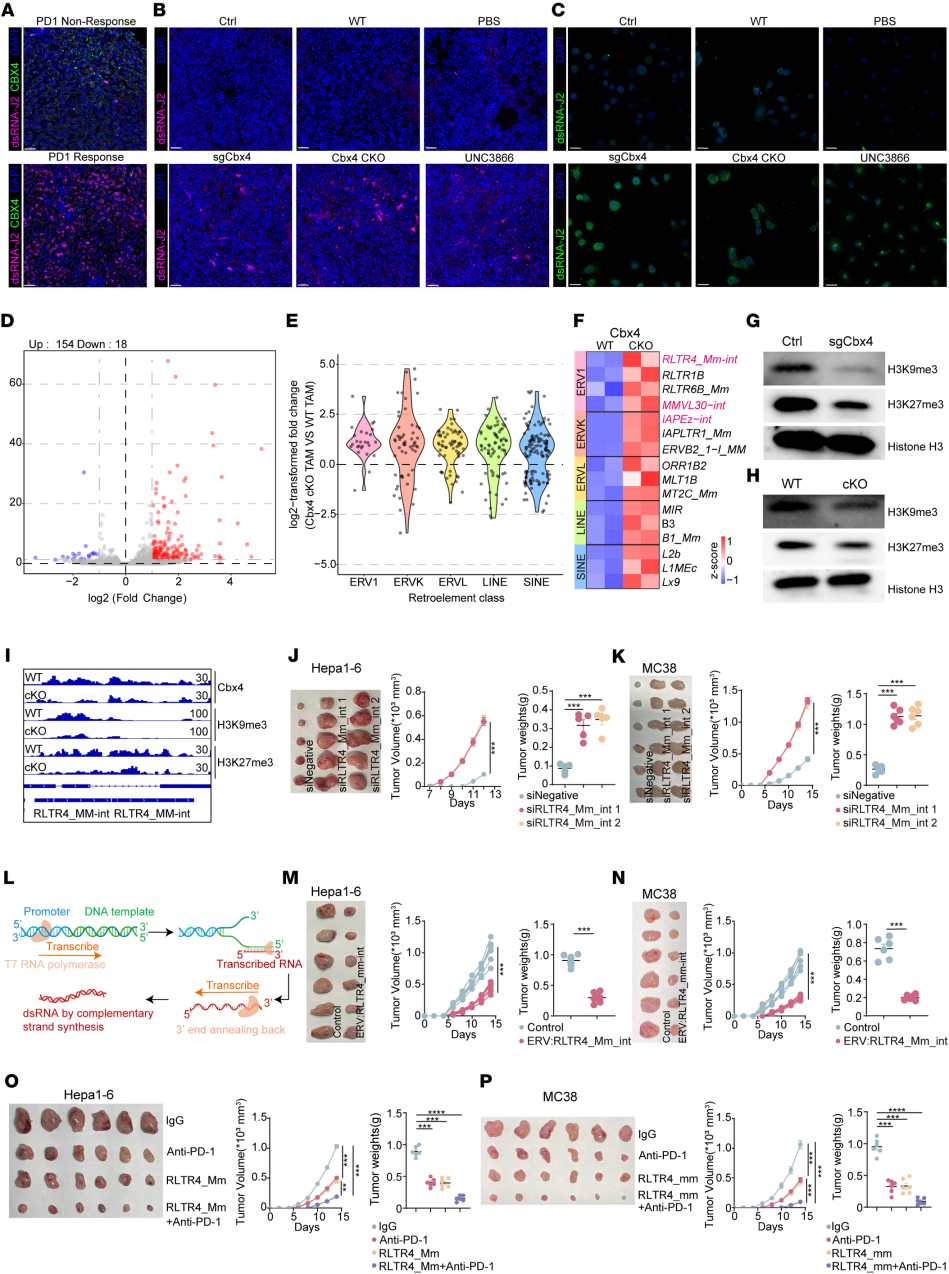
Deletion of CBX4 derepresses endogenous retroelements. (**A**) Representative images from clinical HCC tissues with responsive or nonresponsive to anti–PD-1 treatment. Scale bars: 50 μm. (**B**) Representative images from Hepa1-6 tumor tissues. Scale bars: 50 μm. (**C**) Representative images from control and sgCbx4 Hepa1-6 tumor cells, WT and Cbx4-cKO TAMs, treated with or without 1 μM UNC3866 BMDMs. Scale bars: 20 μm. (**D**) Volcano plot showing retroelement loci with increased expression (red) and decreased expression (blue) in Cbx4-cKO CD11b^hi^F4/80^hi^ TAMs versus WT CD11b^hi^F4/80^hi^ TAMs, combining both forward and reverse strands. (**E**) Violin plots showing differentially expressed retroelement classes comparing Cbx4-cKO CD11b^hi^F4/80^hi^ TAMs with WT CD11b^hi^F4/80^hi^ TAMs. (**F**) Heatmap of scaled retroelement loci expression in differential retroelement classes comparing Cbx4-cKO CD11b^hi^F4/80^hi^ TAMs with WT CD11b^hi^F4/80^hi^ TAMs. (**G** and **H**) Western blots showing the expression of H3K9me3 and H3K27me3 in control and sgCbx4 Hepa1-6 tumor cells (**G**) and WT and Cbx4-cKO CD11b^hi^F4/80^hi^ TAMs (**H**). (**I**) Integrative Genomic Viewer (IGV) screenshots of Cbx4, H3K9me3, and H3K27me3 CUT&Tag signals from WT and Cbx4-cKO CD11b^hi^F4/80^hi^ TAMs. (**J** and **K**) sgCbx4-Hepa1-6 and MC38 tumor volumes, tumor growth curves, and tumor weights were assessed for siNegative, si RLTR4_Mm_int1, and si RLTR4_Mm_int2 groups (*n* = 5). (**L**) Schematic of dsRNA production. (**M** and **N**) Hepa1-6 and MC38 tumor volumes, tumor growth curves, and tumor weights were assessed for control and ERV: RLTR4_Mm_int (*n* = 6) groups. (**O** and **P**) Hepa1-6 and MC38 tumor volumes, tumor growth curves, and tumor weights were assessed for IgG, anti–PD-1, ERV: RLTR4_Mm_int, and ERV: RLTR4_Mm_int + anti–PD-1 groups (*n* = 6). Data represent the mean ± SEM. Tumor growth curve data were analyzed by 2-way ANOVA with Tukey’s multiple-comparison test (**J**, **K**, and **M**–**P**). Other data were analyzed by 1-way ANOVA (**J**, **K**, **O**, and **P**) and 2-tailed, unpaired Student’s *t* test (**M** and **N**). **P < 0.01, ***P < 0.001, and ****P < 0.0001.

The RIG-I receptor signaling pathway could be activated by endogenous dsRNAs, including mis-spliced transcripts and endogenous retroviruses (ERVs), which are major contributors to cytosolic dsRNAs (40, 41). However, there was no enrichment of spliceosome-associated pathways in Cbx4-deficient tumor cells or macrophages (Supplemental Figure 8A and Figure 5B). We speculate that Cbx4 deficiency activates ERVs and endogenous nucleic acid–sensing pathways. Staining human samples with a dsRNA-specific antibody (J2) revealed higher levels of dsRNAs in anti–PD-1 responders with low CBX4 expression than in nonresponders with high CBX4 levels (Figure 6A). Furthermore, both in vitro and in vivo experiments demonstrated that targeting Cbx4 induced the accumulation of dsRNA in cytoplasm and tumor tissues (Figure 6, B and C). RNA-seq analysis showed increased expression of bidirectional transcripts from retroelements, including long terminal repeat–containing (LTR-containing) ERVs and non-LTR elements, such as RLTR4-Mm-int, a top Cbx4 ablation–induced ERV in TAMs (Figure 6, D–F). Similar results were observed in Hepa1-6 tumor cells (Supplemental Figure 14A).The fundamental epigenetic mechanisms that regulate ERV expression include DNA methylation and histone modification (42). However, the MethylFlash Global DNA Methylation (5-mC) ELISA assay revealed that Cbx4 ablation in macrophages and tumor cells did not detectably alter global DNA methylation levels, indicating that the activation of ERV expression caused by Cbx4 ablation may have been induced by histone methylation modification (Supplemental Figure 14, B and C). Retrotransposon silencing is primarily controlled by repressive H3K9me3 and H3K27me3 heterochromatin (43–45). Recent studies showed that Cbx4, part of the PRC1 complex, binds to histone H3 trimethylated at Lys-9 (H3K9me3) and H3 trimethylated at Lys-27 (H3K27me3) (46), indicating that Cbx4 may act via chromatin remodeling and modification of histones to regulate ERV expression. Next, we demonstrated that Cbx4 deficiency in TAMs and tumor cells decreased H3K9me3 levels and H3K27me3 levels (Figure 6, G and H). Loss of Cbx4 led to decreased binding of the H3K9me3 and H3K27me3 repressive marks at the RLTR4-MM-int loci (Figure 6I and Supplemental Figure 14E).

Knockdown of RLTR4-Mm-int, a top Cbx4 loss–induced ERV, in macrophages decreased type I IFN responses without affecting the expression of other ERVs (Supplemental Figure 14, D and F). Furthermore, to determine whether Cbx4 mainly influences tumor progression and immune microenvironment remodeling through the activation of RLTR4-Mm-int, we generated stable Cbx4/RLTR4-Mm-int double-knockdown Hepa1-6/MC38 cell lines (Figure 6, J and K). Our findings suggest that RLTR4-Mm-int knockdown restored the tumor-suppressive and immune-modulatory effects associated with Cbx4 deficiency (Figure 6, J and K). To better ascertain the potential therapeutic value of RLTR4-Mm-int, we designed the ERV: RLTR4-Mm-int for in vivo treatment (Figure 6L). Intratumoral injection of the ERV: RLTR4_Mm_int significantly inhibited Hep1-6 and MC38 tumor growth (Figure 6, M and N). Flow cytometry showed the ERV: RLTR4_Mm_int increased the proportion of CD8^+^ T cells, CD8^+^ Tef cells (IFN-γ^+^TNF-α^+^ and perforin^+^granzyme B^+^), CD8^+^ Tpex cells (PD-1^+^TIM3^–^TOX^+^TCF1^+^), CD8^+^ Tprof cells (Ki67^+^), early-activated CD8^+^ T cells (CD69^+^), and NK and cyto-NK cells (IFN-γ^+^TNF-α^+^ and perforin^+^granzyme B^+^), whereas the proportion of Tex cells (PD-1^+^TIM3^+^ and PD-1^+^LAG3^+^) was reduced (Supplemental Figure 15, A and B). In vitro and in vivo experiments showed that ERV: RLTR4_Mm_int induced the expression and release of inflammatory factors, thereby reshaping the immunosuppressive TME (Supplemental Figure 15, C–F). Then, we used mono or combinatory therapies of ERV: RLTR4_Mm_int and PD-1 blockade in immune-competent murine HCC and CRC models (Figure 6, O and P). As hypothesized, its combination with ICB further augmented therapeutic potency, yielding robust and durable antitumor responses (Figure 6, O and P).

To explore the potential interactions between CBX4 and known epigenetic regulators of ERV expression, we performed immunoprecipitation coupled with mass spectrometry (IP-MS) using anti-CBX4 antibodies. Our results demonstrated that CBX4 specifically interacted with the epigenetic regulators EZH2 (primarily targets H3K27me3) and SETDB1 (primarily targets H3K9me3) (47, 48), suggesting that CBX4 may suppress ERV expression by forming a multi-protein complex with these factors. In contrast, we did not observe an interaction between CBX4 and EP300 or KDM5B (Supplemental Figure 16, A–C). To assess whether pharmacological modulation of histone methylation could reverse CBX4-mediated immunosuppression, we performed rescue experiments using specific inhibitors targeting the histone marks H3K9me3 and H3K27me3, which are regulated by CBX4. We selected specific inhibitors of SETDB1 (SETDB1-TTD-IN-1) and EZH2 (GSK126) to pharmacologically suppress H3K9me3 and H3K27me3 levels in subsequent rescue experiments. We then established stable tumor cell lines overexpressing Cbx4 and conducted subcutaneous tumor implantation assays. As expected, Cbx4 overexpression markedly reduced the proportions of tumor-infiltrating CD8^+^ T cells and NK cells. Importantly, treatment with the H3K9me3 or H3K27me3 inhibitors effectively disrupted this immunosuppressive microenvironment, restoring immune cell infiltration and inhibiting tumor progression (Supplemental Figure 16, D–M). These data demonstrate that targeting histone methylation can functionally reverse the immune evasion induced by CBX4.

To better enhance the clinical relevance, we also analyzed RNA-seq datasets from pretreatment melanoma tumor samples that were sampled before anti–PD-1 therapy (49). We found that CBX4 expression negatively correlated with the ERVs, such as ERVmap_2550, ERVmap_2192, and ERVmap_705, etc., which were expressed at significantly higher levels in patients with complete responses (CRs) to anti–PD-1 treatment than in patients with progressive disease (Supplemental Figure 17, A–C). Overall, we demonstrated that the deficiency of Cbx4 in tumor cells and macrophages mediated a shared mechanism, which is to remodel the TME through derepression of the H3K9me3- and H3K27me3-marked endogenous retrotransposons RLTR4-MM-int. ERV: RLTR4_Mm_int may act as a therapeutic target for enhancing antitumor immunity and cancer immunotherapy.

### Targeting CBX4 induces antitumor immunity and improves the in vivo antitumor effects of PD-1 blockade.

**Figure 7:**
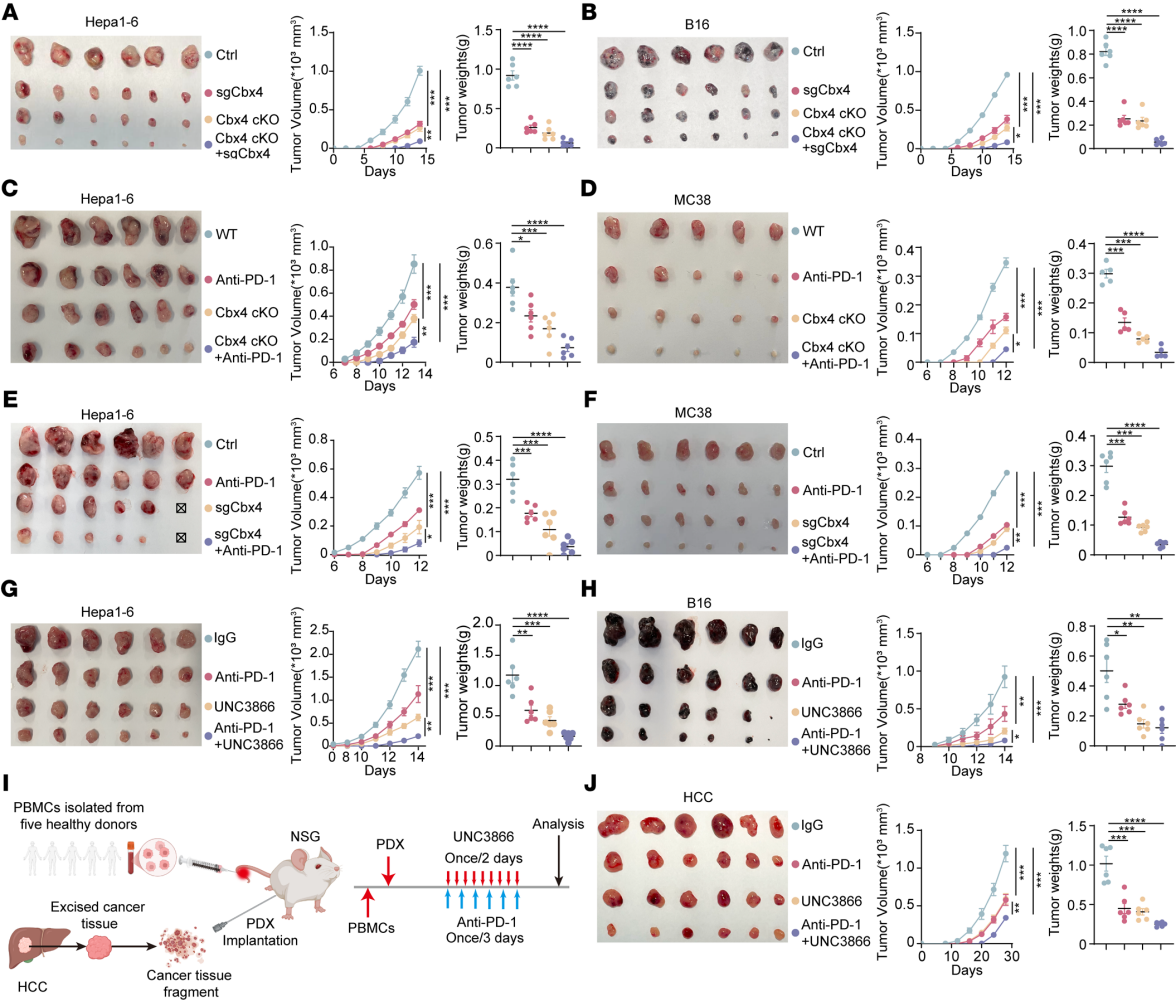
Targeting CBX4 strengthens the anti–PD-1 antitumor response. (**A**) Hepa1-6 tumor volumes, tumor growth curves, and tumor weights were assessed for WT (Ctrl), sgCbx4, Cbx4-cKO, sgCbx4 + Cbx4-cKO groups (*n* = 6). (**B**) B16 tumor volumes, tumor growth curves, and tumor weights were assessed for WT (Ctrl), sgCbx4, Cbx4-cKO, and sgCbx4 + Cbx4-cKO groups (*n* = 6). (**C**) Hepa1-6 tumor volume, tumor growth curve and tumor weight were assessed for the following groups: WT, WT + anti-PD1, Cbx4 cKO, anti-PD1 + Cbx4 cKO. (*n* = 6). (**D**) MC38 tumor volumes, tumor growth curves, and tumor weights were assessed for WT, WT + anti–PD-1, Cbx4-cKO, and anti–PD-1 + Cbx4-cKO groups (*n* = 5). (**E**) Hepa1-6 tumor volumes, tumor growth curves, and tumor weights were assessed for control, anti–PD-1 + control, sgCbx4, and anti–PD-1 + sgCbx4 groups (*n* = 6). (**F**) MC38 tumor volumes, tumor growth curves, and tumor weights were assessed for control, anti–PD-1 + control, sgCbx4, and anti–PD-1 + sgCbx4 groups (*n* = 6). (**G**) Hepa1-6 tumor volumes, tumor growth curves, and tumor weights were assessed for IgG, anti–PD-1, UNC3866, and anti–PD-1 + UNC3866 groups (*n* = 6). (**H**) B16 tumor volumed, tumor growth curved, and tumor weights were assessed for IgG, anti–PD-1, UNC3866, and anti–PD-1 + UNC3866 groups (*n* = 6). (**I**) Schematic overview of PDX-engrafted humanized mice. (**J**) HCC tumor volumes, tumor growth curves, and tumor weights were assessed for IgG, anti–PD-1, UNC3866, and anti–PD-1 + UNC3866 groups (*n* = 6). Data represent the mean ± SEM. Tumor growth curves data were analyzed by 2-way ANOVA with Tukey’s multiple-comparison test (**A**–**J**). Other data were analyzed by 1-way ANOVA (**A**–**J**). *P < 0.05, **P < 0.01, ***P < 0.001, and ****P < 0.0001.

To better understand how concurrent Cbx4 loss in both compartments would affect tumor progression and immune responses, we compared the effects of Cbx4 deficiency in tumor cells and macrophages separately and combined Cbx4 deficiency using Hepa1-6 and B16 tumor–bearing mouse models. Combined Cbx4 deficiency remarkably inhibited tumor growth and induced the production of more effector T cells and NK cells (Figure 7, A and B, and Supplemental Figure 18, A–F). Next, using Hepa1-6 and MC38 tumor–bearing mouse models, we demonstrated that CBX4 ablation in both macrophages (Figure 7, C and D) and tumor cells (Figure 7, E and F) could boost the efficacy of anti–PD-1 immunotherapy. Consistently, we observed that the combination of anti–PD-1 and CBX4 ablation in macrophages did not change the proportion of total tumor-associated macrophages (Supplemental Figure 19A), but increased the expression of MHC class I molecules (Supplemental Figure 19B). However, the proportion of CD86^+^ TAMs was substantially higher in the combination group, alongside a marked decrease in immunosuppressive CD206^+^ macrophages (Supplemental Figure 19, C and D). Importantly, the proportions of cytotoxic CD8^+^ T cells, NK cells, and cytotoxic TNF-α^+^IFN-γ^+^CD8^+^ T cells were significantly increased (Supplemental Figure 19, E–G), with the combination therapy further augmenting this effect. To assess the therapeutic value of targeting CBX4, Hepa1-6 and B16 tumor–bearing mice were treated with either the CBX4 inhibitor UNC3866 or anti–PD-1 antibodies, or their combination, and tumor growth was monitored. We noticed that the combination of CBX4 inhibition and anti–PD-1 antibodies substantially slowed tumor growth in both Hepa1-6 and B16 tumor–bearing mouse models (Figure 7, G and H). Consistently, we observed that the proportion of total TAMs did not change significantly (Supplemental Figure 19H). However, the proportion of CD86^+^ TAMs was substantially higher in the combination group, along with a marked decrease in immunosuppressive CD206^+^ macrophages (Supplemental Figure 19, J and K). Meanwhile, the expression of MHC class I molecules with antigen presentation capability was markedly increased (Supplemental Figure 19I). Importantly, the proportions of CD8^+^ T cells, NK cells, and cytotoxic TNF-α^+^IFN-γ^+^CD8^+^ T cells were significantly increased after UNC3866 monotherapy, further augmented by combination therapy (Supplemental Figure 19, L–N). UNC3866 only mildly inhibited tumor growth in Rag^–/–^ γc^–/–^ mice. However, in immunocompetent C57BL/6J mice, UNC3866 significantly suppressed tumor growth and increased the proportions of CD8^+^ T cells and NK cells (Supplemental Figure 19, Q and R). These results showed that immune-mediated, UNC3866-limited tumor growth was more dependent on the functions of immune cells. To better simulate clinical responses to immunotherapy, we established patient-derived xenograft (PDX) models in humanized NSG mice using freshly resected HCC specimens from individuals with distinct CBX4 expression and immunotherapeutic outcomes. Compared with the control, UNC3866 monotherapy significantly inhibited tumor growth and reduced the tumor burden. As hypothesized, the combination of UNC3866 with ICB further augmented therapeutic potency, yielding robust and durable antitumor responses (Figure 7, I and J). Collectively, these findings indicated that CBX4 is a promising therapeutic target that sensitizes tumors to ICB, offering a compelling combinatorial strategy for enhancing antitumor immunity. These results highlight the use of epigenetic-immune cotargeting as a promising and clinically actionable strategy to enhance cancer immunotherapy.

## Discussion

Epigenetic regulators have recently been implicated in immune escape and immunotherapy resistance, making them attractive targets for epigenetic targeting or combination therapy with immunotherapy (43, 50–52). To systematically identify intrinsic immune evasion pathways in tumor cells, we performed in vivo CRISPR/Cas9 screening using an epigenetic library in Hepa1-6 and MC38 transplantable mouse tumor models treated with ICB. We identified CBX4 as an epigenetic target involved in immune evasion and resistance to immunotherapy, which accumulated in nonresponders to anti–PD-1 treatment in both tumor cells and in subpopulations of immunosuppressive TAMs. Targeting CBX4 derepressed H3K9me3- and H3K27me3-marked endogenous retroelements such as RLTR4-MM-int and triggered the cytosolic RNA–sensing pathway and subsequent activation of the type I IFN response, leading to a robustly inflamed TME and ultimately sensitizing ICB-based immunotherapy.

CBX4, as a component of the canonical polycomb-repressive complex 1 (PRC1), contains a chromodomain that recognizes H3K27me3 and permits binding of PRC1 to these specific chromatin regions, leading to chromatin compaction and target gene repression (53). CBX4 represses the transcript and protein levels of p16/INK4a during embryogenesis and prevents human epidermal stem cell senescence (54). CBX4 is also a special chromobox protein because it is a SUMO E3 ligase, and it can act as both oncogene and tumor suppressor depending on the cell type as well as its interacting partners (54). CBX4 can both promote and suppress tumor progression depending on its interacting partners. For instance, CBX4 inhibits tumorigenesis under the Kras^G12D^ background via repression of P15, P16, and other apoptosis-related genes in immunodeficient mice (55). CBX4 recruits histone deacetylase 3 (HDAC3) to the Runx2 promoter and suppresses Runx2 expression, thereby inhibiting the metastasis of colorectal carcinoma (56), whereas CBX4 promotes metastasis in osteosarcoma through induction of Runx2 via the recruitment of lysine acetyltransferase 2A (KAT2A) to the Runx2 promoter (57), and CBX4 is significantly correlated with proliferation and angiogenesis of HCC through enhancement of HIF-1α sumoylations in vitro and in immunodeficient mice (58). All of these findings indicate that the function of CBX4 is dependent on its partners and microenvironments. Utilizing both syngeneic immunodeficient and immunocompetent mice in concurrent experiments, we demonstrated that CBX4 suppressed multiple immune-related pathways, such as type I IFN responses, RIG-I–like receptor signaling, and cytosolic DNA sensing during ICB therapy. Unlike in vitro or in immunodeficient mice, CBX4 promotes tumor cell survival against antitumor immunity in immunocompetent mice, suggesting that CBX4 mainly functions as a repressor of multiple immune-related pathways under immune pressure and serves as a target for ICB therapy.

Interestingly, we determined that CBX4 deficiency in tumor cells and macrophages induced immune-related gene expression through reactivation of ERVs. In addition to repressing coding genes, CBX4 also repressed ERV expression by binding to endogenous retrotransposon regions. Our data reveal that loss of CBX4 derepressed endogenous retrotransposons RLTR4-MM-int, resulting in a dramatic increase of cytosolic dsRNA and, consequently, triggering IFN signaling. We also established a negative correlation between CBX4 and ERVmap expression such as ERVmap_2550, ERVmap_2192, and ERVmap_705, which was markedly higher in patients with a complete response to anti–PD-1 treatment compared with those with progressive disease. Although, to our knowledge, there are no published data showing that CBX4 represses ERV expression, CBX4 is considered to recognize H3K27me3 and H3K9me3, which maintains ERVs in a silent state (58, 59). Recent work suggests that the inhibitors of EZH2, the predominant catalytic subunit of PRC2, diminishes H3K27me3 at ERVs and induces dsRNAs and ISGs, along with increased activation of tumor-infiltrating CD8^+^ T cells, augmenting ICB in murine and human prostate cancers (60). H3K9 methyltransferase SETDB1 promotes H3K9me3 to repress endogenous retroelements, enhancing tumor immunogenicity (24, 43). And our Cut&Tag data showed that CBX4 bound to the same region with H3K27me3 and H3K9me3, suggesting that CBX4 could bind to H3K27me3 and H3K9me3 to repress H3K9me3- and H3K27me3-marked endogenous retrotransposons.

ERVs are retrotransposons that spread throughout the genome and can be regulated by promoters and enhancer elements, attract DNA and histone modifying complexes (61, 62). Because of the sequence and structural analogies with exogenous viruses, ERVs can trigger robust antiviral innate immunity via cytosolic sensors (e.g., RIG-I, MDA5), enhancing cytokine and chemokine secretion. Moreover, ERVs that encode peptides have been identified as alternative antigens in many tumors (63). These ERV-derived antigens can be presented by MHC I for specific T cell activation and trigger adaptive immune responses. So, future studies should be warranted to explore whether the induced ERVs contribute to the gross antigen pools of *CBX4*-deficient tumor to induce antigen-specific T cell responses. Indeed, ERVs affect the TME and have applications in ICB augmentation (64). Here, we demonstrated that targeting CBX4 induced ERVs and cytosolic dsRNA, thereby triggering IFN and RIG-I signaling, suggesting that ERVs serve as promising therapeutic targets during ICB therapy by reshaping the TME.

The TME is a complex interplay of diverse cell types, with regulation of their interactions managed by mechanisms including direct cellular contacts and paracrine signaling (65, 66). Recent advances in tumor immunotherapy aimed at rejuvenating tumor-infiltrating T cells and NK cells have demonstrated clinical benefits across various types of cancer (67–69). Targeting the reactivation of CD8^+^ T cells and NK cells requires coordinated cell-cell interactions and communication, which depend on innate immune responses (10, 18, 19). Tumor cells and macrophages within the TME promote immune surveillance via dsRNA/IFN and STING/IFN-β signaling, among others (18, 19, 33). Here, our data showed that elevated ERV expression augments innate immunity in the immune-compromised TME and is crucial for the efficacy of ICB. We found that CBX4 ablation in tumor cells and macrophages elevated the expression of multiple chemokines, including CXCL9 and CXCL10, and increased the levels of molecules responsible for antigen processing and presentation by activating the dsRNA/IFN signaling pathway, which augments CD8^+^ T cell– and NK cell–mediated antitumor immune responses. We also found that immunotherapeutic treatment blocking the PD-1/PD-L1 axis had a striking synergistic effect when targeting CBX4 in tumors and macrophages, indicating that CBX4 loss could sensitize the refractory tumor to anti–PD-1 blockade treatment. These data show that CBX4 serves as a retrotransposon regulator and that CBX4-mediated ERV silencing functions as an intrinsic checkpoint for antitumor immunity. In the future, more work is needed to explore the integration of ERV silencing and tumor epigenetic regulators.

Our findings suggest that targeting CBX4 induced ERV expression and RIG-I signal activation, thereby enhancing ICB therapy. Moreover, CBX4 may serve as a biomarker for stratifying patients with cancer on ICB therapies such as anti–PD-1 or anti–PD-L1 treatment and for predicting immunotherapeutic efficacy and clinical prognosis.

## Methods

### Sex as a biological variable.

Our study examined samples from both male and female donors. Experiments were conducted on female and male mice in separate studies. Sex was not considered as a biological variable. While our study focused on male mice, the findings may be relevant for both sexes.

Detailed methods and materials, including experimental design, protocols, data analysis, all reagents, and key resources are presented in the Supplemental Methods.

### Statistics.

Statistical analysis is specified in the figure legends. A *P* value of less than 0.05 was considered significant. Data are presented as the mean ± SEM. Comparisons among multiple groups were analyzed by 1-way ANOVA, while comparisons between 2 groups were performed using Welch’s 2-tailed *t* tests. Survival curves were generated using the Kaplan-Meier method and compared with the log-rank test. Correlations between 2 continuous variables were assessed using Pearson’s correlation analysis. Longitudinal tumor growth data were evaluated by repeated-measure 2-way ANOVA.

### Study approval.

Animal experimental protocols were approved by the IACUC of the Tongji Hospital, Huazhong University of Science and Technology (ethics approval no. TJH-202202001). The Clinical study received approval from the Ethics Committee of Tongji Hospital (HUST, Wuhan, China), and informed consent was obtained from every patient (approval no. TJ-IRB202412182).

### Data availability.

Raw data files from bulk RNA-seq, Cut&Tag-seq, and scRNA-seq experiments were deposited to the Genome Sequence Archive (GSA) database (https://ngdc.cncb.ac.cn/gsub/) affiliated with the National Genomics Data Center (https://ngdc.cncb.ac.cn/) (identifier accession codes: CRA022150, CRA022114, and CRA022205). All individual values represented in graphs are provided in the Supporting Data Values file. The data that support the findings of this study are available from the corresponding author upon reasonable request.

## Author contributions

ZM, WJ, and XZ share the co–first author credit. The order of the first authors reflects the contributions of the individual authors. PL, ZM, WJ, and XZ designed the experiment and interpreted the data. ZM and XZ performed most of the experiments. JL, QL, GS, and NY assisted in some experiments. WJ and RC conducted the data analyses. PL and ZM provided the key materials and the overall guidance. PL and ZM wrote the manuscript. PL, ZM, and ZC provided funding for the project.

## Conflict of interest

The authors have declared that no conflict of interest exists. 

Funding support

National Natural Science Foundation of China (NSFC) (825B2050, 82271807, 82471810, and 82371794).

Key Science and Technology Project of Hubei Provincial Health Commission (WJ2025Z020).

Nonprofit Central Research Institute Fund of China (2023-PT320-07).

Fundamental Research Funds for the Central Universities (YCJJ20251102).

## Supplementary Material

Supplemental data

Unedited blot and gel images

Supporting data values

Supplemental table 1

Supplemental table 2

## Version 1 03/31/2026

In-Press Preview

## Version 2 05/15/2026

Electronic publication
