# Supplementary material for: In vivo CRISPR screens identify CBX4 as an epigenetic regulator for cancer immunotherapy
Source: J Clin Invest. 2026 Mar 31;136(10):e200564. doi: 10.1172/JCI200564 (PMC13178658; doi:10.1172/JCI200564)
Supplement: Supplemental data [file jci-136-200564-s265.pdf]

Supplement Figures

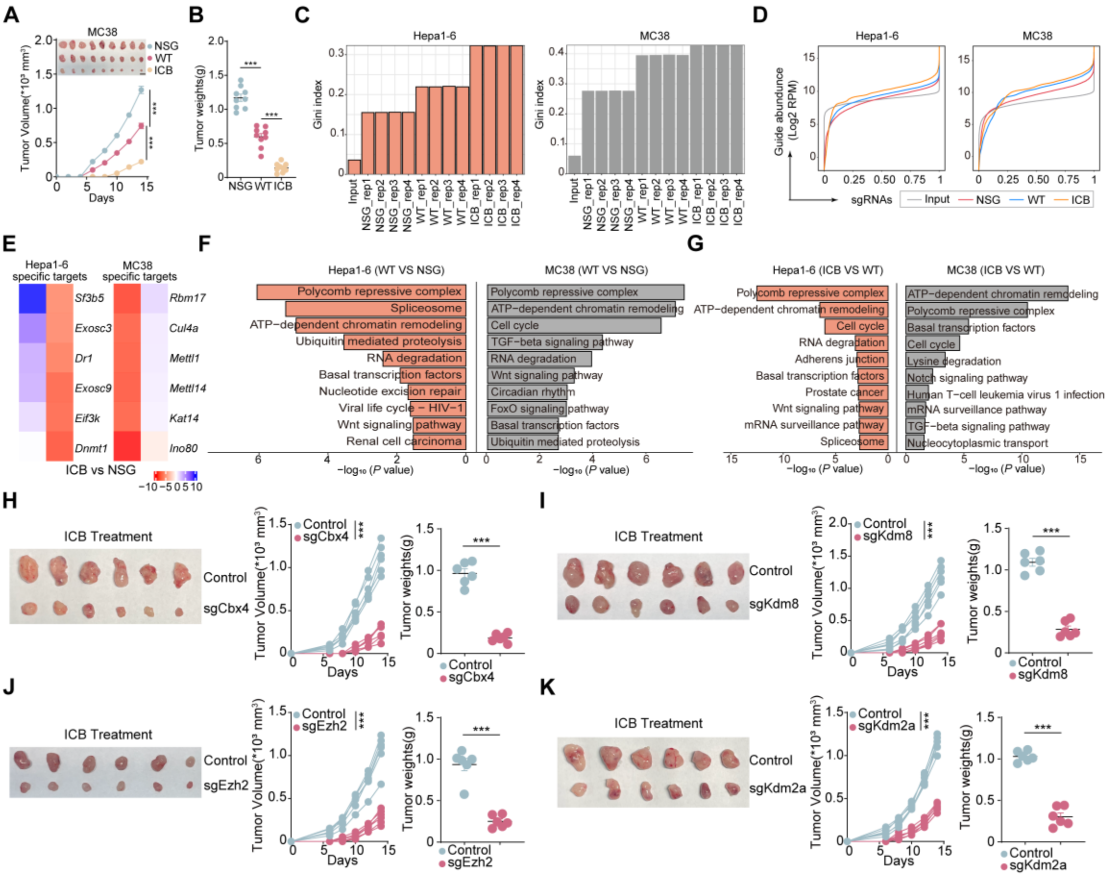

Figure.S1 Quality control assessment of CRISPR–Cas9 screen data, relative to Fig.1.

(A-B) Schematic (up), tumor growth curve (left) and tumor weight (right) for MC38 (n=9) tumors in NSG, C57BL/6J, and C57BL/6J + ICB-treated [anti-PD-1 (200ug at d6, 9, 12)] groups. Scale bars, 1 cm.

(C) The Gini index, which measures read depth evenness within samples.

(D) In vitro and in vivo library recovery.

(E) Depletion (*red*) or enrichment (*blue*) of targeted genes in ICB vs NSG mice grouped by top cell-specific hits. Hepa1-6 specific genes (left) and MC38 specific genes (right).

(F) Core pathway enrichment of deleted genes from the Hepa1-6 and MC38 libraries in WT compared to NSG mice.

(G) Core pathway enrichment of deleted genes from the Hepa1-6 and MC38 libraries in ICB-treated compared to WT mice.

(H-K) Schematic (left), tumor growth curve (left) and tumor weight (right) for Hepa1-6 tumors in Control (treated with ICB) and sgCbx4 (H) /sgKdm8 (I) /sgEzh2 (J) /sgKdm2a (K) (treated with ICB) groups. (n=6).

Data represent mean ± SEM. Tumor growth curves data were analyzed by two-way ANOVA with Tukey's multiple comparisons test (A and H-J). Other data were analyzed by one-way ANOVA (B) and 2-tailed unpaired Student's *t* test (H-J), with the corresponding results expressed as follows: ns, non-significant, **p* < 0.05, ***p* < 0.01, ****p* < 0.001, *****p* < 0.0001.

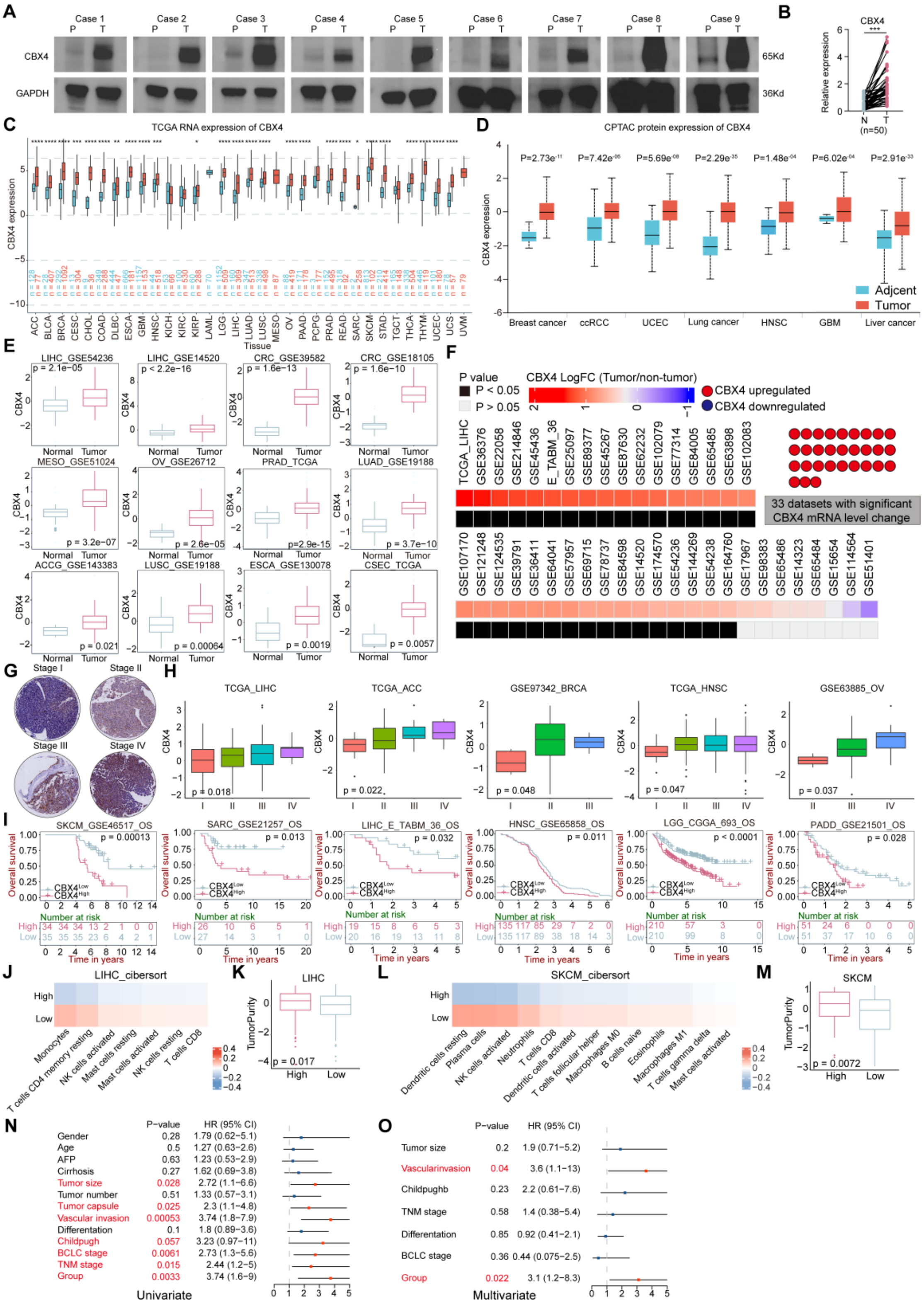

Figure. S2 CBX4 is associated with tumor progression, recurrence, and patient outcome, relative to Fig.1.

(A-B) Representative western blot bands of 50 pairs of CBX4 differentially expressed in HCC and paracancerous tissues from cohort 1 (n=50). Relative CBX4 protein level in HCC tissues (T) by comparing to their counterpart paracancerous tissues (P) after normalizing to GAPDH expression.

(C) The expression level of CBX4 between Pan-cancer tissues and normal tissues from TCGA database.

(D) Protein expression levels of CBX4 in tumor and paratumor tissues across various cancer types based on CPTAC datasets.

(E) The expression level of CBX4 between Pan-cancer tissues and normal tissues from GEO database.

(F) Large-scale data mining was used to compare the expression differences in CBX4 mRNA between HCC tissues and adjacent noncancerous liver tissues.

(G) The expression level of CBX4 with different grades from TCGA and GEO database.

(H) Representative IHC images of CBX4 staining of clinical HCC with different grades.

(I) Kaplan-Meier analyses for overall survival (OS) for CBX4 expressions in pan-cancer GEO datasets.

(J) Comparison of the scores for immune cells estimated by the Cibesort abs algorithm between high and low CBX4 expression in LIHC**.**

(K) Comparison of the scores estimated by the Tumor purity analysis between high and low CBX4 expression in LIHC**.**

(L) Comparison of the scores for immune cells estimated by the Cibesort abs algorithm between high and low CBX4 expression in SKCM**.**

(M) Comparison of the scores estimated by the Tumor purity analysis between high and low CBX4 expression in SKCM**.**

(N and O) Forest plot of the univariate and multivariate Cox proportional hazards model for overall survival. Data are presented as mean values ± 95% confidence interval.

Statistical significance was performed through the utilization of two-tailed paired t test (B), with the corresponding results expressed as follows: $***p < 0.001$.

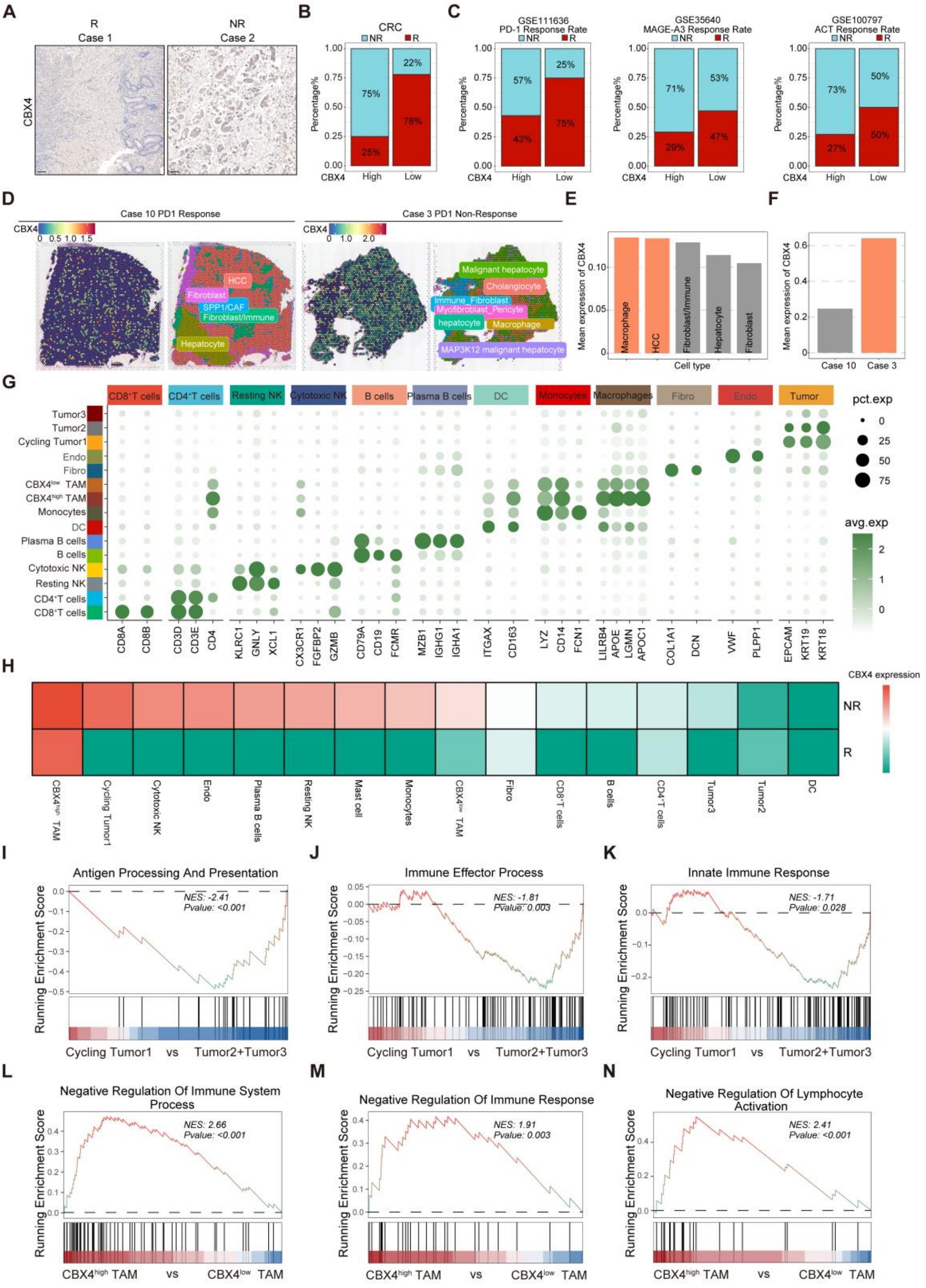

Figure. S3 Single-cell RNA sequencing of RCC reveals CBX4 is highly expressed in both tumor cells and immunosuppressive TAMs subpopulations, relative to Fig.1.

(A) Representative IHC images showing CBX4 staining in clinical CRC samples that are either responsive or non-responsive to ICB treatment.

(B-C) Results based on Tongji CRC cohort, GSE35640, GSE111636 and GSE100797

indicating the relationship between the high and low levels of CBX4 expression and the response rate to immunotherapy.

(D-F) Spatial feature plots of signature score of CBX4 in tissue sections.

(G) Marker gene expression across defined cell clusters. Bubble size is proportional to the percentage of cells expressing a gene and color intensity is proportional to average scaled gene expression.

(H) Heatmap of CBX4 expression in distinct cell populations from groups with responsive and non-responsive to ICB treatment.

(I-K) GSEA plots of three pathways comparing scRNA-seq data from cycling tumor1 population (CBX4^{high}) and Tumor2+ Tumor3(CBX4^{low}) populations.

(L-N) GSEA plots of three pathways comparing scRNA-seq data from CBX4^{high} TAMs population and CBX4^{low} TAMs population.

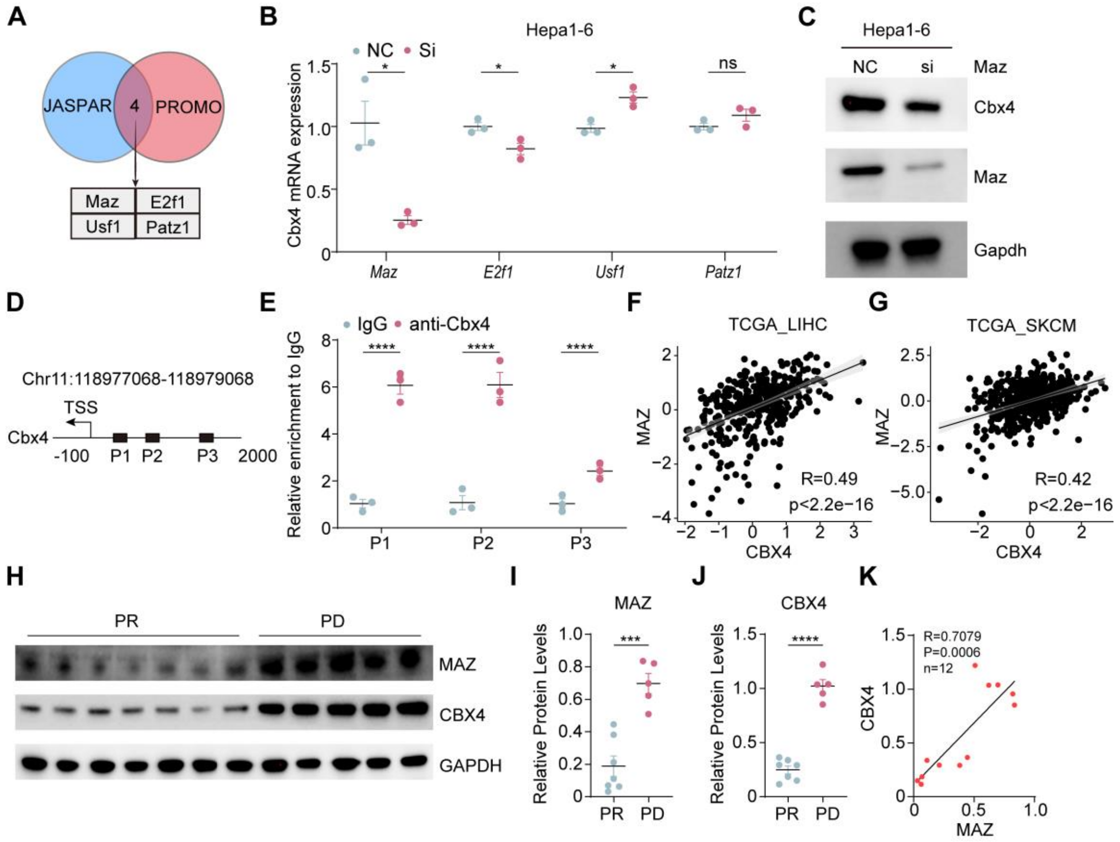

Figure. S4 MAZ upregulates CBX4 expression, relative to Figure.1.

(A) Venn diagram illustrating the intersection of transcription factors predicted to regulate Cbx4 by JASPAR and PROMO databases.

(B) qPCR analysis of Cbx4 mRNA expression in Hepa1-6 tumor cells following siRNA-mediated knockdown of Maz, E2f1, Usf1 and Patz1.

(C) Western blot analysis of Cbx4 and Maz protein expression in Hepa1-6 tumor cells following siRNA-mediated knockdown of Maz.

(D) Predicted Maz binding sites in the Cbx4 promoter according to JASPAR.

(E) Relative enrichment of Maz and IgG (negative control) on predicted binding sites in the Cbx4 promoter assessed by Cut&Tag-qPCR analysis.

(F-G) The correlation between CBX4 and MAZ in public data from hepatocellular carcinoma and Melanoma patients.

(H) Representative Western blot results showing the expression levels of MAZ and CBX4 from clinical HCC tissues with responsive or non-responsive to anti-PD-1 treatment (PR: Partial response; PD: Progressive disease).

(I-J) The expression level of MAZ and CBX4 between PR (n=7) and PD (n=5) tumor tissues.

(K) The correlation between MAZ and CBX4 in tumor tissues (n=12).

Data represent mean ± SEM. Statistical significance was performed through the utilization of 2-tailed unpaired Student's *t* test (B, E and I-J), purity-corrected Spearman test (F-G and K) with the corresponding results expressed as follows: ns, non-significant, $*p < 0.05$, $**p < 0.01$, $***p < 0.001$, $****p < 0.0001$.

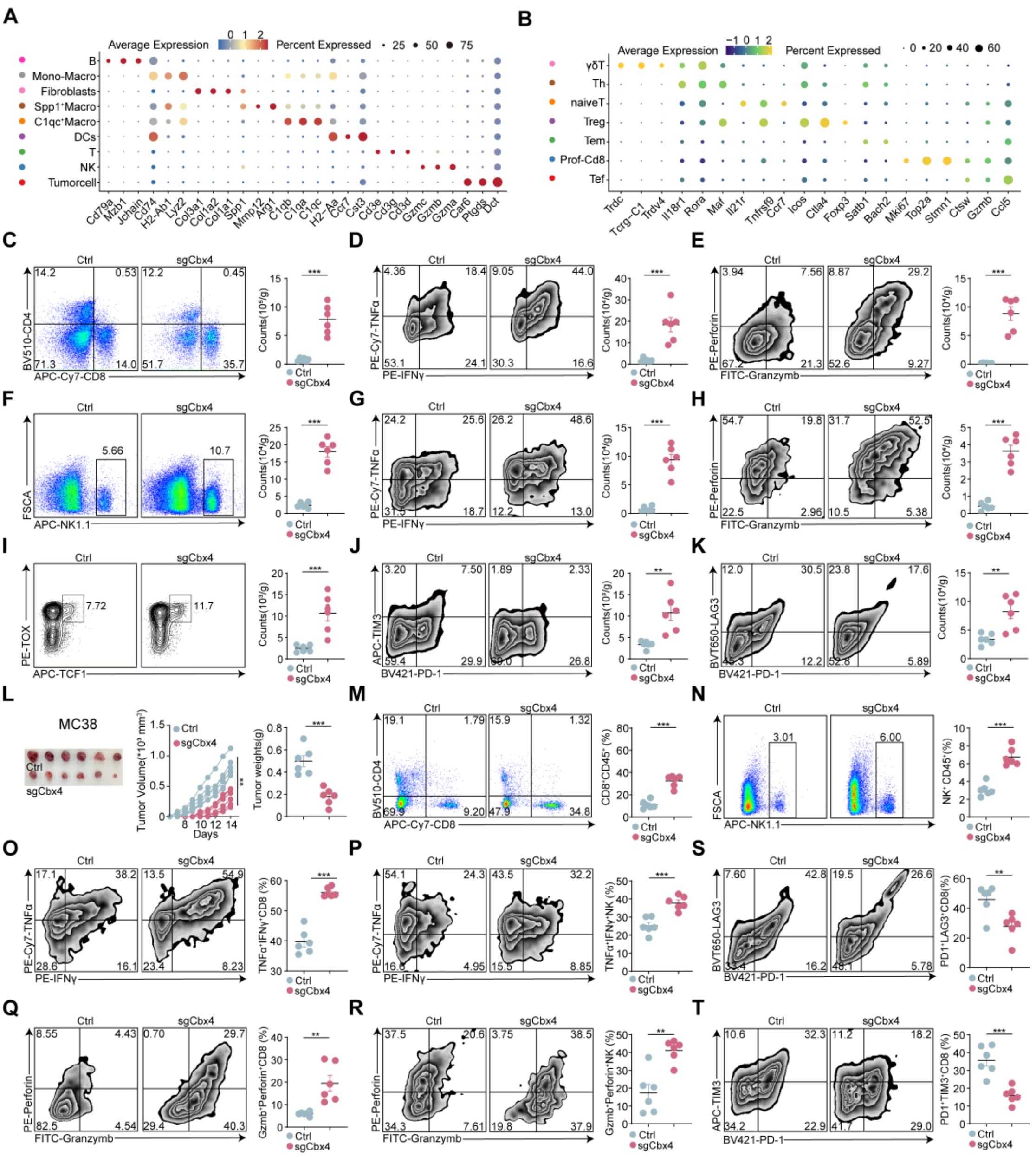

Figure. S5 Loss of CBX4 in tumor cells elicits activation of the tumor inflammatory microenvironment, relative to Figure.2.

(A) Marker gene expression across defined cell clusters. Bubble size is proportional to the percentage of cells expressing a gene and color intensity is proportional to average scaled gene expression.

(B) Marker gene expression across defined cell clusters. Bubble size is proportional to the percentage of cells expressing a gene and color intensity is proportional to average scaled gene expression.

(C) Schematic (left) and counts (right) of $CD8^{+}$T cells in Control and sgCbx4 Hepa1-6 tumors (n=6).

(D-E) Schematic (left) and counts (right) of TNF-α^{+}IFN-γ^{+} $CD8^{+}$ T cells (D) and $Perforin^{+}$ granzyme B^{+} $CD8^{+}$ T cells (E) in Control and sgCbx4 Hepa1-6 tumors o (n=6).

(F) Schematic (left) and counts (right) of NK cells in Control and sgCbx4 Hepa1-6 tumors (n=6).

(G-H) Schematic (left) and counts (right) of TNF-α^{+}IFN-γ^{+} NK cells (G) and Perforin^{+} granzyme B^{+} NK cells (H) in Control and sgCbx4 Hepa1-6 tumors (n=6).

(I-K) Schematic (left) and counts (right) of PD1^{+} TIM3^{-}TOX^{+}TCF1^{+} CD8^{+} T cells (I), PD1^{+} TIM3^{+} CD8^{+} T cells (J) and PD1^{+} LAG3^{+} CD8^{+} T cells (K) in Control and sgCbx4 Hepa1-6 tumors (n=6).

(L) Schematic (left), tumor growth curve (middle) and tumor weight (right) for MC38 (n=6) tumors.

(M-N) Schematic (left) and percentage (right) of CD8^{+}T (M) and NK cells (N) in Control and sgCbx4 MC38 tumors (n=6).

(O-P) Schematic (left) and percentage (right) of TNF-α^{+}IFN-γ^{+} CD8^{+} T cells (O) and Perforin^{+} granzyme B^{+} CD8^{+} T cells (P) in Control and sgCbx4 MC38 tumors (n=6).

(Q-R) Schematic (left) and percentage (right) of TNF-α^{+}IFN-γ^{+} NK cells (Q) and Perforin^{+} granzyme B^{+} NK cells (R) in Control and sgCbx4 MC38 tumors (n=6).

(S-T) Schematic (left) and counts (right) of PD1^{+} LAG3^{+} CD8^{+} T cells (S) and PD1^{+} TIM3^{+} CD8^{+} T cells (T) in Control and sgCbx4 MC38 tumors (n=6).

Data represent mean ± SEM. Tumor growth curves data were analyzed by two-way ANOVA with Tukey's multiple comparisons test (L). Other data were analyzed by 2-tailed unpaired Student's *t* test (C-T), with the corresponding results expressed as follows: ns, non-significant, *$p < 0.05$, **$p < 0.01$, ***$p < 0.001$, ****$p < 0.0001$.

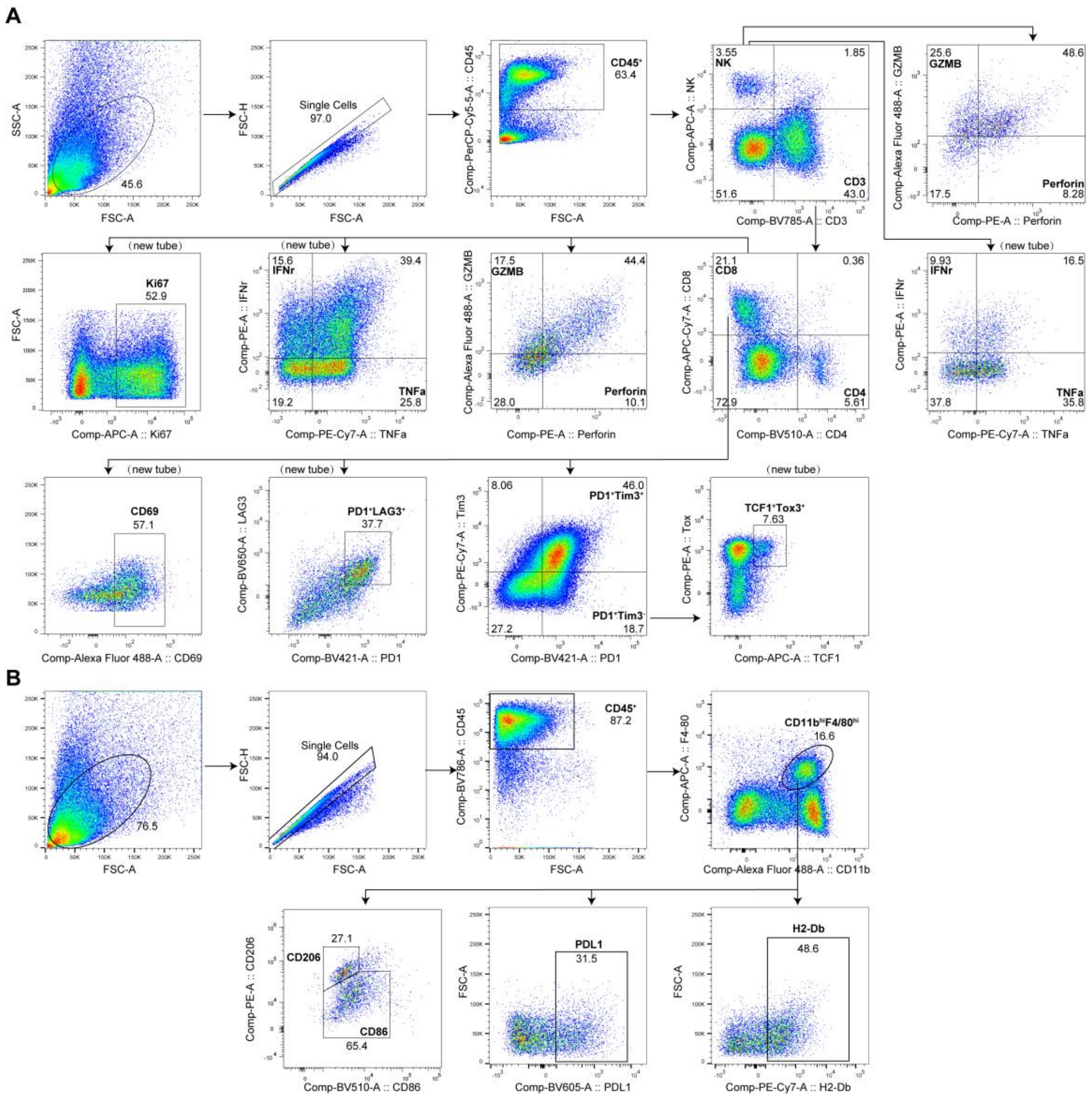

Figure. S6 Gating strategy for flow cytometry to define immune cell subsets, relative to Figure.2 and 3.

(A) Gating strategy for flow cytometry to define lymphoid cell subsets.

(B) Gating strategy for flow cytometry to define myeloid cell subsets.

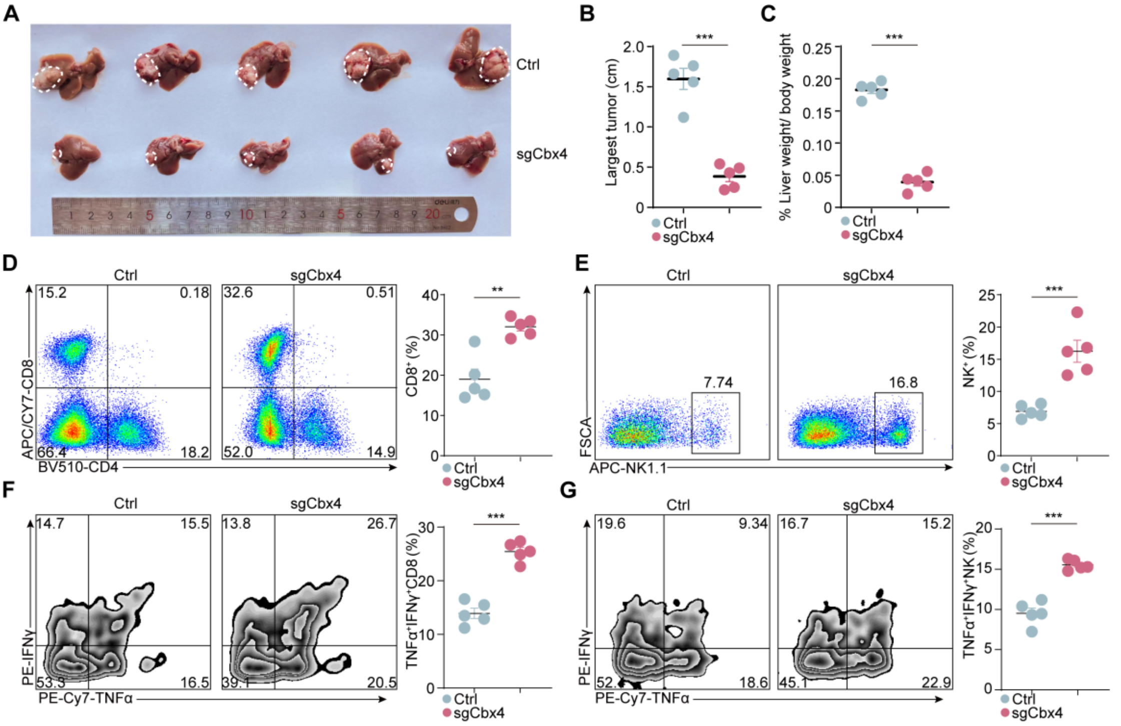

Figure. S7 In the orthotopic liver cancer model, loss of CBX4 in tumor cells augmented the CD8$^+$T cells and NK cells anti-tumor immunity. relative to Figure.2.
(A) The general images of orthotopic tumors derived from C57BL/6J mice in Control and sgCbx4 groups (n=5).
(B) The longest diameters of orthotopic tumors (n=5).
(C) The percentage of liver weight/body weight of orthotopic tumors (n=5).
(D-E) The percentage of CD8$^+$T and NK cells in Control and sgCbx4 Hepa1-6 orthotopic tumors as determined by flow cytometry (n=5).
(F-G) The percentage of TNF-α$^+$IFN-γ$^+$ CD8$^+$ T cells and TNF-α$^+$ IFN-γ$^+$CD8$^+$ NK cells in Control and sgCbx4 Hepa1-6 orthotopic tumors as determined by flow cytometry (n=5).
Data represent mean ± SEM. Statistical significance was performed through the utilization of 2-tailed unpaired Student's *t* test (B-G), with the corresponding results expressed as follows: ns, non-significant, *$p < 0.05$, **$p < 0.01$, ***$p < 0.001$, ****$p < 0.0001$.

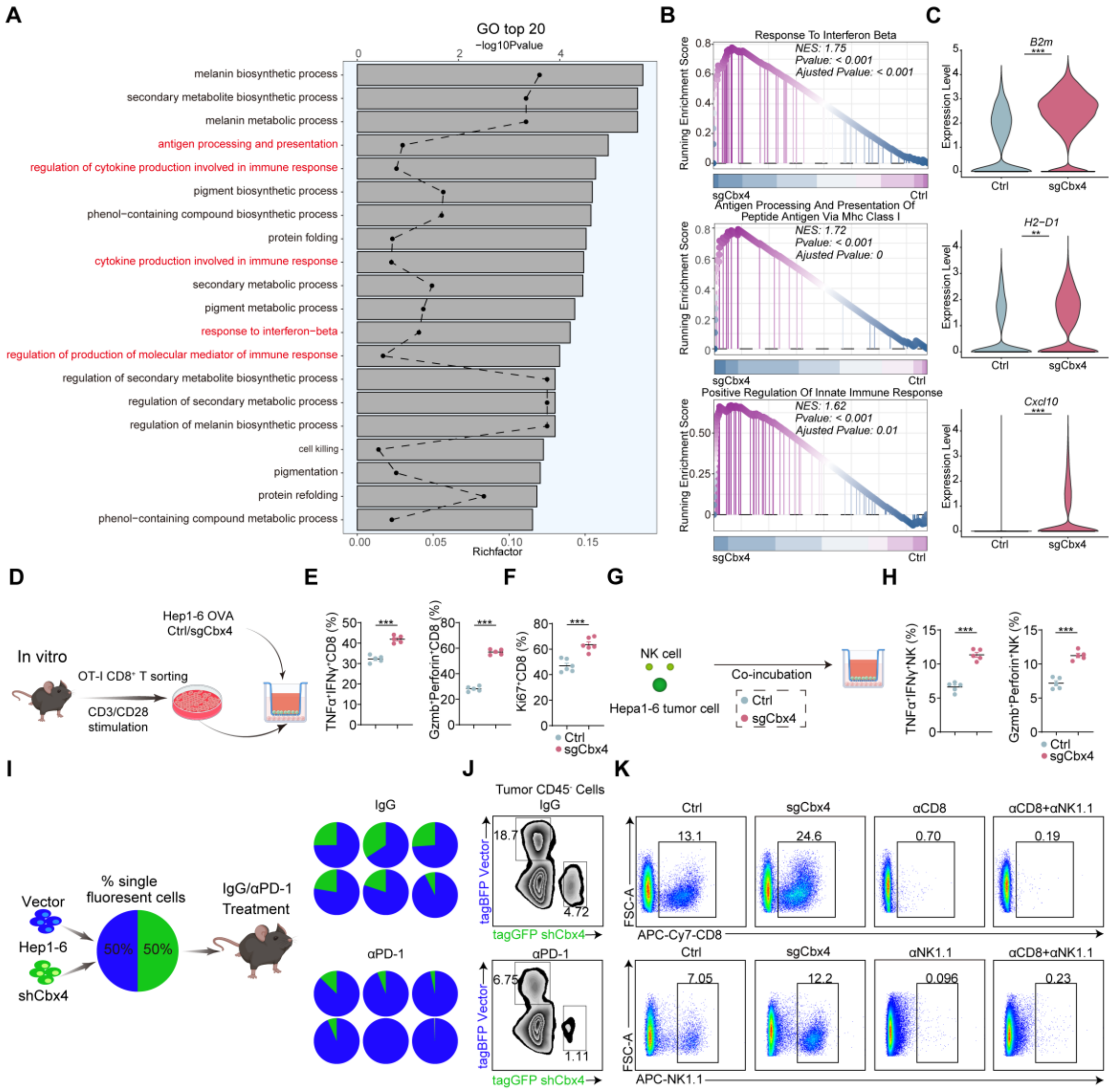

Figure. S8 Loss of CBX4 in tumor cells activates the innate immune response, thereby reshaping the tumor inflammatory microenvironment, relative to Figure.2.
(A) Gene Ontology (GO) pathways data comparing scRNA-seq data from sgCbx4 tumor population and Control tumor population. Shown are selected top 20 upregulated pathways.
(B) GSEA plots of three pathways comparing scRNA-seq data from sgCbx4 tumor population and Control tumor population.
(C) Violin plots showing the expression distributions of *B2m*, *H2-d1* and *Cxcl9* in the tumor population from Control group versus sgCbx4 group.
(D-F) Schematic (D), quantification of TNFα^{+}IFNγ^{+}, Gzmb^{+} Perforin^{+} (E) and Ki67^{+} (F) OVA-specific T cells co-cultured with OVA-expressing Control and sgCbx4 tumor cells.
(G) Schematic design of the *in vitro* co-culture systems of NK cells and Hepa1-6 tumor cells to determine the stimulatory effects of tumor cells (Control or sgCbx4) on the cytotoxic capacity of NK cells.
(H) The percentage of TNF-α^{+} IFN-γ^{+} NK cells and Perforin^{+} granzyme B^{+} NK cells from NK cells co-cultured with Hepa1-6 tumor cells (Control or sgCbx4).

(I) (Left): Schematic of a 50%:50% ratio of Hepa1-6-tagBFP tumor cells (Vehicle) and Hepa1-6-tagGFP (shCbx4) tumor cells in cell mixture before injection for tumor transplantation and ICB treatment. (Right): Statistical analysis of the ratio of tagBFP-Vehicle and tagGFP-Sh-Cbx4 tumor cells out of $CD45^-$ cells from tumor mass as indicated left (n=6).
(J) Representative flow cytometry analysis of the ratio of tagBFP-Vehicle and tagGFP-Sh-Cbx4 Hepa1-6 tumor cells from tumor mass transplanted in C57/BL6 mice. Mice were treated with IgG control or anti-PD-1 antibody *in vivo*.
(K) Flow cytometry analysis of $CD8^+$ T (V) and NK (W) cell populations in NC and sgCbx4 tumors with or without $CD8^+$ T /NK cell depletion.
Data represent mean ± SEM. Statistical significance was performed through the utilization of 2-tailed unpaired Student's *t* test (E, F and H), with the corresponding results expressed as follows: $^{**}p < 0.01$, $^{***}p < 0.001$.

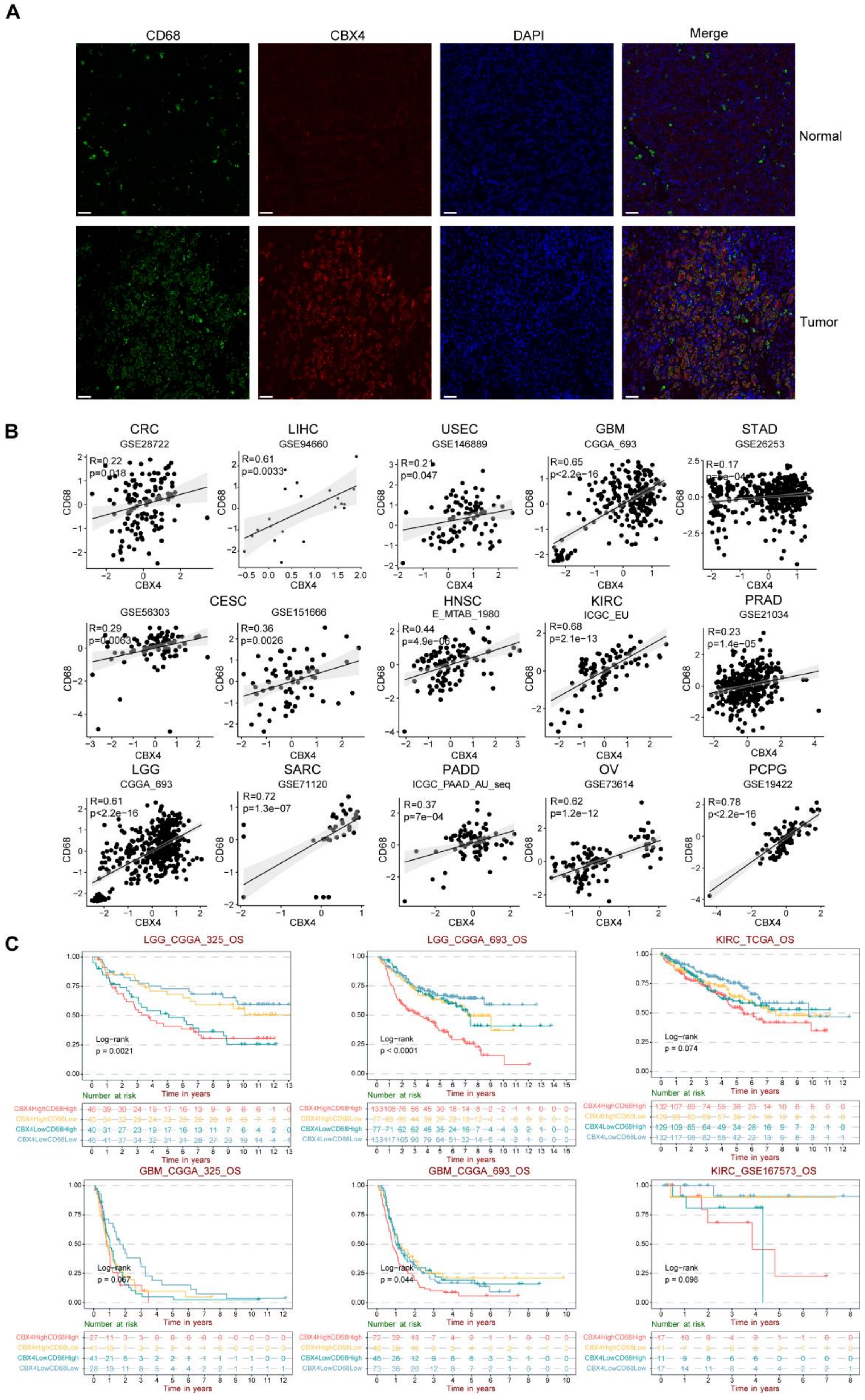
A
CD68
CBX4
DAPI
Merge
Normal
Tumor
B
CRC
GSE28722
R=0.22
p=0.018
LIHC
GSE94660
R=0.61
p=0.0033
USEC
GSE146889
R=0.21
p=0.047
GBM
CGGA_693
R=0.65
p<2.2e−16
STAD
GSE26253
R=0.17
CESC
GSE56303
R=0.29
p=0.0063
GSE151666
R=0.36
p=0.0026
HNSC
E_MTAB_1980
R=0.44
p=4.9e−06
KIRC
ICGC_EU
R=0.68
p=2.1e−13
PRAD
GSE21034
R=0.23
p=1.4e−05
LGG
CGGA_693
R=0.61
p<2.2e−16
SARC
GSE71120
R=0.72
p=1.3e−07
PADD
ICGC_PAAD_AU_seq
R=0.37
p=7e−04
OV
GSE73614
R=0.62
p=1.2e−12
PCPG
GSE19422
R=0.78
p<2.2e−16
CD68
CBX4
C
LGG_CGGA_325_OS
Log−rank
p = 0.0021
LGG_CGGA_693_OS
Log−rank
p < 0.0001
KIRC_TCGA_OS
Log−rank
p = 0.074
GBM_CGGA_325_OS
Log−rank
p = 0.067
GBM_CGGA_693_OS
Log−rank
p = 0.044
KIRC_GSE167573_OS
Log−rank
p = 0.098
Number at risk
Time in years
CBX4HighCD68High
CBX4HighCD68Low
CBX4LowCD68High
CBX4LowCD68Low

Figure. S9 CBX4 expression in macrophage is associated with tumor progression, recurrence, and patient outcome, relative to Figure.3.

(A) Representative multiplex immunohistochemistry images of clinical HCC and paracancerous tissues. F4/80 (Green), CBX4 (Red), DAPI (Blue). Scale bars, 50 μm.

(B) The correlation between CD68 and CBX4 in public data from TCGA database and GEO datasets.

(C) Kaplan-Meier analyses for overall survival (OS) for CBX4 and CD68 expressions in pan-cancer GEO datasets.

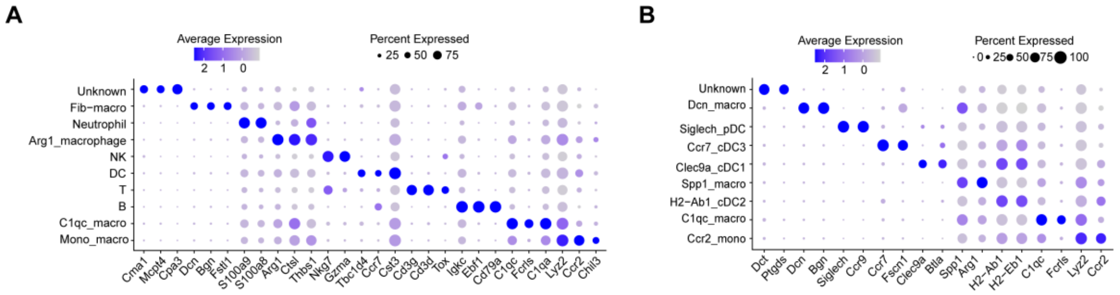

Figure.S10 Nomenclature of Single-cell Subpopulations, relative to Figure.3.

(A-B) Marker gene expression across defined cell clusters. Bubble size is proportional to the percentage of cells expressing a gene and color intensity is proportional to average scaled gene expression.

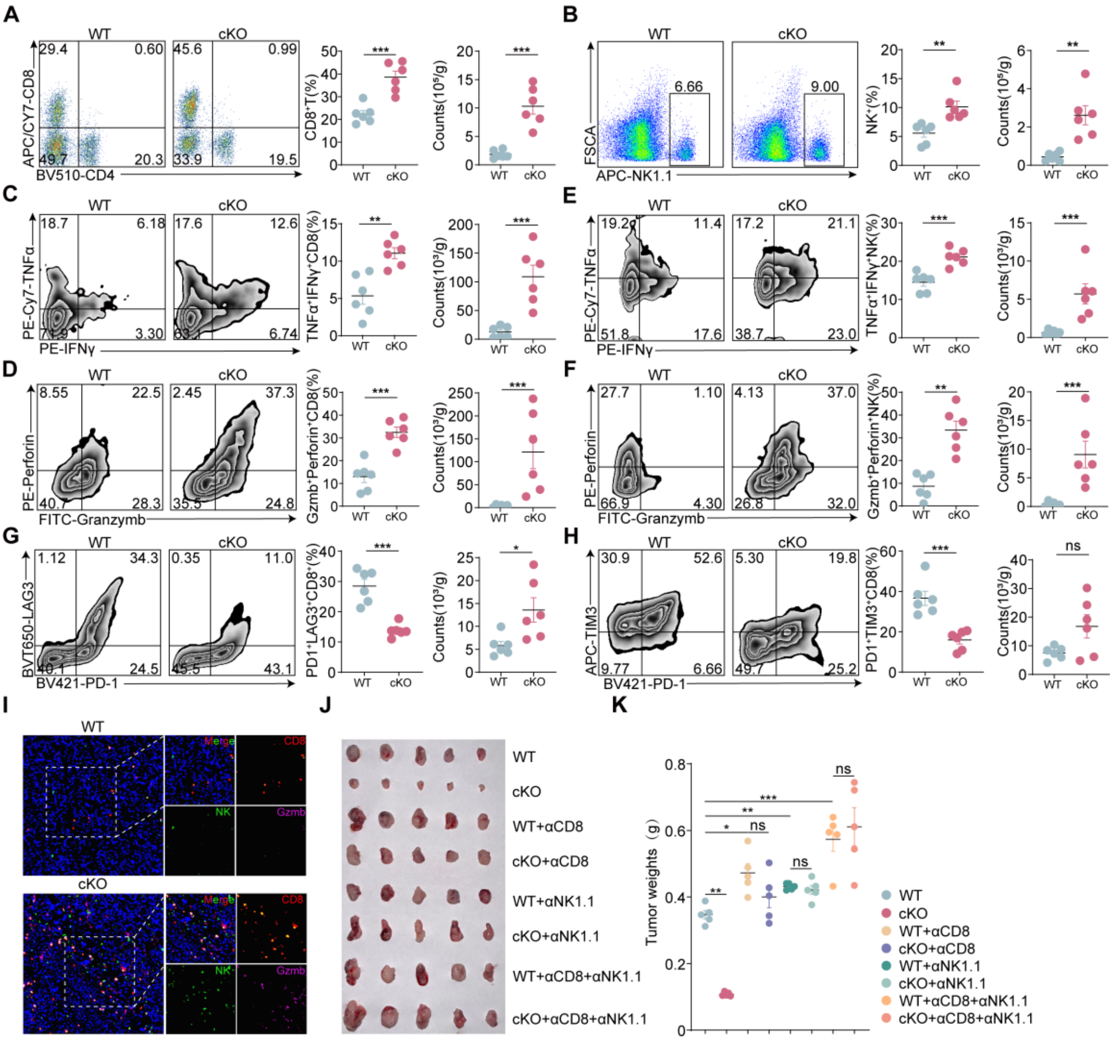

Figure. S11 Depletion of CBX4 in macrophages augmented the CD8^{+}T cells and NK cells anti-tumor immunity, relative to Figure.4.

(A-B) The percentage and numbers of CD8^{+}T and NK cells in WT and Cbx4 cKO B16 tumors as determined by flow cytometry (n=6).

(C-D) The percentage and numbers of TNF-α^{+}IFN-γ^{+} CD8^{+} T cells and Perforin^{+} granzyme B^{+} CD8^{+} T cells from WT and Cbx4 cKO B16 tumors (n=6).

(E-F) The percentage and numbers of TNF-α^{+}IFN-γ^{+} NK cells and Perforin^{+} granzyme B^{+} NK cells from WT and Cbx4 cKO B16 tumors (n=6).

(G-H) The percentage and numbers of PD1^{+} LAG3^{+} CD8^{+} T cells and PD1^{+} TIM3^{+} CD8^{+} T cells from WT and Cbx4 cKO B16 tumors (n=6).

(I) Representative images from WT and Cbx4 cKO Hepa1-6 tumor tissues. NK (Green), CD8 (Red), Gzmb (Purple), DAPI (Blue). Scale bars: 50 μm.

(J-K) Hepa1-6 tumor schematic diagram and tumor weight were assessed for the following groups: WT + IgG, Cbx4 cKO + IgG, WT + αCD8, Cbx4 cKO + αCD8, WT + αNK1.1, Cbx4 cKO + αNK1.1, WT + αCD8+ αNK1.1 and s Cbx4 cKO +αCD8+ αNK1.1 (n=5).

Data represent mean ± SEM. Data were analyzed by 2-tailed unpaired Student's *t* test (A-H) and one-way ANOVA (K), with the corresponding results expressed as follows: ns, non-significant, *$p < 0.05$, **$p < 0.01$, ***$p < 0.001$, ****$p < 0.0001$.

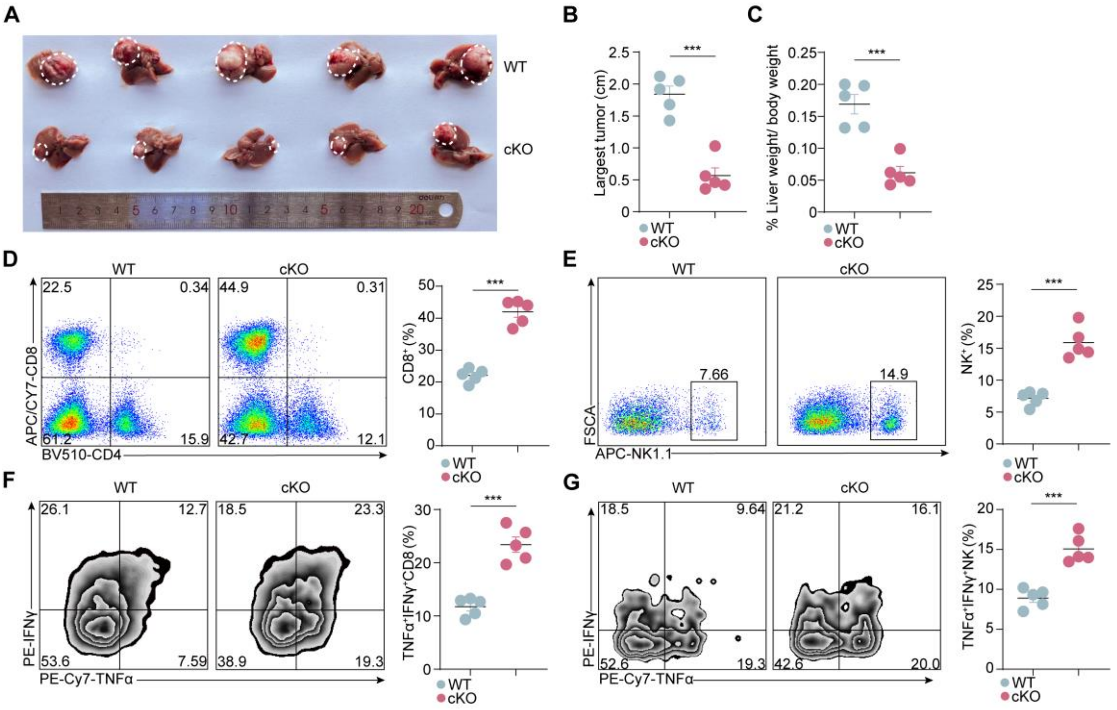

Figure. S12 In the orthotopic liver cancer model, depletion of CBX4 in macrophages augmented the CD8$^+$T cells and NK cells anti-tumor immunity, relative to Figure.4.

(A) The general images of orthotopic tumors derived from C57BL/6J mice in WT and Cbx4 cKO groups (n=5).

(B) The longest diameters of orthotopic tumors (n=5).

(C) The percentage of liver weight/body weight of orthotopic tumors (n=5).

(D-E) The percentage of CD8$^+$T and NK cells in WT and Cbx4 cKO Hepa1-6 orthotopic tumors as determined by flow cytometry (n=5).

(F-G) The percentage of TNF-α$^+$IFN-γ$^+$ CD8$^+$ T cells and TNF-α$^+$ IFN-γ$^+$CD8$^+$ NK cells in WT and Cbx4 cKO Hepa1-6 orthotopic tumors as determined by flow cytometry (n=5).

Data represent mean ± SEM. Statistical significance was performed through the utilization of 2-tailed unpaired Student's *t* test (B-G), with the corresponding results expressed as follows: ***$p < 0.001$.

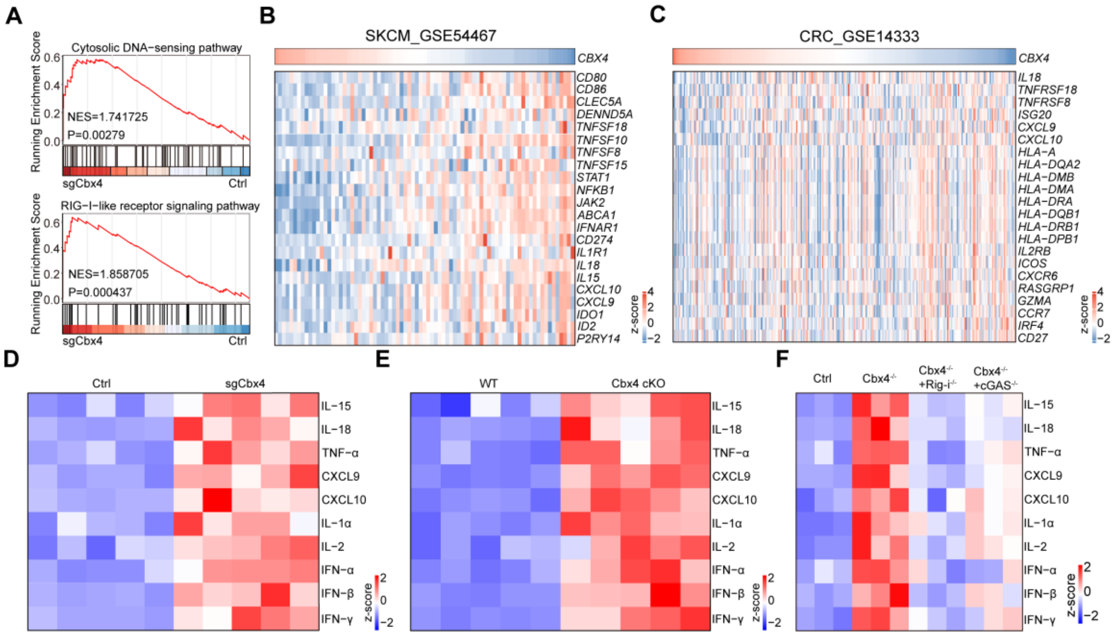

Figure. S13 Depletion of CBX4 activates the type-I interferon response through cytosolic RNA-sensing pathway, relative to Figure.5.

(A) GSEA plots of two top pathways induced by Cbx4 deletion in Hepa1-6 tumor cells from RNA-seq.

(B-C) Heatmap of scaled type-I interferon-related genes expression and innate immune-related genes expression in SKCM and CRC datasets, which were grouped by CBX4 expression.

(D) Heatmap of multi-cytokine profiling from Control and sgCbx4 tumor bearing mice.

(E) Heatmap of multi-cytokine profiling from WT and Cbx4 cKO tumor bearing mice.

(F) Heatmap of multi-cytokine profiling from Control, sgCbx4, sgCbx4+sgRig-i, sgCbx4+sgcGAS tumor bearing mice.

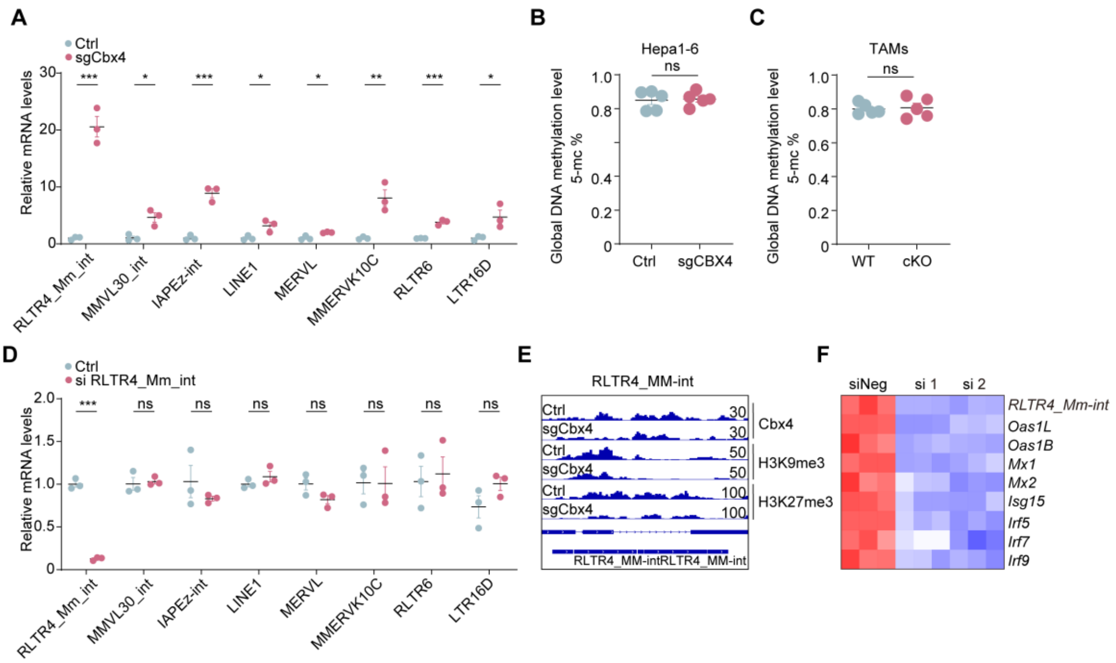

Figure. S14 Deletion of CBX4 derepresses endogenous retroelements, relative to Figure.6.

(A) qPCR analysis of retroelement loci expression in Ctrl/sgCbx4 Hepa1-6 tumor cells.
(B-C) The quantification of global DNA methylation levels of Control and sgCbx4 Hepa1-6 tumor cells, as well as WT and Cbx4 cKO CD11b^{high}F4/80^{high} TAMs.
(D) qPCR analysis of retroelement loci expression in Ctrl/si RLTR4_Mm_int Hepa1-6 tumor cells.
(E) IGV (Integrative Genomic Viewer) screenshots of Cbx4, H3K9me3 and H3K27me3 CUT-tag signals from Control and sgCbx4 Hepa1-6 tumor cells.
(F) Heatmap of scaled retroelement loci expression and type-I interferon-related genes expression in Cbx4 cKO TAMs with/without siRNA inhibition of RLTR4_Mm_int.
Data represent mean ± SEM. Statistical significance was performed through the 2-tailed unpaired Student's *t* test (A-D) with the corresponding results expressed as follows: ns, non-significant, **p* < 0.05, ***p* < 0.01, ****p* < 0.001.

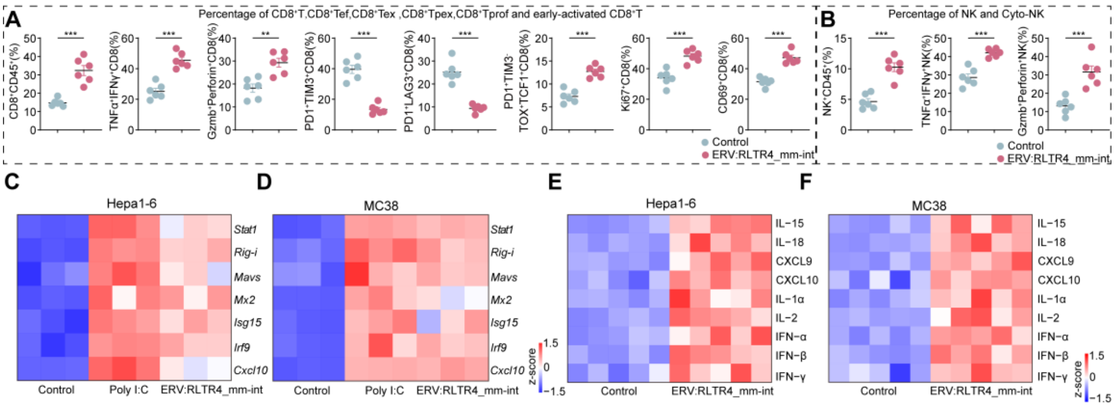

Figure. S15 ERV: RLTR4_Mm_int shapes the inflammatory microenvironment landscape, relative to Figure.6.

(A) The percentage of $CD8^{+}$T, $CD8^{+}$Tef (IFN-γ^{+} TNF-α^{+} and $Perforin^{+}$ granzyme B^{+}), $CD8^{+}$Tex ($PD1^{+}TIM3^{+}$ and $PD1^{+}LAG3^{+}$) and $CD8^{+}$Tpex ($PD1^{+}TIM3^{-}TOX^{+}TCF1^{+}$) in Control and ERV: RLTR4_Mm_int (5 nmol per intratumorally injection) Hepa1-6 tumors of the tumor-bearing mice as determined by flow cytometry (n=6).

(B) The percentage of NK and cyto-NK (IFN-γ^{+} TNF-α^{+} and $Perforin^{+}$ granzyme B^{+}) in Control and ERV: RLTR4_Mm_int (5 nmol per intratumorally injection) Hepa1-6 tumors of the tumor-bearing mice as determined by flow cytometry (n=6).

(C-D) Heatmap of relative expression of ISGs are shown. Hepa1-6/MC38 cells were transfected with or without Poly I:C and ERV: RLTR4_Mm_int as indicated. Data are representative of three experiments.

(E-F) Heatmap of multi-cytokine profiling from Control and ERV: RLTR4_Mm_int (5 nmol per intratumorally injection) Hepa1-6/MC38 tumor bearing mice (n=5).

Data represent mean ± SEM. Statistical significance was performed through the 2-tailed unpaired Student's t test (A-B) with the corresponding results expressed as follows: $^{**}p < 0.01$, $^{***}p < 0.001$.

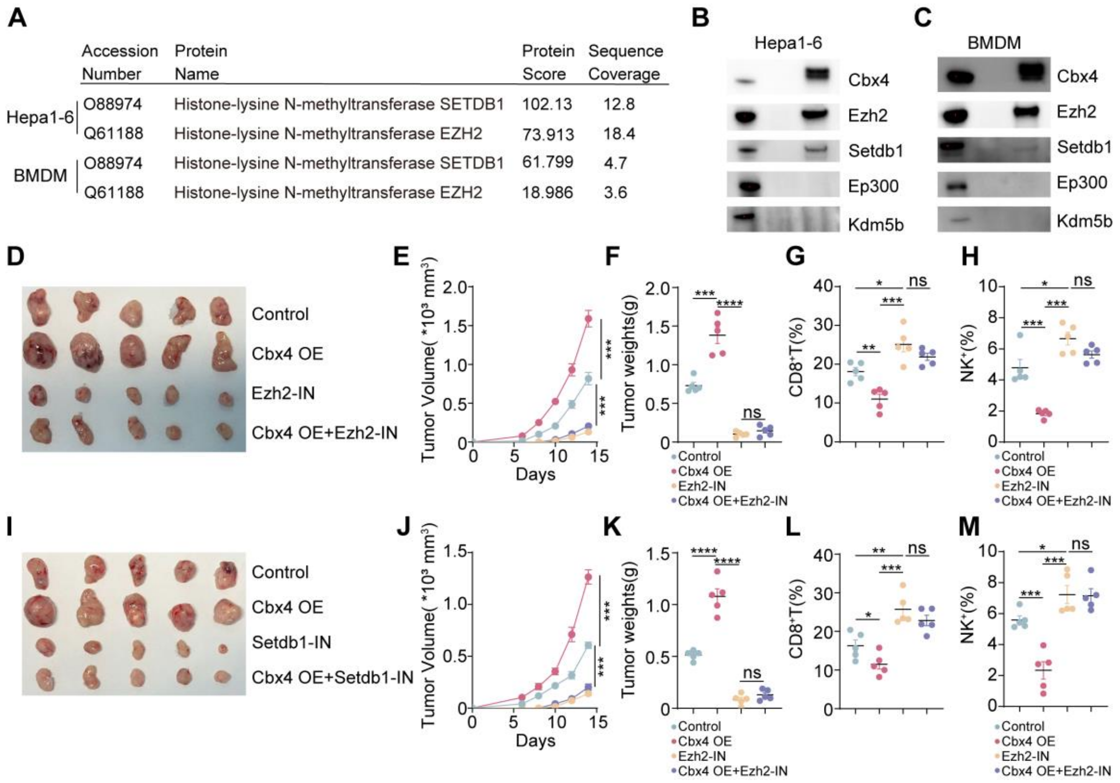

Figure. S16 Cbx4 interacts with the epigenetic regulators Ezh2 and Setdb1, relative to Figure.6.

(A) Selected Cbx4-binding proteins, protein score, and sequence coverage in mass spectrometry are listed.

(B-C) Immunoblot analysis of Cbx4, Ezh2, Setdb1, Ep300 and Kdm5b in cell lysates immunoprecipitated with anti-Cbx4 or IgG from Hepa1-6 tumor cells and BMDMs.

(D-F) Hepa1-6 tumor volume (D), tumor growth curve (E) and tumor weight (F) were assessed for the following groups: Control, Cbx4OE, Ezh2-IN, Cbx4 OE+Ezh2-IN (n=5).

(G-H) The percentage of CD8 (G) and NK(H) in Control, Cbx4OE, Ezh2-IN, Cbx4 OE+Ezh2-IN Hepa1-6 tumors at 14 days as determined by flow cytometry (n=5).

(I-K) Hepa1-6 tumor volume (I), tumor growth curve (J) and tumor weight (K) were assessed for the following groups: Control, Cbx4OE, Setdb1-IN, Cbx4 OE+Setdb1-IN (n=5).

(L-M) The percentage of CD8 (L) and NK(M) in Control, Cbx4OE, Ezh2-IN, Cbx4 OE+Ezh2-IN Hepa1-6 tumors at 14 days as determined by flow cytometry (n=5).

Data represent mean ± SEM. Tumor growth curves data were analyzed by two-way ANOVA with Tukey's multiple comparisons test (E, J). Other data were analyzed by one-way ANOVA (F-H and K-L), with the corresponding results expressed as follows: ns, non-significant, **p* < 0.05, ***p* < 0.01, ****p* < 0.001, *****p* < 0.0001.

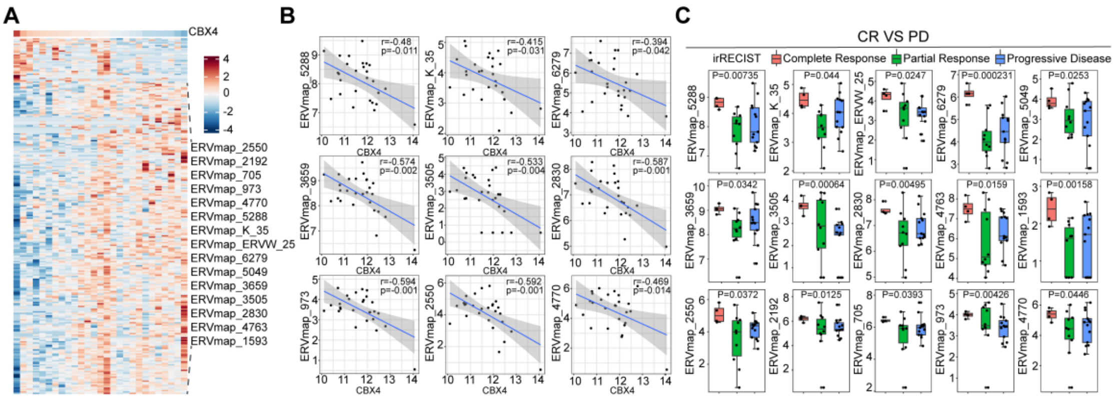

Figure. S17 Correlation analysis of human ERVs with CBX4 expression and selected epigenetic regulators with patient response to anti-PD-1 treatment, relative to Figure.6.

(A) Heatmap illustrating the expression of highly differential human endogenous retroviruses (ERVs) in patient biopsies prior to anti-PD-1 treatment, with samples ranked by CBX4 expression levels.

(**B)** *ERVmap* expression levels exhibit a negative correlation with CBX4 expression in the patient cohort described in panel R, with the gray zone representing the 95% confidence interval for the linear model prediction.

(C) Box plot showing ERVmap expression levels in the patient cohort described in panel R, with patient responses to anti-PD-1 treatment annotated (PD, progressive disease; PR, partial response; CR, complete response).

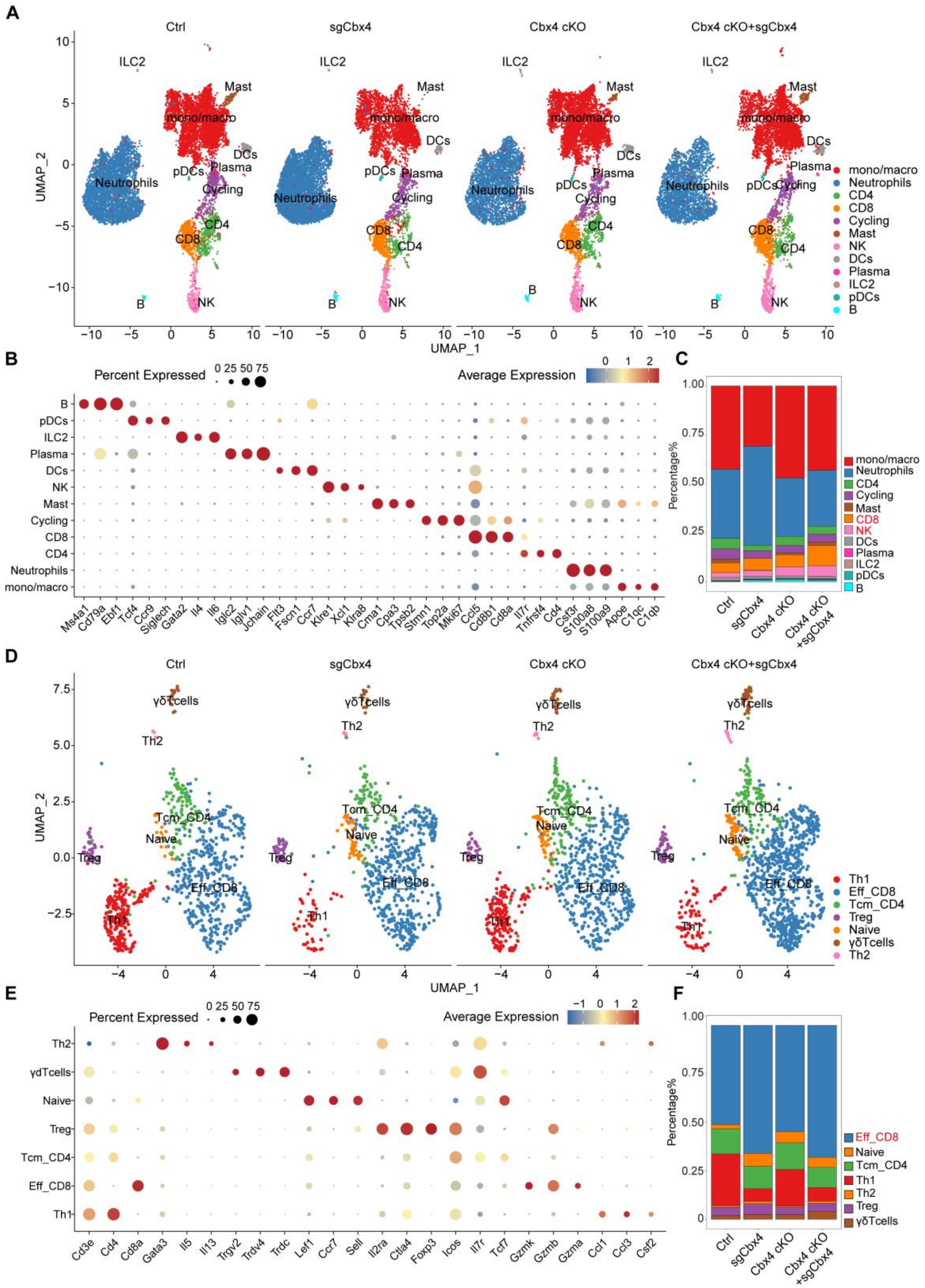

Figure. S18 Loss of Cbx4 in tumor cells and macrophages augmented the CD8^{+}T cells and NK cells anti-tumor immunity, relative to Figure.7.

(A) Uniform manifold approximation and projection (UMAP) maps of scRNA-seq of distinct clusters in the following groups: WT, sgCbx4, Cbx4 cKO, sgCbx4+Cbx4 cKO. (**B)** Marker gene expression across defined cell clusters. Bubble size is proportional to the percentage of cells expressing a gene and color intensity is proportional to average

scaled gene expression.

(C) The percentage of cell populations. The colour code corresponds to (A).

(D) Uniform manifold approximation and projection (UMAP) maps of scRNA-seq of T cell subpopulations in in the following groups: WT, sgCbx4, Cbx4 cKO, sgCbx4+Cbx4 cKO.

(E) Marker gene expression across defined cell clusters. Bubble size is proportional to the percentage of cells expressing a gene and color intensity is proportional to average scaled gene expression.

(F) The percentage of T cell subpopulation populations. The colour code corresponds to (D).

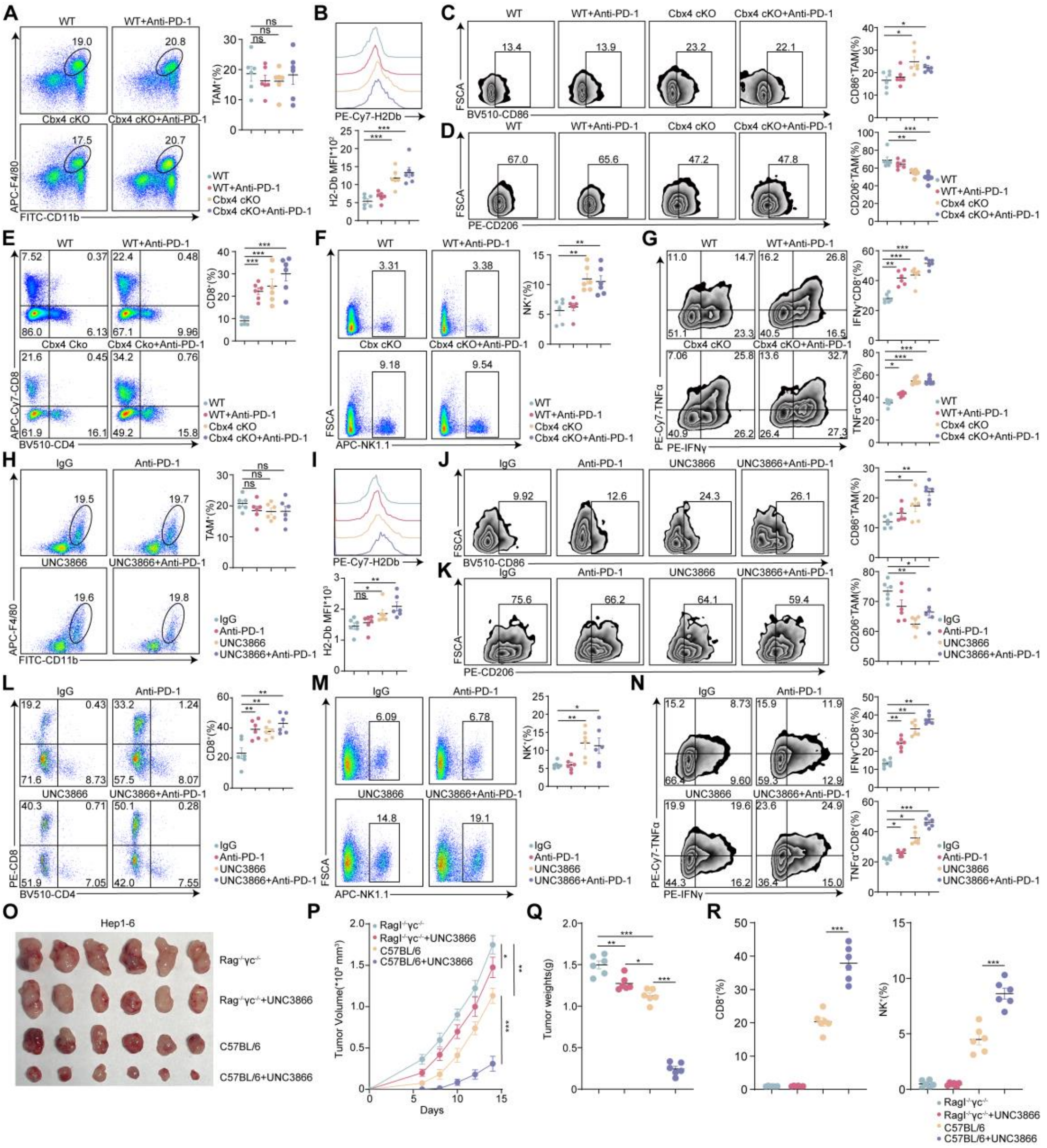

Figure. S19 Targeting Cbx4 strengthens the antitumor response of anti-PD1, relative to Figure.7.

(A) The percentage of $CD11b^{high}F4/80^{high}$ macrophages in WT, WT+ anti-PD1, Cbx4 cKO, anti-PD1+ Cbx4 cKO Hepa1-6 tumors as determined by flow cytometry (n=6).

(B) The Mean Fluorescence intensity (MFI) of H2-Db on $CD11b^{high}F4/80^{high}$ macrophages in WT, WT+ anti-PD1, Cbx4 cKO, anti-PD1+ Cbx4 cKO Hepa1-6 tumors as determined by flow cytometry (n=6).

(C) The percentage of CD86 on $CD11b^{high}F4/80^{high}$ macrophages in WT, WT+ anti-PD1, Cbx4 cKO, anti-PD1+ Cbx4 cKO Hepa1-6 tumors as determined by flow cytometry (n=6).

(D) The percentage of CD206 on $CD11b^{high}F4/80^{high}$ macrophages in WT, WT+ anti-PD1, Cbx4 cKO, anti-PD1+ Cbx4 cKO Hepa1-6 tumors as determined by flow cytometry (n=6).

(E-F) The percentage of $CD8^{+}$T and NK cells in WT, WT+ anti-PD1, CBX4 cKO, anti-

PD1+ Cbx4 cKO Hepa1-6 tumors as determined by flow cytometry (n=6).

(G) The percentage of TNF-α^+IFN-γ^+ CD8$^+$ T cells in WT, WT+ anti-PD1, Cbx4 cKO, anti-PD1+ Cbx4 cKO Hepa1-6 tumors as determined by flow cytometry (n=6).

(H) The percentage of CD11b^{high}F4/80^{high} macrophages in IgG, anti-PD1, UNC3866, anti-PD1+UNC3866 Hepa1-6 tumors as determined by flow cytometry (n=6).

(I) The Mean Fluorescence intensity (MFI) of H2-Db on CD11b^{high}F4/80^{high} macrophages in IgG, anti-PD1, UNC3866, anti-PD1+UNC3866 Hepa1-6 tumors as determined by flow cytometry (n=6).

(J) The percentage of CD86 on CD11b^{high}F4/80^{high} macrophages in IgG, anti-PD1, UNC3866, anti-PD1+UNC3866 Hepa1-6 tumors as determined by flow cytometry (n=6).

(K) The percentage of CD206 on CD11b^{high}F4/80^{high} macrophages in IgG, anti-PD1, UNC3866, anti-PD1+UNC3866 Hepa1-6 tumors as determined by flow cytometry (n=6).

(L-M) The percentage of CD8$^+$T and NK cells in IgG, anti-PD1, UNC3866, anti-PD1+UNC3866 Hepa1-6 tumors as determined by flow cytometry (n=6).

(N) The percentage of TNF-α^+IFN-γ^+ CD8$^+$ T cells in IgG, anti-PD1, UNC3866, anti-PD1+UNC3866 Hepa1-6 tumors as determined by flow cytometry (n=6).

(O-Q) Hepa1-6 tumor volume (O), tumor growth curve (P) and tumor weight (Q) were assessed for the following groups: Rag$^{-/-}\gamma$c$^{-/-}$, Rag$^{-/-}\gamma$c$^{-/-+}$UNC3866, C57BL/6, C57BL/6+UNC3866. (n=6).

(R) The percentage of CD8$^+$T and NK cells in Rag$^{-/-}\gamma$c$^{-/-}$, Rag$^{-/-}\gamma$c$^{-/-+}$UNC3866, C57BL/6, C57BL/6+UNC3866 Hepa1-6 tumors as determined by flow cytometry (n=6).

Tumor growth curves data were analyzed by two-way ANOVA with Tukey's multiple comparisons test (P). Data represent mean ± SD. Other data were analyzed by one-way ANOVA (A-N and Q-R), with the corresponding results expressed as follows: ns, non-significant, *$p < 0.05$, **$p < 0.01$, ***$p < 0.001$, ****$p < 0.0001$. Data represent mean ± SEM.

Supplement Methods

Clinical tissue samples

Three independent cohorts of HCC patients were recruited from the Hepatobiliary Center of Tongji Hospital, Huazhong University of Science and Technology (HUST), Wuhan, China. All samples were stored at -80°C. Cohort 2 comprised 108 pairs of HCC tissue samples collected from patients who underwent primary HCC resection at the Hepatic Surgery Center of Tongji Hospital, Wuhan, China. Samples from this cohort were used to construct microarrays for immunohistochemistry and multiplex immunofluorescence experiments. Cohort 3 comprised 50 pairs of HCC tissues (pairs consisting of tumor tissues and adjacent tissues from the same patient) for Western blot analysis of the CBX4 protein. Cohort 4 included 34 patients with HCC tissue samples after treatment with PD-1 antibody (tislelizumab) at Tongji Hospital, between December 2019 and September 2022. Samples from this cohort were used to construct microarrays for immunohistochemistry (IHC) experiments. No patient in Cohort 2, Cohort 3, or Cohort 4 received any other HCC treatment before surgery. Each specimen underwent histological and pathological examination and grading by three experienced pathologists.

Animal studies

Specific-pathogen-free C57BL/6J, NOD.Cg-Prkdc^{scid} Il2rg^{tm1Wjl} /SzJ (NSG) mice, OT-I mice and Rag$^{-/-}$ γc$^{-/-}$ mice were obtained from Charles River Laboratories (Beijing, China). *LysM-Cre* transgenic mice were obtained from Model Organisms (Shanghai, China) and subjected to genotyping. *Cbx4$^{fl/fl}$* mice were generated using the CRISPR–Cas9 system (GemPharmatech) and were then backcrossed with *LysM-Cre* transgenic mice to generate mice with selective deletion of Cbx4 in myeloid cells (*Cbx4$^{fl/fl}$ LysM-Cre*). For all tumor challenges, 6-12 week-old, age-matched mice were used and pre-specified end-points for tumor size were adhered to as defined by the Broad IACUC, including 2.0 cm in maximum dimension for validation studies and 2.5 cm in maximum dimension for screens with daily monitoring. All mice were housed under carefully controlled conditions (22°C, 50% humidity and 12-hour light/dark cycle starting at 7:00 AM).

Cell line and culture

The HEK293, B16F10, Hepa1-6, MC38 and Hepa1-6-OVA cell lines were acquired from the American Type Culture Collection (ATCC, Manassas, VA, USA). They were cultured in Dulbecco's modified Eagle's medium (DMEM) (Cat#: C11995500BT; Gibco) or RPMI-1640 (Procell, Cat# GPM150113) medium supplemented with 10% fetal bovine serum (FBS) (Gibco, Cat# 10099-141C) and 1% penicillin/streptomycin (New Cell & Molecular Biotech Co., Ltd., Shanghai, China).

Animal Models

For subcutaneous tumor formation, $5x10^5$ tumor cells were subcutaneously injected into C57BL/6 mice, and the tumor growth curve was measured by calculating the product of the length and width. At the end of the experiments, tumors were surgically dissected and tumor size was measured. For methylation inhibition experiments, male C57BL/6J mice were administered GSK126 (MCE, Cat# HY-13470, 25mg/kg per mouse, every 3 days) or SETDB1-TTD-IN-1 (TargetMol, Cat#: T9742, 10mg/kg per mouse, every 3 days) via intraperitoneal injection. For the orthotopic tumor formation assay, Hepa1-6 cells (1×10^6 cells in 30 μL serum-free DMEM) were injected into the left liver lobe of C57BL/6 male mice. For the in vivo $CD8^+$ T cell and NK cell depletion studies, 6-week-old male C57BL/6J mice were administered an anti-CD8 (αCD8α, Cat#: 210220, Selleck)/NK1.1 (αNK1.1, Cat#: 211410, Selleck) or IgG2a/IgG2b isotype control antibody twice a week. For anti-PD-1 monotherapy studies, 6-week-old male C57BL/6J mice were injected with $5x10^5$ tumor cells. Starting seven days after injection, mice received 10 mg/kg of anti-PD-1 monoclonal antibody (αPD-1, Cat#: 212227, Selleck) or an isotype control antibody (IgG, Cat#: 211610, Selleck) every three days. For PDX-huPBMC model: Human peripheral blood mononuclear cells (PBMCs) were intravenous inoculated into immunodeficient NSG mice at a dose of 5,000,000 cells/200 μL. PDX models were established by transplanting small tumor fragments quickly and directly from surgical specimens of specific HCC patients into the flanks subcutaneous tissues of hu-PBMC-NSG mice and recorded as Day0. About two weeks later, the mice were divided into four groups: IgG, UNC3866 (10mg/kg per mouse; intraperitoneal injection, every 2 days), Pembrolizumab (αPD-1, Cat#: 200513, Selleck)

(100 μg per mouse; intraperitoneal injection every 3 days), and UNC3866 + Pembrolizumab.

Bone marrow-derived macrophages (BMDM) isolation, *invitro* TAMs induction and *invivo* TAMs sorting

Bone marrow cells were isolated from 6-8-week-old male *Cbx4*$^{fl/fl}$ *LysM-Cre* and littermate mice and cultured in a sterile dish with complete macrophage medium composed of DMEM (Cat#: C11995500BT; Gibco), 10% FBS (Gibco, Cat# 10099-141C), 1% penicillin/streptomycin (New Cell & Molecular Biotech Co., Ltd., Shanghai, China) and 20 ng/ml M-CSF (Cat#: 315-02; Peprotech) for 6 days. When BMDMs were induced into M0 state (day 6), cells were cultured in a 1:1 ratio of 10% FBS DMEM and Hepa1-6 tumor-conditioned media to induce tumor-associated macrophages in vitro. Induced macrophages were used for further coculture experiments, RNA and protein isolation experiments. In the coculture experiments, OT-I CD8^{+} T cells were cultured with TAMs already pulsed with 10ug/ml OVA (Genescript, RP10611CN) to imitate the process of antigen presentation *in vivo*. To obtain *invivo* TAMs, tumors were harvested and on day 14. Live CD45^{+}CD11B^{high}F4/80^{high} tumor-associated macrophages (TAMs) were isolated using FACSAria II Cell Sorter (BD Biosciences). The sorted cells were used for RNA-seq and CUT&Tag experiments.

OT-I CD8^{+}T isolation and differentiation for in vitro assays and adoptive transfer into tumour-bearing mice

Naive CD8^{+} T cells were isolated from the spleens of OT-I mice using the Mouse CD8^{+} T Cell Isolation Kit (B90011, Selleck), in accordance with the manufacturer's protocol. Subsequently, the purified OT-I CD8^{+} T cells were activated with the T Cell Activation/Expansion Kit (Miltenyi Biotech, Cat# 130-093-627). Following activation, the cells were cultured for 3 days in RPMI-1640 medium (Procell, Cat# GPM150113), which was supplemented with 10% fetal bovine serum (Gibco, Cat# 10099-141C), 1% penicillin-streptomycin (Gibco, Cat# 15070063), and 50 μM mercaptoethanol (Thermo Fisher Scientific, Cat# 21985023), prior to subsequent experimentation. Preactivated OT-I CD8^{+} T cells were then cocultured with Hepa1-6 OVA tumour cells at a ratio of 5:1 in standard 10% RPMI 1640 cell medium for 24 hours. For adoptive transfer

experiment, Hepa1-6-OVA cells were subcutaneously implanted into the right flank of $Rag^{-/-}$ $\gamma c^{-/-}$ mice. At 6 days post-transplantation, the in vitro activated OT-1 T cells were intravenously injected into each tumor-bearing $Rag^{-/-}$ $\gamma c^{-/-}$ mouse.

NK-cell isolation, treatment and coculture with tumor cells.

NK cells were sorted from spleen using APC-labeled anti-NK1.1 antibody (Cat#: 156505; Biolegend) and anti-APC magnetic beads (Cat#: 130-090-855; MACS, Miltenyi) according to the manufacturer's recommendations. NK cells were cultured in RPMI-1640 medium (Procell, Cat# GPM150113) supplemented with 10% fetal bovine serum (Gibco, Cat# 10099-141C), 1% penicillin-streptomycin (New Cell & Molecular Biotech Co., Ltd., Shanghai, China), IL-2 (500 U ml^{-1}) and IL-15 (20 ng ml^{-1}; PeproTech) for 2 days before further experiments. Then NK cells were cocultured with Hepa 1-6 cells at a ratio of 5:1 in standard 10% RPMI 1640 cell medium containing IL-2/15 for 24 hours.

In vivo CRISPR screens

We constructed a pooled sgRNA library targeting 998 epigenetic-related genes (10 sgRNAs per gene) along with 400 non-targeting control guides into a lentiviral CRISPR vector (lentiCRISPR-v2-puro). The CRISPR library plasmids were transfected into HEK293 cells at 90% confluence in 15 cm tissue culture plates. Viral supernatants were collected at 48 and 72 hours post-transfection, filtered through a 0.45 μm filtration unit (Corning, Cat# 430770), aliquoted, and stored at -80°C for future use. Hepa1-6 and MC38 cells were cultured following standard protocols. For the pooled large-scale CRISPR screen, cancer cells were transduced with the lentivirus carrying the library at a multiplicity of infection (MOI) of 0.3. After 3 days of puromycin selection, approximately 30% of the surviving cells were stored as Day-0 input samples at -80°C, while the remaining cells were used for in vivo screenings. For tumor challenges, 1×10^6 cells from the pooled library were suspended in 100 μl PBS, then inoculated subcutaneously into the bilateral flanks of recipient mice. For the Hepa1-6 or MC38 screen, mice received 200 μg of anti-PD1 i.p. on days 6, 9, and 12. Tumors were harvested on day 14. For genomic DNA extraction, pooled tissue was first digested with Proteinase K (Qiagen) and buffer ATL (Qiagen). Genomic DNA was then extracted

using the FastPure® Cell/Tissue DNA Isolation Mini Kit (TD102, Vazyme Biotech, China). PCR amplification of the library-targeted regions was performed on genomic DNA to prepare the sequencing library. The PCR products were sequenced on an Illumina HiSeq platform. Each library was sequenced to achieve approximately 500-fold coverage of the CRISPR library. The resulting sequencing data were then analyzed using MAGeCK and MAGeCK-VISPR for downstream analysis.

Generation of CRISPR/Cas9 Knockout Cell Lines

Small guide RNAs (sgRNAs) targeting the Cbx4, Kdm8, Ezh2 and Kdm2a gene were designed using the Benchling CRISPR sgRNA design tools (https://benchling.com/crispr). The specific target sequences for Cbx4, Kdm8, Ezh2 and Kdm2a are listed in *Table Materials*. Lentiviral vectors were generated by co-transfecting the Leti-Cas9-puro vector and packaging plasmids into HEK293 cells. For the construction of Cbx4, Kdm8, Ezh2 and Kdm2a knockout (KO) cell lines, target cells were infected with the CRISPR-Cas9-sgRNA lentivirus and subsequently selected using puromycin. Puromycin-resistant clones were isolated, and knockout efficiency was confirmed by western blotting.

Construction of lentivirus and stable cell lines

Lentiviral vectors encoding mouse Cbx4, Kdm8, Ezh2, and Kdm2a were constructed by utilizing the pLV-puro backbone. An empty vector was employed as the negative control. The process of lentivirus production and cell infection was conducted in accordance with the protocol supplied by Addgene. Specifically, lentiviral plasmids, together with packaging plasmids pMD2.G and psPAX2 (Addgene plasmids #12259 and #12260), were transfected into HEK293 cells using Lipofectamine® 3000 transfection reagent (Thermo Fisher Scientific) and Opti-MEM (Thermo Fisher Scientific). The lentiviral supernatant was harvested on the 4th and 5th days and passed through a 0.45-μm filter before being stored at -80°C. Target cells were infected with lentiviral particles in media containing 5 μg/mL polybrene (Sigma, H9268). Seventy-two hours after infection, the cells were selected using 2.5 μg/mL puromycin for a period of 2 weeks, thereby resulting in stable cell pools that were subsequently utilized for further experiments.

Gene interference and *in vitro* dsRNA transcription

All siRNAs employed in this study were purchased from GENA-CREATE and are listed in *Table Materials*. RNA interference transfections into macrophages were performed according to the procedure for CALNP™ RNAi in vitro (D-Nano Therapeutics; cat # DN001- 05) and knockout efficiency was confirmed by RT-qPCR. siRNAs were transfected at 20 μM final concentration. Poly I:C was obtained from Sigma-Aldrich (cat #P9582). The genomic sequences of *RLTR4_Mm_int* were download from the mm39 database. The region (222 bp) was selected as the dsRNA *in vitro* transcription template sequence. The dsRNA was synthesized and purified according to the *In vitro* dsRNA Synthesis Kit instructions (Sangon Biotech, Cat. No. B639254, China). The transfection method of poly I:C and dsRNA were the same as that of siRNA transfection at a final concentration of 5 μg/ml.

Adoptive cell transfer

Naive $CD8^+$ T cells were isolated from the spleens of OT-I mice using the Mouse $CD8^+$ T Cell Isolation Kit (B90011, Selleck), in accordance with the manufacturer's protocol. Subsequently, the purified OT-I $CD8^+$ T cells were activated with the T Cell Activation/Expansion Kit (Miltenyi Biotech, Cat# 130-093-627). Following activation, the cells were cultured for 3 days in RPMI-1640 medium (Procell, Cat# GPM150113), which was supplemented with 10% fetal bovine serum (Gibco, Cat# 10099-141C), 1% penicillin-streptomycin (Gibco, Cat# 15070063), and 50 μM mercaptoethanol (Thermo Fisher Scientific, Cat# 21985023), prior to subsequent experimentation. Hepa1-6-OVA cells were subcutaneously implanted into the right flank of $Rag^{-/-}$ $\gamma c^{-/-}$ mice. At 6 days post-transplantation, the in vitro activated OT-1 T cells were intravenously injected into each tumor-bearing $Rag^{-/-}$ $\gamma c^{-/-}$ mouse.

Isolation of Tumor-infiltrating immune Cells

To purify the tumor-infiltrating immune cells, tumor-bearing mice were euthanized on day 14, and the tumors were dissected from the surrounding tissues. The isolated tumors were rinsed with PBS and subsequently incubated in DMEM containing 100 ng/mL of type IV collagenase (Sigma-Aldrich) at 37°C for 30 minutes. Following the digestion process, the tumor tissue was passed through a 70μm filter (Biosharp) under pressure.

The resulting cells were washed with PBS and then subjected to density gradient centrifugation using a 38% Percoll solution (GE Healthcare).

Flow Cytometry Analysis

Immune cells extracted from mice were used for flow cytometry analyses. This process involved incubating the cells with the respective antibodies at 4°C for 30 min, specifically for surface staining. The cells were then washed with a solution of phosphate-buffered saline (PBS) enriched with 0.2% bovine serum albumin (BSA) to remove any unbound antibodies. Cell surface staining was performed using the appropriate antibodies.

For the intracellular staining procedure, samples underwent initial surface marker labeling before being fixed and permeabilized in accordance with the protocol provided by the manufacturer (BD Biosciences, Part No. 562574). Then, the cells were being fixed and permeabilized at a low temperature in the dark for a duration of 30 minutes. Following this, the cells were suspended in a nuclear antigen staining solution and allowed incubate at a low temperature in the dark for a duration of 30 minutes. After incubation, the cells were subjected to washing with PBS to ready them for flow cytometric analysis.

IHC

Slides were rehydrated in xylene and ethanol after being deparaffinized for IHC. Following a 30-minute incubation period with 0.3% hydrogen peroxide, antigen retrieval was carried out using citrate buffer for 15 minutes at a temperature below boiling point, and the reaction was blocked for 60 minutes with 5% bovine serum albumin (BSA). After that, the slides were then incubated for one hour at 37°C with HRP-conjugated secondary antibodies and overnight at 4°C with primary antibodies. After that, the sections were incubated for color development using a 3, 3'-diaminobenzidine tetrahydrochloride kit (Gene Tech, Shanghai, China) and for nuclear counterstaining using hematoxylin. The program Case-Viewer (3DHISTECH, Budapest, Hungary) or a regular microscope (Olympus, Tokyo, Japan) were used to obtain the images. Staining intensity and area of staining were then independently scored by two pathologists who were unaware of the patient's clinical information.

In situ multi-color immunofluorescence staining and scoring

In situ multi-color immunofluorescence staining and analysis were performed on paraffin-embedded tissue sections using the OPAL multiplex fluorescent staining system (PerkinElmer). Sections were heated at 62℃ for 3 hours and dewaxed on a Leica Bond RX. Primary antibodies were applied at the manufacturer's suggested dilutions, followed by fluorescent labeling. Signal amplification and detection were achieved with Opal Polymer HRP-conjugated secondary antibodies paired with fluorophores Opal 480, Opal 520, Opal 570, Opal 620, Opal 690, and DAPI (Akoya Biosciences). Protein expression was quantified by staining intensity or positivity rate, with intensity scored as 0 (negative), 1 (weak), 2 (moderate), or 3 (strong). Semi-quantitative evaluation of positively stained cells was performed for each sample, and the histological score (H-score) was computed by multiplying the intensity score by the percentage of positive cells. The staining results were assessed independently by two reviewers. Imaging was conducted using the PerkinElmer Vectra multispectral slide scanning system.

Cell Lysis and Immunoblotting

For immunoblotting, cells were washed twice with DPBS and lysed on ice in RIPA lysis buffer supplemented with a protease inhibitor cocktail (Sigma, P8340). Protein concentrations were determined using the BCA protein assay kit (Beyotime, China), and samples were denatured at 95 °C for 5 min. To analyze protein expression, 20 µg of protein extract was separated by 10% SDS-PAGE and transferred to an Immunobilon membrane (Immobilon-P; Millipore, MA, USA). The membrane was blocked with 5% skim milk in TBST for 1 hour before incubation with primary antibodies overnight at 4°C. After washing, the membrane was incubated with HRP-conjugated secondary antibodies at room temperature for 1 hour. Protein bands were visualized using the Clarity™ Western ECL substrate (Bio-Rad, USA) and detected with a Bio-Rad GelDoc system (Bio-Rad, USA). Information on the antibodies used is provided in *Table Materials*.

Multiplex secretome analysis

Mouse sera were collected after treatment as indicated. Fluorescent-coded

microspheres (ABplex Mouse Multiplex Custom Panel, ABclonal Technology) were mixed, and the sera were added according to the manufacturer's instructions, with technical assistance from ABclonal Technology.

Global DNA methylation (5-mC) ELISA assay

Total DNA was isolated from the cell samples with the Blood & Cell Culture DNA Midi Kit (QIAGEN, Cat. No. 13343) for the purpose of DNA methylation analysis. The levels of global DNA hydroxymethylation (5hmC) were determined using the MethylFlash Global DNA Methylation (5-mC) ELISA Kit (EpiGentek, Cat. No. P-1030-96), following the protocol provided by the manufacturer.

Reverse Transcription Quantitative PCR

Total RNA was extracted from cells using an RNAfast200 kit (Fastagen), and cDNA was synthesized using the PrimeScript RT Reagent Kit (Takara). To assess gene expression levels, both genes of interest and the HPRT control were subjected to PCR analysis using SYBR Green mix (Vazyme). The transcript levels of the target genes were calculated as the ratio of their expression to that of HPRT. Fold changes in target gene expression were analysed by StepOne Software (Applied Biosystems) using the delta/delta CT method.

Single-cell RNA Sequencing and Data analysis

Tissue Processing

Tumor tissues were washed thrice with Hank's Balanced Salt Solution (HBSS) and then minced into small pieces. Tissues were enzymatically digested in a solution containing type I collagenase (1 mg/mL; Biosharp, BS163) and DNase I (0.1 mg/mL; Biosharp, BS137) using a gentleMACS Tissue Dissociator (Miltenyi Biotec) at 37°C, following the manufacturer's recommended program. Post-digestion, the tissue homogenate was passed through a 40 μm sterile mesh to obtain a single suspension, which was subsequently resuspended in red blood cell (RBC) lysis buffer and washed twice with cold PBS. Cell viability was assessed using trypan blue staining under a microscope. To enrich immune cells, the single-cell suspensions were incubated with an APC-conjugated anti-CD45 antibody (BioLegend) at 4°C for 30 minutes. Following washing, the $CD45^{+}$ immune cells were isolated using a FACSAria II Cell Sorter (BD

Biosciences), achieving a purity of over 95% and a viability of more than 85% post-sorting.

RT & Amplification & Library Construction

Single-cell suspensions were prepared at a concentration of 2×10^5 cells/mL in PBS (HyClone) and loaded onto a microwell chip using the Singleron Matrix® Single-cell Processing System. Barcoding beads were collected from the chip, and captured mRNA was reverse-transcribed into cDNA, followed by PCR amplification. The amplified cDNA was fragmented, ligated with sequencing adapters, and used to construct libraries according to the GEXSCOPE® Single-cell RNA Library Kit protocol (Singleron). Libraries were diluted to 4 nM, pooled, and sequenced on an Illumina NovaSeq 6000 platform with 150 bp paired-end reads.

Single-cell RNA Sequencing Data analysis

The Cell Ranger analysis pipeline (v7.1.0) was used to generate gene expression matrices with cell-level gene counts for each sample. The resulting gene expression matrices were imported into Seurat (v4) and merged for further analysis. Low-quality cells were filtered based on the following criteria: $500 \leq$ detected genes ≤ 8000, mitochondrial RNA content $\leq 15\%$, and log10(GenesPerUMI) > 0.7. Additionally, DoubletFinder was used to remove potential doublets. Non-linear dimensionality reduction was performed using UMAP to visualize the clustering results. Cluster biomarkers were identified using the 'FindAllMarkers' function. Cell subsets were defined based on cluster-specific highly expressed genes and pathway enrichment.

To analyze cell–cell interactions, we utilized CellPhoneDB (v.4.0.0), a curated database of ligand–receptor interactions that includes information on subunit composition and details of monomeric or heteromeric complexes. CellPhoneDB is integrated into a statistical framework that allows the inference of significant ligand–receptor interactions between different cell types in single-cell RNA sequencing (scRNA-seq) data. After identifying the distinct cell types, we performed the cell–cell interaction analysis using a local installation of CellPhoneDB, following the recommended procedures for data preparation.

Cleavage Under Targets and Tagmentation (Cut&Tag)

Manual CUT&Tag reactions were performed according to the CUT&Tag-direct protocol (Vazyme, TD904-1). Briefly, cells were bound using Concanavalin A-coated Magnetic Beads Pro (ConA Beads Pro), and cell membrane permeabilization was achieved with the non-ionic detergent Digitonin. Targeted cleavage of the DNA sequence adjacent to the target protein was accomplished through the mediation of a primary antibody specific to the target protein, a corresponding secondary antibody, and Protein A/G. A transposon fused with Protein A/G was employed for precise targeting, and during the cleavage process, adapter sequences were added to both ends of the cleaved fragments. After PCR amplification, these fragments were transformed into a library suitable for direct high-throughput sequencing.

Bulk RNA sequencing and Data Analysis

Total RNA was extracted from TAMs and Hepa1-6 cells with TRIzol (Invitrogen). Total RNA (2 μg) was used for stranded RNA sequencing library preparation with a Stranded mRNA Library Prep Kit from DR08502 (Bioyigene) according to the manufacturer's instructions. The library products corresponding to 200-500 bp were enriched, quantified, and finally sequenced on DNBSEQ-T7. The gene expression profiles of TAMs from *Cbx4*$^{fl/fl}$ and *Cbx4*$^{fl/fl}$*LysM-Cre* mice, as well as Hepa1-6 Control cells and Cbx4 sgRNA cells after various treatments were determined by RNA-Seq data analysis (Bioyigene). In brief, raw sequencing data were first filtered by FastQC; low-quality reads were discarded, and adaptor sequences were trimmed. After quality filtering, each sample had ~49.5-67.5 million clean reads. Clean reads from each sample were mapped to the *Mus musculus* GRCm39 reference genome using HISAT2. Significantly differentially expressed transcripts were screened by applying the criteria FC>=2 or <=−2 and P value < 0.05. Gene ontology (GO) and Kyoto Encyclopedia of Genes and Genomes (KEGG) pathway analyses were performed using the Database for Annotation, Visualization, and Integrated Discovery (DAVID). Gene set enrichment analysis (GSEA) was conducted to identify whether DEGs were enriched in specific phenotypes or signaling pathways.

Transposable element (TE) analyses

The repeatmasker annotation file for mm39 was obtain using UCSC table browser. Create mm39 gene reference sequence files. Sequencing fastq files were aligned to the reference genome using Bowtie2 software and converted to bam files using SAMtools software. SAMtools was used for read quantification, resulting in quantification data. Repeat sequences with zero expression in at least 2 samples were removed. The DESeq2 package was used for normalization analysis. Dfam release 3.3 was used for annotation of transposable element families as LTR (further subtyped into ERV1, ERVK, ERVL) SINE, LINE, or DNA.

Supplement Materials

REAGENT or RESOURCE	COMPANY	IDENTIFIER
**Antibodies and Dyes**		
PE/Cyanine7 anti-mouse/human CD11b Antibody (clone M1/70)	Biolegend	cat#:101216; RRID: AB_312799
Brilliant Violet 785™ anti-mouse CD45 Antibody (clone 30-F11)	Biolegend	cat#:103149; RRID: AB_2564590
APC anti-mouse NK-1.1 Antibody (clone S17016D)	Biolegend	cat#: 156505; RRID: AB_ 2876525
APC anti-mouse F4/80 Antibody (clone BM8)	Biolegend	cat#:123116; RRID: AB_893481
FITC anti-mouse CD19 Antibody (clone 1D3/CD19)	Biolegend	cat#: 152403; RRID: AB_ 2629812
APC/Cyanine7 anti-mouse CD8a Antibody (clone 53-6.7)	Biolegend	cat#:100713; RRID: AB_312752
Brilliant Violet 510™ anti-mouse CD4 Antibody (clone RPA-T4)	Biolegend	cat#:300546; RRID: AB_2563314
PE/Cyanine7 anti-mouse H-2D^{b} Antibody (clone 28-8-6)	Biolegend	cat#: 114615; RRID: AB_ 2750195
Brilliant Violet 510™ anti-mouse CD86 Antibody (clone GL-1)	Biolegend	cat#:105039; RRID: AB_ 2562370
PE anti-mouse CD206 Antibody (clone C068C2)	Biolegend	cat#:141705; RRID: AB_10895754
PE anti-mouse IFNγ Antibody (clone XMG1.2)	Biolegend	cat#: 505807; RRID: AB_ 315401
PE/Cyanine7 anti-mouse TNFα Antibody (clone MP6-XT2)	Biolegend	cat#: 506323; RRID: AB_ 2256076
PE anti-mouse Perforin Antibody (clone S16009B)	Biolegend	cat#: 154405; RRID: AB_ 2721640
FITC anti-mouse GranzymeB Antibody (clone GB11)	Biolegend	cat#: 515403; RRID: AB_ 2114575
PE/Cyanine7 anti-mouse/human CD44 Antibody (clone IM7)	Biolegend	cat#: 103027; RRID: AB_ 830784
Brilliant Violet 605™ anti-mouse CD274 (B7-H1, PD-L1) Antibody (clone 10F.9G2)	Biolegend	cat#:124321; RRID: AB_2563635
Brilliant Violet 421™ anti-mouse CD279 (PD-1) Antibody (clone 29F.1A12)	Biolegend	cat#: 135217; RRID: AB_ 2561447
Brilliant Violet 650™ anti-mouse CD223 (LAG-3) Antibody (clone C9B7W)	Biolegend	cat#: 125227; RRID: AB_ 2687209
APC anti-mouse CD366 (Tim-3) Antibody (clone B8.2C12)	Biolegend	cat#: 134007; RRID: AB_ 2562997

TruStain FcX™ (anti-mouse CD16/32) Antibody (clone 93)	Biolegend	cat#:101320; RRID: AB_1574975
Rabbit monoclonal [EPR23053-7] to CBX4 - ChIP Grade	Abcam	cat#: ab242149
CD8a Monoclonal Antibody (53-6.7)	Invitrogen Antibodies	cat#: 14-0081
Rabbit monoclonal [EP2567Y] to NCAM1	Abcam	cat#: ab75813
Mouse monoclonal [NAT105] to PD1	Abcam	cat#: ab52587
Rabbit monoclonal [EPR20545] to CD68	Abcam	cat#: ab213363
Rabbit polyclonal to Mannose Receptor	Abcam	cat#: ab64693
Mouse monoclonal [C-11] to pan Cytokeratin	Abcam	cat#: ab7753
Rat monoclonal [CI: A3-1] to F4/80 - Macrophage Marker	Abcam	cat#: ab6640
Granzyme B (D2H2F) Rabbit mAb	CST	cat#: 17215
Double-stranded RNA (dsRNA) Antibody (J2), mAb, Mouse	Genecreate	cat#: A02181
Tri-Methyl-Histone H3 (Lys9) (D4W1U) Rabbit mAb	CST	cat#:13969S
Tri-Methyl-Histone H3 (Lys27) (C36B11) Rabbit mAb	CST	cat#:9733S
STAT1 Antibody	CST	cat#: 9172
Phospho-STAT1 (Tyr701) (58D6) Rabbit mAb	CST	cat#: 9167
KDM5B Rabbit pAb	Abclonal	cat#: A7772
EZH2/KMT6 Rabbit pAb	Abclonal	cat#: A16846
NF-kB p65/RelA Rabbit mAb	Abclonal	cat#: A19653
Phospho-NF-kB p65/RelA-S311 Rabbit pAb (AP0445)	Abclonal	cat#: AP0445
IRF3 Rabbit pAb	Abclonal	cat#: A19717
Phospho-IRF3 Rabbit mAb	Abclonal	cat#: AP1412
IRF7 Rabbit mAb	Abclonal	cat#: A22742
Phospho-IRF7-S471/472 Rabbit pAb (AP0445)	Abclonal	cat#: AP1264
cGAS Mouse mAb	Abclonal	cat#: A27100
STING/TMEM173 Rabbit pAb	Abclonal	cat#: A3575
RIG-1/DDX58 Monoclonal antibody	Proteintech	cat#: 3B10F5
MAZ Polyclonal antibody	Proteintech	cat#: 21068-1-AP
p300 Polyclonal antibody	Proteintech	cat#: 20695-1-AP
SETDB1 Polyclonal antibody	Proteintech	cat#: 11231-1-AP
MAVS Rabbit pAb	Abclonal	cat#: A5764
GAPDH Rabbit pAb	Abclonal	cat#: AC001

DAPI	Servicebio	cat#: G1012
Goat anti-Rabbit IgG (H+L) Secondary Antibody, HRP	Invitrogen	cat#: 31460;<br>RRID: AB_228341
**Chemicals, Peptides, and Recombinant Proteins**		
Recombinant Murine M-CSF	Peprotech	cat#: 315-02
TRIzol	Invitrogen	cat#: 15596018
Collagenase II	Sigma-Aldrich	cat#: C2-BIOC
Collagenase IV	Sigma-Aldrich	cat#: C4-BIOC
Anti-mouse CD8α-InVivo	Selleck	cat#: 210220
Anti-mouse NK1.1-InVivo	Selleck	cat#: 211410
Anti-mouse PD-1 (CD279)-InVivo	Selleck	cat#: 212227
GSK126	MCE	cat#: HY-13470
SETDB1-TTD-IN-1	TargetMol	cat#: T9742
Phosphate buffer saline	Meilunbio	cat#: MA0016
PrimeScript RT Master Mix (Perfect Real Time)	Takara	cat#: RR036A
ChamQSYBR qPCR Master Mix (High ROX Premixed)	Vazyme	cat#: Q341-02
**Critical Commercial Assays**		
MethylFlash Global DNA Methylation (5-mC) ELISA Easy Kit	Epigentek	cat#: P-1030-48
Hyperactive Universal CUT&Tag Assay Kit for Illumina Pro	Vazyme	cat#: TD904-01
**Software and Algorithms**		
GraphPad Prism 8	GraphPad Software	http://www.graphpad.com
FlowJo (version 10.3)	LLC	http://www.flowjo.com
ImageJ	NIH software	https://imagej.nih.gov/ij/
Cell Ranger (v7.1.0)	10x Genomics	https://10xgenomics.com.RRID: SCR_017344;
Seurat (v4.3.0		
Monocle2 (v2.29.0)	Qiu et al., 2017	https://github.com/cole-trapnelllab/monocle-release RRID:SCR_01633;
SingleR (v2.3.7)		
clusterProfiler (v4.9.3)	NA	https://bioconductor.org/packages/release/bioc/html/clusterProfiler.html
R (v4.3.1)	NA	https://www.r-project.org/

GSEA (v6.0)	Subramanian et al., 2005; Mootha et al., 2003	https://www.gsea-msigdb.org/gsea/index.jsp. RRID:SCR_003199.
CaseViewer	3DHISTECH	https://www.3dhistech.com/solutions/caseviewer/; RRID: SCR_017654
**Chemicals, Peptides, and Recombinant Proteins**		
Mouse: C57BL/6J (C57BL/6JNifdc)	Charles River Laboratories	N/A
Mouse: LysM-Cre	Model Organisms	N/A
Mouse: *Cbx4*$^{fl/fl}$	Model Organisms	N/A
Mouse: NOD SCID Il2rg$^{-/-}$	Charles River Laboratories	N/A
Mouse: OT-1	Charles River Laboratories	N/A
**Sequences of primer sets used for real-time quantitative PCR**		
**Gene**	**Forwardprimer,5-3**	**Reverseprimer,5-3**
h-β-actin	CATGTACGTTGCTATCCAGGC	CTCCTTAATGTCACGCACGAT
h-OASL1	CTGATGCAGGAACTGTATAGCAC	CACAGCGTCTAGCACCTCTT
h-DHX58	GGGCCTCCAAACTCGATGG	TTCTGGGGTGACATGATGCAC
h-MX1	GTTTCCGAAGTGGACATCGCA	CTGCACAGGTTGTTCTCAGC
h-IRF5	GGGCTTCAATGGGTCAACG	GCCTTCGGTGTATTTCCCTG
h-IRF7	GCTGGACGTGACCATCATGTA	GGGCCGTATAGGAACGTGC
h-IRF9	GCCCTACAAGGTGTATCAGTTG	TGCTGTCGCTTTGATGGTACT
h-MDA5	TCGAATGGGTATTCCACAGACG	GTGGCGACTGTCCTCTGAA

h-MAVS	CAGGCCGAGCCTATCATCTG	GGGCTTTGAGCTAGTTGGCA
h-CXCL9	CCAGTAGTGAGAAAGGGTCGC	AGGGCTTGGGGCAAATTGTT
h-CXCL10	GTGGCATTCAAGGAGTACCTC	TGATGGCCTTCGATTCTGGATT
h-HLA-B	CAGTTCGTGAGGTTCGACAG	CAGCCGTACATGCTCTGGA
h-HLA-DMA	CCTGCACACAGTGTACTGC	CACCCGAGTGTTCTGGGAA
h-HLA-A	GACCAGGAGACACGGAATGTG	CCTCGTTCAAGGCGATGTAATC
h-B2M	GAGGCTATCCAGCGTACTCCA	CGGCAGGCATACTCATCTTTT
h-IL18	TCTTCATTGACCAAGGAAATCGG	TCCGGGGTGCATTATCTCTAC
h-CBX4	GCAGAGTGGAGTATCTGGTGA	AGCTTGGCACGGTTGTCAG
h-CGAS	TAACCCTGGCTTTGGAATCAAAA	TGGGTACAAGGTAAAATGGCTTT
h-STING1	CACTTGGATGCTTGCCCTC	GCCACGTTGAAATTCCCTTTTT
m-*Hprt*	TCAGTCAACGGGGGACATAAA	GGGGCTGTACTGCTTAACCAG
m-*Cbx4*	AAGAAGCGGATACGCAAGGG	GGAGGAGTCTTGAAGCCCAG
m-*cGAS*	GTTCAAACACAAGAAATGCACTG	GCTGACGGAGTACACAATCCT
m-*Sting1*	CCTAGCCTCGCACGAACTTG	CGCACAGCCTTCCAGTAGC
m-*Mavs*	AGGGTGGGATGGACTGAGAT	CTAGGGGAGAATGAGGTCGG
m-*Rig-I*	GAGTACCACTTAAAGCCAGAG	AATCCATTTCTTCAGAGCATCC

m-*Mda5*	CGGAAGTTGGAGT CAAAGC	TTTGTTCAGTCTGAGTC ATGG
m-*Stat1*	CGCGCATGCAACT GGCATATAACT	ATGCTTCCGTTCCCACG TAGACTT
m-*Irf5*	GGTCAACGGGGAA AAGAAACT	CATCCACCCCTTCAGTG TACT
m-*Irf7*	CACAGTCTTCCGCG TACCC	TCCCGGCTAAGTTCGTA CACC
m-*Irf9*	TTCATCTATGGTGG CCGAGT	ACGCCTCTGTCAAGCTG ATT
m-*Ifi30*	GGCTGACTCTGAC AGGTGGT	CTTTCGCAAGCATCCTC C
m-*Oasl1*	CAGGAGCTGTACG GCTTCC	CCTACCTTGAGTACCTT GAGCAC
m-*Isg15*	GGTGTCCGTGACTA ACTCCAT	TGGAAAGGGTAAGACC GTCCT
m-*Oas1b*	GGCCTCTAAGGGG GTCAAG	CTGGCAGCACGTCAAA CTTC
m-*Mx1*	GACCATAGGGGTC TTGACCAA	AGACTTGCTCTTTCTGA AAAGCC
m-*Mx2*	GAGGCTCTTCAGA ATGAGCAAA	CTCTGCGGTCAGTCTCT CT
m-*Maz*	GCCCCAGTTGCATC TGTCTT	CTTCGGAGGTTGTAGCC GTT
m-*E2f1*	CTCGACTCCTCGCA GATCG	GATCCAGCCTCCGTTTC ACC
m-*Usf1*	CTGAAACCGAAGA GGGAACAG	GTTGGGGTCAGGAAAA GTGG
m-*Patz1*	GGCTGCTACACCTA CCAGG	TGCGTTGTTGGTTCAGG TTGT
RLTR4-MM-int	GACCAGCTAGACC AGCCAGTGA	AGGAGTTGTCGCCGCCT TGA

MMVL30-int	CTGGCTGCTCTCTGGAGTTGGA	CTCGTGTCCGCTCTTGTTGTGA
IAPEz	AAGCAGCAATCACCCACTTTGG	CAATCATTAGATGTGGCTGCCAAG
LINE1	TTTGGGACACAATGAAAGCA	CTGCCGTCTACTCCTCTTGG
MERVL	ATCTCCTGGCACCTGGTATG	AGAAGAAGGCATTTGCCAGA
MMERVK10C	CAAATAGCCCTACCATATGTCAG	GTATACTTTCTTCTTCAGGTCCAC
RLTR6_Mm	CTCTAGATGGGAGGGGTCG	CTCACCTCAGAGCCCGAC
LTR16D	ACTCATTCTTTCCTTGCTCCAT	TTGCCTCTGCCTCACCAC
m-*Cbx4*-P1	GCTTTTGGGGGTTCACTTCTGC	CAAAAGAGGAGAGTGGGTCGAC
m-*Cbx4*-P2	AGCTGGGGCCCCGGACGCA	GCCTGGGAGATCCGTGGGG
m-*Cbx4*-P3	GCGGGGCGGTGGGCACCGAG	CCCTGCTGGGGGCCACGGA
LysM cre genotyping	AGTGCTGAAGTCCATAGATCGG	CTGATTCTCCTCATCACCAGG
Cbx4$^{fl/fl}$ genotyping	GAGGCAGGGCCTTCAAAGATTG	CTGAGGACTTCTGGGACATCTGCT

siRNA, shRNA and sgRNA used for knock down and knock out

sgCtrl	GCGAGGTATTCGGCTCCGCG
sgCbx4	ACTTGCAAGATATAACACGT

sgKdm8	GGCAGCCGAAGGAATATACC	
sgEzh2	TATCGTAGTAAGTACCAATG	
sgKdm2a	CTGGAGTCCCTATAGTACAG	
sgcGAS	ACGCAAAGATATCTCGGAGG	
sgSting1	TACCTTGGTAGACAATGAGG	
sgRig-i	CGTTGGAGATGCTAAGACCG	
sgMavs	GCCACCAGACATCCTCGCGA	
si-*cGAS*	GGAUUGAGCUACAAGAAUATT	UAUUCUUGUAGCUCAAUCCTT
si-*sting1*	GAGCUUGACUCCAGCGGAATT	UUCCGCUGGAGUCAAGCUCTT
si-*Rig-I*	CCACAAACUUGGAGAGUCATT	UGACUCUCCAAGUUUGUGGTT
si-*Mavs*	CCAAGAAGAGGAAGAACAUTT	AUGUUCUUCCUCUUCUUGGTT
si-RLTR4_MM-int-1	AGACAGAAUUUCGGUAGUATT	UACUACCGAAAUUCUGUCUTT
si-RLTR4_MM-int-2	GGUUCUGACCCAACAGUAUTT	AUACUGUUGGGUCAGAACCTT
si-Maz	GCAAGGACCGCAUGAGUUATT	UAACUCAUGCGGUCCUUGCTT
Cbx4-oe	TCGGCGGCCGCATGGAGCTGCCAGCTGTT	TCTGCTAGCTTACACCGTCACGTATTCCTT
shCbx4	CCGGCGTGATCGTTATGAGCAAGTACTCGAGTACTTGCTCATAACGATCACGTTTTTG	AATTCAAAAACGTGATCGTTATGAGCAAGTACTCGAGTACTTGCTCATAACGATCACG

**ERV: RLTR4_Mm_int Sequences (5’-3’)**
ACTGCCCCCGCCCTGGGATTGCCAGATTTGACTAAGCCCTTTGAACTCTTTGTCGACGA AAAGCAGGGCTACGCCAAAGGCGTCCTAACGCAAAAACTGGGACCTTGGCGTCGGCC TGTGGCCTACCTGTCCAAAAAGCTAGACCCAGTGGCAGCTGGGTGGCCCCCTTGCCTA CGGATAATAGCAGCCATTGCCGTTCTGACAAAAGATGCAGGCAATGCT
