# Supplementary figures and images for: In vivo CRISPR screens identify CBX4 as an epigenetic regulator for cancer immunotherapy
Source: J Clin Invest. 2026 Mar 31;136(10):e200564. doi: 10.1172/JCI200564 (PMC13178658; doi:10.1172/JCI200564)

Fig.5E

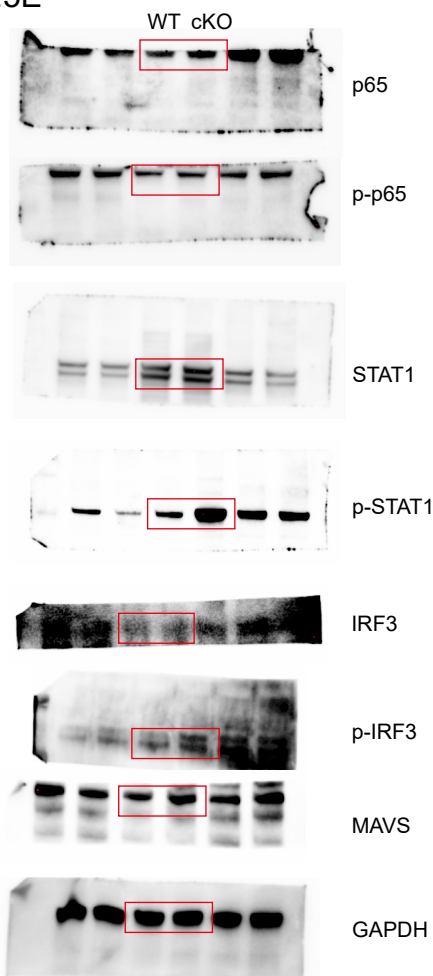

Fig.6G and H

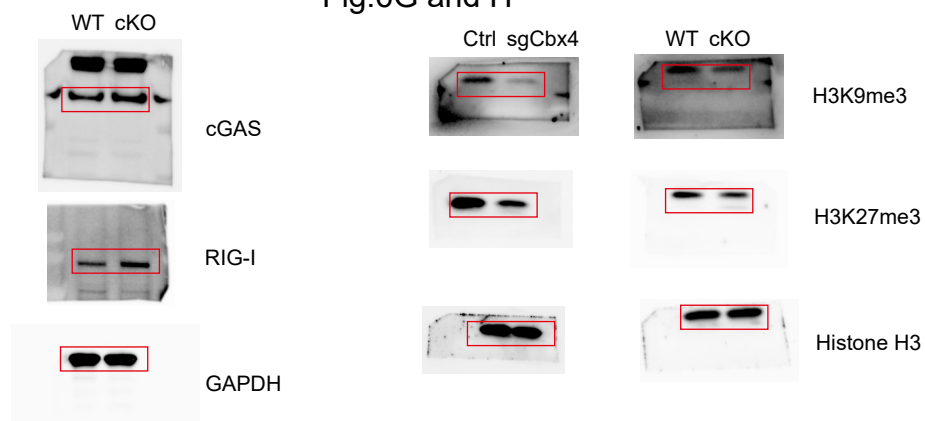

Fig.S2A

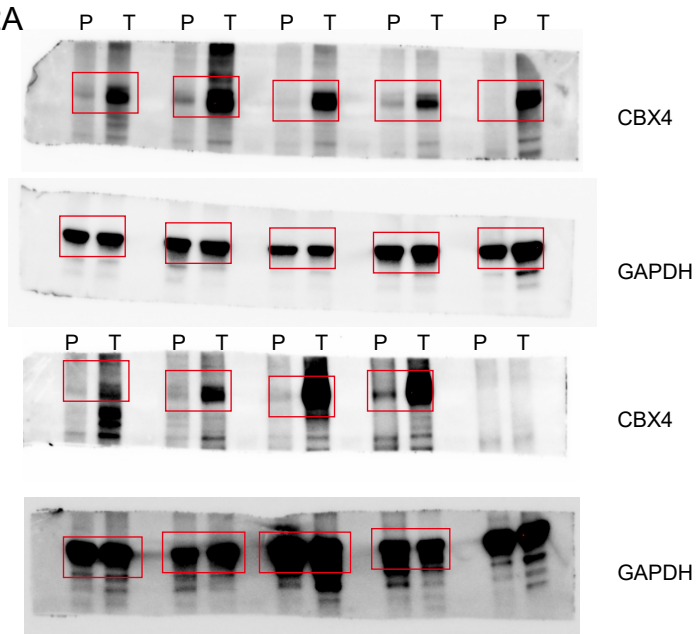

Fig.S4C

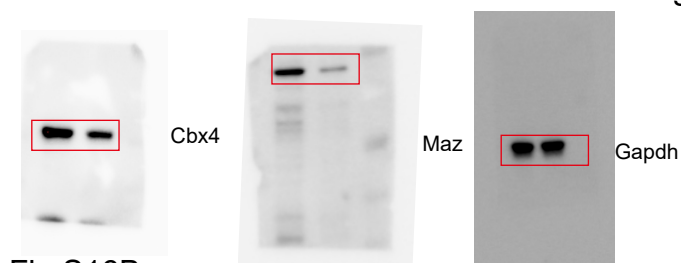

Fig.S4H

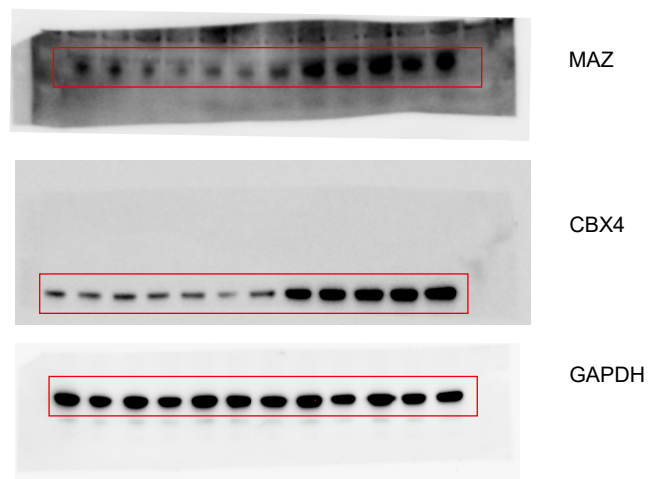

Fig.S16B

Fig.S16C

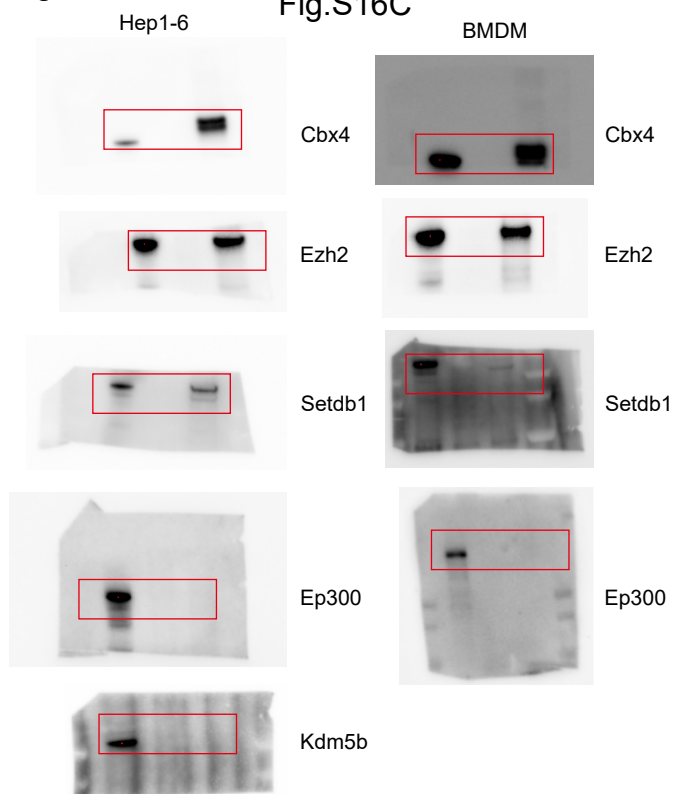

Supplement: Unedited blot and gel images [file jci-136-200564-s266.pdf]
